# Supplementary material for: The comprehensive researcher development framework (CRDF): Core learning outcomes for research training
Source: PLoS One. 2025 Sep 15;20(9):e0332587. doi: 10.1371/journal.pone.0332587 (PMC12435680; doi:10.1371/journal.pone.0332587)
Supplement: S1 Appendix — (PDF) [file pone.0332587.s001.pdf]

S1. Researcher Development Frameworks and Assessments included in Literature Review

	Citation	Discipline(s) Addressed	Training Stage(s) Addressed	Validity Evidence
1	Ahmadi et al. (2022)	Health Professions	Graduate	Content
2	Association of College and Research Libraries (2000)	Cross-Disciplinary	Undergraduate	Content
3	Böttcher & Thiel (2018)	Cross-Disciplinary	Undergraduate; Graduate	Content; Internal Structure
4	Bray & Boon (2011)	Physical Sciences; Life Sciences; Social Sciences; Arts/Humanities	Graduate; Postdoc	Content; Response Processes
5	Brown et al. (2016	Life Sciences	Undergraduate	Content
6	Brownell & Kloser (2015)	Life Sciences	Undergraduate	Content
7	Burke et al. (2005)	Health Professions	Undergraduate; Graduate	Content
8	Butz & Branchaw (2020)	Physical Sciences; Life Sciences	Undergraduate; Graduate	Content; Response Processes; Internal Structure
9	Carnethon et al. (2019)	Health Professions	Postdoc	Content
10	Charumbira et al. (2021)	Health Professions	Undergraduate	Content

	Citation	Discipline(s) Addressed	Training Stage(s) Addressed	Validity Evidence
11	Clemmons et al. (2020)	Life Sciences	Undergraduate	Content; Response Processes; Internal Structure
12	Cui & Harshman (2020)	Physical Sciences	Graduate, Postdoc	Content; Response Processes
13	Dewey et al. (2008)	Social Sciences	Undergraduate; Graduate	Content
14	Drotar et al. (2015)	Health Professions	Postdoc	Criterion
15	Duru & Örsal (2021)	Health Professions	Undergraduate; Graduate	Internal Structure; Convergent
16	Elder et al. (2023)	Physical Sciences; Social Sciences	Undergraduate	Content
17	Enders (2011)	Life Sciences	Graduate	Content
18	Feldon et al. (2017)	Life Sciences	Graduate	Content
19	Feldon et al. (2019)	Life Sciences	Graduate	Content
20	Feldon et al. (2015)	Physical Sciences, Life Sciences	Graduate	Content
21	France et al. (2008)	Health Professions	Graduate	Content
22	Gess et al. (2018)	Social Sciences	Undergraduate	Content; Response Processes; Internal Structure; Test Criterion

	Citation	Discipline(s) Addressed	Training Stage(s) Addressed	Validity Evidence
23	Harsh et al. (2017)	Physical Sciences	Undergraduate	Content: Test Criterion
24	Hayes-Harb et al. (2020)	Cross-Disciplinary	Undergraduate	Content
25	Hodgson et al. (2013)	Health Professions	Graduate	Content
26	Ipanaqué-Zapata et al. (2023)	Cross-Disciplinary	Undergraduate	Content; Internal Structure
27	Kamen et al. (2010)	Health Professions	Graduate	Content; Internal Structure
28	Karyiana et al. (2017)	Social Sciences	Graduate	Content
29	Kiley & Wisker (2009)	Cross-Disciplinary	Graduate	Content
30	Kulikowski et al. (2012)	Life Sciences	Graduate	Content
31	Lambie et al. (2014)	Social Sciences	Graduate	Content
32	Larson et al. (2011)	Cross-Disciplinary	Graduate	Content
33	Lindsay & Floyd (2019)	Social Sciences	Graduate; Postdoc	Content
34	Madan-Swain et al. (2012)	Life Sciences; Health Professions	Graduate	Content
35	Maltese et al. (2017)	Physical Sciences; Life Sciences	Undergraduate	Content; Response Processes; Internal Structure

	Citation	Discipline(s) Addressed	Training Stage(s) Addressed	Validity Evidence
36	Meijers et al. (2005)	Physical Sciences	Undergraduate; Graduate	Content
37	Mekolochick (2021)	Cross-Disciplinary	Undergraduate	Content
38	Miller et al. (2021)	Health Professions	Graduate	Content
39	Musial et al. (2007)	Health Professions	Postdoc	Content
40	National Postdoctoral Association (n.d.)	Physical Sciences; Life Sciences; Social Sciences; Arts/Humanities	Postdoc	Content
41	Nowell et al. (2021)	Cross-Disciplinary	Postdoc	Content
42	Patra & Khan (2019)	Health Professions	Graduate	Content
43	Pelaez et al. (2017)	Life Sciences	Undergraduate	Content
44	Poloyac et al. (2011)	Health Professions	Graduate	Content
45	Qiu et al. (2019)	Health Professions	Undergraduate	Internal Structure; Response Processes
46	Senekal et al. (2022)	Cross-Disciplinary	Graduate	Content
47	Singer et al. (2020).	Physical Sciences; Life Sciences; Social Sciences; Arts/Humanities	Undergraduate	Content; Response Processes; Internal Structure

	Citation	Discipline(s) Addressed	Training Stage(s) Addressed	Validity Evidence
48	Singer & Zimmerman (2012)	Cross-Disciplinary	Undergraduate	Content; Response Processes
49	Steen et al. (2021)	Health Professions	Postdoc	Content
50	Stiers et al. (2015)	Health Professions	Postdoc	Content
51	Swank & Lambie (2016)	Social Sciences	Graduate	Content; Internal Structure
52	Talley (2014)	Law	Graduate	Content
53	Verderame et al. (2018)	Physical Sciences; Life Sciences	Graduate; Postdoc	Content
54	Willison & O'Regan (2007)	Life Sciences	Undergraduate	Content
55	Willison et al. (2018)	Physical Sciences, Life Sciences; Social Sciences; Arts/Humanities	Undergraduate	Content
56	Wilson Sayres et al. (2018)	Life Sciences	Undergraduate	Content

Note: Validity evidence refers to the evidence that a test can be used for its intended purpose. For this paper, we analyzed the different types of validity evidence presented for the content of frameworks and assessments. The different types of validity evidence are defined as follows: Content, or evidence based on test content, is the extent to which the content of a test or assessment relates to its construct. This type of evidence can come from expert review or alignment of items to the different areas to be assessed. Response Processes refers to validity evidence based on how individuals are thinking about and processing individual items. Internal Structure refers to evidence that individual items relate to each other and the dimension(s) of the test in the expected ways. Convergent evidence refers to the extent to which test scores relate to similar constructions on separate tests. Test criterion provides evidence that the test predicts an intended outcome. For more information on different types of validity evidence, see the Standard for Educational and Psychological Testing (AERA, APA, NCME, 2014).

Reference List for Literature Search

Ahmadi M, Sheikhtaheri A, Tahmasbi F, Eslami Jahromi M, Rangraz Jeddi F. A competency framework for Ph.D. programs in health information management. Int J Med Inform. 2022;168: 104906. doi:10.1016/j.ijmedinf.2022.104906

American Library Association. Information Literacy Competency Standards for Higher Education. Jan 2000. Available: https://alair.ala.org/items/294803b6-2521-4a96-a044-96976239e3fb

Böttcher F, Thiel F. Evaluating research-oriented teaching: a new instrument to assess university students' research competences. High Educ. 2018;75: 91–110. doi:10.1007/s10734-017-0128-y

Bray R, Boon S. Towards a framework for research career development: An evaluation of the UK's Vitae Researcher Development Framework. International Journal for Researcher Development. 2011;2: 99–116. doi:10.1108/17597511111212709

Brown AM, Lewis SN, Bevan DR. Development of a structured undergraduate research experience: Framework and implications. Biochem Mol Biol Educ. 2016;44: 463–474. doi:10.1002/bmb.20975

Brownell SE, and Kloser MJ. Toward a conceptual framework for measuring the effectiveness of course-based undergraduate research experiences in undergraduate biology. Stud High Educ. 2015;40: 525–544. doi:10.1080/03075079.2015.1004234

Burke LE, Schlenk EA, Sereika SM, Cohen SM, Happ MB, Dorman JS. Developing Research Competence to Support Evidence-Based Practice. J Prof Nurs. 2005;21: 358–363. doi:10.1016/j.profnurs.2005.10.011

Butz AR, Branchaw JL. Entering Research Learning Assessment (ERLA): Validity Evidence for an Instrument to Measure Undergraduate and Graduate Research Trainee Development. LSE. 2020;19: ar18. doi:10.1187/cbe.19-07-0146

Carnethon MR, Neubauer LC, Greenland P. Competency-Based Postdoctoral Education. Circulation. 2019;139: 310–312. doi:10.1161/CIRCULATIONAHA.118.037494

Charumbira MY, Berner K, Louw QA. Research competencies for undergraduate rehabilitation students : a scoping review. Afr J Health Prof Educ. 2021;13: 52–58. doi:10.7196/AJHPE.2021.v13i1.1229

Clemmons AW, Timbrook J, Herron JC, Crowe AJ. BioSkills Guide: Development and National Validation of a Tool for Interpreting the Vision and Change Core Competencies. LSE. 2020;19: ar53. doi:10.1187/cbe.19-11-0259

Cui Q, Harshman J. Qualitative Investigation to Identify the Knowledge and Skills That U.S.-Trained Doctoral Chemists Require in Typical Chemistry Positions. J Chem Educ. 2020;97: 1247–1255. doi:10.1021/acs.jchemed.9b01027

Dewey JD, Montrosse BE, Schröter DC, Sullins CD, MattoxII JR. Evaluator Competencies: What's Taught Versus What's Sought. Am J Eval. 2008;29: 268–287. doi:10.1177/1098214008321152

Drotar D, Cortina S, Crosby LE, Hommel KA, Modi AC, Pai ALH. Competency-based postdoctoral research training for clinical psychologists: An example and implications. Train Educ Prof Psychol. 2015;9: 92–98. doi:10.1037/tep0000032

Duru P, Örsal Ö. Development of the Scientific Research Competency Scale for nurses. J Res Nurs. 2021;26: 684–700. doi:10.1177/17449871211020061

Elder S, Wittman H, Giang A. Building sustainability research competencies through scaffolded pathways for undergraduate research experience. Elementa (Wash D C). 2023;11: 00091. doi:10.1525/elementa.2022.00091

Enders F. Evaluating Mastery of Biostatistics for Medical Researchers: Need for a New Assessment Tool. Clin Transl Sci. 2011;4: 448–454. doi:10.1111/j.1752-8062.2011.00323.x

Feldon DF, Rates ,Christopher, and Sun C. Doctoral conceptual thresholds in cellular and molecular biology. Int J Sci Educ. 2017;39: 2574–2593. doi:10.1080/09500693.2017.1395493

Feldon DF, Litson K, Jeong S, Blaney JM, Kang J, Miller C, et al. Postdocs' lab engagement predicts trajectories of PhD students' skill development. Proc Natl Acad Sci U S A. 2019;116: 20910–20916. doi:10.1073/pnas.1912488116

Feldon DF, Maher MA, Hurst M, Timmerman B. Faculty Mentors', Graduate Students', and Performance-Based Assessments of Students' Research Skill Development. Am Educ Res J. 2015;52: 334–370. doi:10.3102/0002831214549449

France CR, Masters KS, Belar CD, Kerns RD, Klonoff EA, Larkin KT, et al. Application of the competency model to clinical health psychology. Prof Psychol Res Pr. 2008;39: 573–580. doi:10.1037/0735-7028.39.6.573

Gess C, Geiger C, Ziegler M. Social-Scientific Research Competency. Eur J Psychol Assess. 2019;35: 737–750. doi:10.1027/1015-5759/a000451

Harsh J, J. Esteb J, V. Maltese A. Evaluating the development of chemistry undergraduate researchers' scientific thinking skills using performance-data: first findings from the performance assessment of undergraduate research (PURE) instrument. Chem Educ Res Pract. 2017;18: 472–485. doi:10.1039/C6RP00222F

Hayes-Harb R, St. Andre M, Shannahan M. Assessment of Undergraduate Research Learning Outcomes: Poster Presentations as Artifacts. SPUR. 2020;3: 55–61. doi:10.18833/spur/3/4/10

Hodgson JL, Pelzer JM, Inzana KD. Beyond NAVMEC: Competency-Based Veterinary Education and Assessment of the Professional Competencies. J Vet Med Educ. 2013;40: 102–118. doi:10.3138/jvme.1012-092R

Ipanaqué-Zapata M, Figueroa-Quiñones J, Bazalar-Palacios J, Arhuis-Inca W, Quiñones-Negrete M, Villarreal-Zegarra D. Research skills for university students' thesis in E-learning: Scale development and validation in Peru. Heliyon. 2023;9: e13770. doi:10.1016/j.heliyon.2023.e13770

Kamen C, Veilleux JC, Bangen KJ, VanderVeen JW, Klonoff EA. Climbing the stairway to competency: Trainee perspectives on competency development. Train Educ Prof Psychol. 2010;4: 227–234. doi:10.1037/a0021092

Kariyana I, Sonn ,Reynold A., and Marongwe N. Objectivity of the subjective quality: Convergence on competencies expected of doctoral graduates. Cheng M, editor. Cogent Educ. 2017;4: 1390827. doi:10.1080/2331186X.2017.1390827

Kiley M, and Wisker G. Threshold concepts in research education and evidence of threshold crossing. High Educ Res Dev. 2009;28: 431–441. doi:10.1080/07294360903067930

Kulikowski CA, Shortliffe EH, Currie LM, Elkin PL, Hunter LE, Johnson TR, et al. AMIA Board white paper: definition of biomedical informatics and specification of core competencies for

graduate education in the discipline. JAMIA Open. 2012;19: 931–938. doi:10.1136/amiajnl-2012-001053

Lambie GW, Hayes BG, Griffith C, Limberg D, Mullen PR. An Exploratory Investigation of the Research Self-Efficacy, Interest in Research, and Research Knowledge of Ph.D. in Education Students. Innov High Educ. 2014;39: 139–153. doi:10.1007/s10755-013-9264-1

Larson EL, Landers TF, Begg MD. Building Interdisciplinary Research Models: A Didactic Course to Prepare Interdisciplinary Scholars and Faculty. Clin Transl Sci. 2011;4: 38–41. Available: https://ascpt.onlinelibrary.wiley.com/doi/10.1111/j.1752-8062.2010.00258.x

Lindsay H, Floyd A. Experiences of using the researching professional development framework. Stud Grad Postdr Educ. 2019;10: 54–68. doi:10.1108/SGPE-02-2019-049

Madan-Swain A, Hankins SL, Gilliam MB, Ross K, Reynolds N, Milby J, et al. Applying the Cube Model to Pediatric Psychology: Development of Research Competency Skills at the Doctoral Level. J Pediatr Psychol. 2012;37: 136–148. doi:10.1093/jpepsy/jsr096.

Maltese A, Harsh J, Jung E. Evaluating Undergraduate Research Experiences—Development of a Self-Report Tool. Educ Sci (Basel). 2017;7: 87. doi:10.3390/educsci7040087

Meijers AWM, Borghuis VAJ, Mutsaers EJPJ, Overveld van CWAM, Perrenet JC. Criteria for academic bachelor’s and master’s curricula. 2e, gew. dr. ed. Eindhoven: Technische Universiteit Eindhoven; 2005.

Mekolichick J. Mapping the Impacts of Undergraduate Research, Scholarship, and Creative Inquiry Experiences to the NACE Career Readiness Competencies. NACE Journal. 2021;82: 34–40. Available: https://ebiztest.naceweb.org/career-readiness/competencies/mapping-the-impacts-of-undergraduate-research-scholarship-and-creative-inquiry-experiences-to-the-nace-career-readiness-competencies/

Miller L, Brushett S, Ayn C, Furlotte K, Jackson L, MacQuarrie M, et al. Developing a Competency Framework for Population Health Graduate Students Through Student and Faculty Collaboration. Pedagogy Health Promot. 2021;7: 280–288. doi:10.1177/2373379919859607

Musial JL, Rubinfeld IS, Parker AO, Reickert CA, Adams SA, Rao S, et al. Developing a Scoring Rubric for Resident Research Presentations: A Pilot Study. J Surg Res. 2007;142: 304–307. doi:10.1016/j.jss.2007.03.060

Nowell L, Dhingra S, Kenny N, Jacobsen M, Pexman P. Professional learning and development framework for postdoctoral scholars. Stud Grad Postdr Educ. 2021;12: 353–370. doi:10.1108/SGPE-10-2020-0067

NPA Core Competencies. [cited 25 Apr 2025]. Available: https://www.nationalpostdoc.org/page/CoreCompetencies

Patra S, Khan AM. Development and implementation of a competency-based module for teaching research methodology to medical undergraduates. J Educ Health Promot. 2019;8: 164. doi:10.4103/jehp.jehp_133_19

Pelaez N, Anderson T, Gardner S, Yin Y, Abraham J, Bartlett E, et al. The Basic Competencies of Biological Experimentation: Concept-Skill Statements. PIBERG Instructional Innovation Materials. 2016. Available: https://docs.lib.purdue.edu/pibergiim/4

Poloyac SM, Empey KM, Rohan LC, Skledar SJ, Empey PE, Nolin TD, et al. Core Competencies for Research Training in the Clinical Pharmaceutical Sciences. Am J Pharm Educ. 2011;75: 27. doi:10.5688/ajpe75227

Qiu C, Feng X, Reinhardt JD, Li J. Development and psychometric testing of the Research Competency Scale for Nursing Students: An instrument design study. Nurse Educ Today. 2019;79: 198–203. doi:10.1016/j.nedt.2019.05.039

Senekal JS, Munnik E, Frantz JM. A systematic review of doctoral graduate attributes: Domains and definitions. Front Educ. 2022;7. doi:10.3389/feduc.2022.1009106

Singer J, Weiler D, Zimmerman B, Fox S, Ambos E. Assessment in Undergraduate Research. Earth Sciences Faculty Publications. 2022. doi:https://doi.org/10.1017/9781108869508

Singer J, Zimmerman B. Evaluating a Summer Undergraduate Research Program: Measuring Student Outcomes and Program Impact. Counc Undergrad Res Q. 2012;32: 40–47. Available: https://digitalcommons.buffalostate.edu/cgi/viewcontent.cgi?article=1004&context=earth_sciences_facpub

Steen K, Vornhagen J, Weinberg ZY, Boulanger-Bertolus J, Rao A, Gardner ME, et al. A structured professional development curriculum for postdoctoral fellows leads to recognized knowledge growth. PLoS ONE. 2021;16: e0260212. doi:10.1371/journal.pone.0260212

Stiers W, Barisa M, Stucky K, Pawlowski C, Van Tubbergen M, Turner AP, et al. Guidelines for competency development and measurement in rehabilitation psychology postdoctoral training. Rehabil Psychol. 2015;60: 111–122. doi:10.1037/a0038353

Swank JM, Lambie GW. Development of the Research Competencies Scale. Meas Eval Couns Dev. 2016;49: 91–108. doi:10.1177/0748175615625749

Talley NB. Are You Doing It Backward? Improving Information Literacy Instruction Using the AALL Principles and Standards for Legal Research Competency, Taxonomies, and Backward Design. Law Libr J. 2014;106: 47–68. Available: https://heinonline.org/HOL/P?h=hein.journals/llj106&i=47

Verderame MF, Freedman VH, Kozlowski LM, McCormack WT. Competency-based assessment for the training of PhD students and early-career scientists. Pewsey E, editor. eLife. 2018;7: e34801. doi:10.7554/eLife.34801

Willison J, O'Regan K. Commonly known, commonly not known, totally unknown: a framework for students becoming researchers. High Educ Res Dev. 2007;26. doi:10.1080/07294360701658609

Willison J, O'Regan K, Kuhn SK. Researcher Skill Development Framework (US English Edition). 2018.

Wilson Sayres MA, Hauser C, Sierk M, Robic S, Rosenwald AG, Smith TM, et al. Bioinformatics core competencies for undergraduate life sciences education. PLoS ONE. 2018;13: e0196878. doi:10.1371/journal.pone.0196878
