# Supplementary material for: The comprehensive researcher development framework (CRDF): Core learning outcomes for research training
Source: PLoS One. 2025 Sep 15;20(9):e0332587. doi: 10.1371/journal.pone.0332587 (PMC12435680; doi:10.1371/journal.pone.0332587)
Supplement: S3 Appendix — (PDF) [file pone.0332587.s004.pdf]

S3a. Code Themes, Definitions & Assignments

Themes	Elements with this theme will describe:	Count of Assigned	Pct of Assigned
Aims, RQs & Hypotheses	Conceptualizing a research project, including aims, research questions or hypotheses	64	4.46%
Authorship	Requirements for authorship, authorship order, negotiating authorship. etc.	4	0.28%
Budget Management	Managing finances relating to supporting research (developing budgets, identifying funding opportunities, etc.)	11	0.77%
Career development	Exploring career opportunities, translating skills, engaging in professional development	10	0.70%
Collaboration	Collaborating with team/research members and others on research and cultivating relationships in a research context (e.g., identifying other professionals in the field with similar interests)	31	2.16%
Communicating research & research ideas	Communicating about the research in different ways (e.g., written, oral, visual, or in a grant proposal)	126	8.78%
Confidence	Confidence to do research successfully	6	0.42%
Creative thinking	Thinking creatively about research or use of creativity in research; innovation	12	0.84%
Critical evaluation - content knowledge	Critical evaluation of disciplinary content knowledge, evaluating information, identifying gaps	48	3.34%
Critical thinking	Thinking critically about research (both their own and others) and evaluating research	11	0.77%
Cultural context of research	How research occurs in the context of societies or cultures or how society/culture influences research	8	0.56%
Culturally aware/relevant research	Incorporating cultural relevance into research approaches or practices	4	0.28%
Curiosity	Curiosity about research	4	0.28%
Data analysis approaches	Knowing how to analyze data using the correct approach/technique (e.g., computational, quantitative, qualitative)	68	4.74%
Disciplinary approaches	Knowing the variety/types of methods used to investigate research questions in the discipline. Thinking about, determining methods, determining approach for research	194	13.52%
Disciplinary content knowledge	General knowledge within the research discipline, including key terms, theories, concepts and common pratices.	108	7.53%
Effective interpersonal research relationships	Use of interpersonal skills with colleagues in research, including networking	28	1.95%
General research beliefs	General beliefs about research that don't belong to a specific construct	3	0.21%
Give feedback on research	Giving feedback on research effectively (e.g., Peer review)	6	0.42%
Grit	Resilience and perseverance	11	0.77%
Identity	Identifying as a researcher or developing research identity; establishing expertise in one's field	4	0.28%
Inclusive research practice	Working inclusively and effectively with individuals of different identities	14	0.98%
Independence	Developing independence as a researcher	11	0.77%
Inferences and Implications	Makes meaning of research results (e.g., synthesizing results, discussing results within the context of a theory, model or framework, or using results to refine theories, frameworks, and models)	73	5.09%
Interpret results	Interpreting the results of analyses and using them to confirm or reject hypotheses or answer research questions.	34	2.37%
Leadership	General leadership in research - strategic planning/goal setting	17	1.18%
Mentoring	Mentoring or Teaching others in the research context (not classroom teaching)	10	0.70%

Themes	Elements with this theme will describe:	Count of Assigned	Pct of Assigned
N/A - Heading	Used for headings of frameworks where skills appear underneath	48	3.34%
Omit	Any skills listed that do not relate to research. (e.g., teaching, professional skills in other contexts) or are too vague, or for which not enough information is provided to definitively code them	55	3.83%
Outreach	Engaging with the broader community about research; beyond the academy; ambassadorship	14	0.98%
Practical research skills/techniques	Refers to using the technical skills and/or software or tools needed to do research in a discipline (e.g., techniques, knowing how to use tools,etc.)	79	5.51%
Problem solving	Solving problems in the context of doing research	18	1.25%
Professionalism	Identifying and following cultural and professional norms/expectations within the discipline & the team. Conducting yourself professionally as an individual	18	1.25%
Project management	Management of research projects (either individual project or team projects)	25	1.74%
Receive research feedback	Interpret and respond to feedback from others	7	0.49%
Record keeping and data storage	Ways to responsibly store and record research data/ records	15	1.05%
Responsible and ethical research conduct	General RCR skills and/or research ethics	81	5.64%
Safety	Knowing and implementing research safety practices	2	0.14%
Search disciplinary literature/databases	Knowing how to search the literature or databases relevant to their discipline	46	3.21%
Self Reflective	Self-awareness, thinking reflectively about oneself with respect to the research	14	0.98%
Self-Regulation in Research	Managing time, emotions and behaviors in the research environment	3	0.21%
Team management	Management of a research team	13	0.91%
Time Management	Time management and individual project management, self-management skills	6	0.42%
Translating research to practice	Translating research to a broader audience and/or a real-world context (e.g., to practitioners or across disciplines)	23	1.60%
Use and test disciplinary theories, frameworks, and models	Use and development of theories, frameworks and/or models that are relevant to their discipline.	33	2.30%
Visualize data	Representing Data in a visual way (e.g., charts, graphs, visual models, etc.)	12	0.84%
Wellness	Practices to support well-being in research	2	0.14%

S3b. Framework Elements & Code Themes

Framework	Element	Theme
2	2.1 Broad Conceptual Knowledge of a Scientific Discipline	N/A - Heading
2	2.1.1 Knowledge base for multiple disciplines aquired from classes, seminars, journal clubs, etc.	Disciplinary content knowledge
2	2.1.2 Broad scientific approaches	Disciplinary approaches
2	2.2 Deep Knowledge of a Specific Field	N/A - Heading
2	2.2.1 Historical Context of a Specific Area	Disciplinary content knowledge
2	2.2.2 Current Content Expertise in the Specific Area	Disciplinary content knowledge
2	2.2.3 Tools and Approaches for a Specific Area	Disciplinary approaches
2	2.3 Critical Thinking Skills	N/A - Heading
2	2.3.1 Recognize Important Questions	Disciplinary content knowledge
2	2.3.2 Design a Single Experiment (answer questions, controls, etc.)	Aims, RQs & Hypotheses
2	2.3.3 Interpret Data	Interpret results
2	2.3.4 Design a Research Program	Aims, RQs & Hypotheses
2	2.4 Experimental Skills	N/A - Heading
2	2.4.1 Identify Appropriate Experimental Protocols	Disciplinary approaches
2	2.4.2 Design and Execute Experimental Protocols	Disciplinary approaches
2	2.4.3 Identify and Troubleshoot Technical Issues	Problem solving
2	2.4.4 Lab Safety and Regulatory Issues	Safety
2	2.4.5 Research Records and Data Storage	Record keeping and data storage
2	2.4.6 Recognition of Data Ownership	Responsible and ethical research conduct
2	2.5 Computational Skills	N/A - Heading
2	2.5.1 Basic Statistical Analysis	Data analysis approaches
2	2.5.2 Bioinformatics Literacy	Disciplinary content knowledge
2	2.6 Collaboration and Team Science	N/A - Heading
2	2.6.1 Openness to Collaboration	Collaboration
2	2.6.2 Self-Awareness	Self Reflective
2	2.6.3 Disciplinary Awareness	Disciplinary content knowledge
2	2.6.4 Integration	Inferences and Implications
2	2.6.5 Team Skills	Collaboration
2	2.7 Responsible Conduct of Research and Research Ethics	N/A - Heading
2	2.7.1 Knowledge About Responsible Conduct of Research (RCR)	Responsible and ethical research conduct
2	2.7.2 Ethical Decision Making (EDM) in RCR (outcome to process)	Responsible and ethical research conduct
2	2.7.3 Moral Courage	Responsible and ethical research conduct
2	2.7.4 Integrity	Responsible and ethical research conduct
2	2.8 Communication Skills	N/A - Heading
2	2.8.1 Informal Oral Presentation Skills	Communicating research & research ideas
2	2.8.2 Formal Oral Presentation Skills	Communicating research & research ideas
2	2.8.3 Written Communication - Scientific Manuscript	Communicating research & research ideas
2	2.8.4 Written Communication - Grant Proposals	Communicating research & research ideas
2	2.8.5 Written Communication - Meeting Poster	Communicating research & research ideas
2	2.8.6 Communication With the Public	Communicating research & research ideas
2	2.9 Leadership Skills	N/A - Heading
2	2.9.1 Vision	Leadership
2	2.9.2 Integrity	Responsible and ethical research conduct
2	2.9.3 Group Dynamics and Interpersonal Skills	Effective interpersonal research relationships

Framework	Element	Theme
2	2.9.4 Organization and Planning	Project management
2	2.9.5 Decision-Making	Leadership
2	2.9.6 Problem-Solving	Problem solving
2	2.9.7 Managing Conflicts	Effective interpersonal research relationships
2	2.10 Survival Skills	N/A - Heading
2	2.10.1 Motivation	General research beliefs
2	2.10.2 Perseverance	Grit
2	2.10.3 Adaptability	Grit
2	2.10.4 Professional Development	Career development
2	2.10.5 Networking	Effective interpersonal research relationships
3	3.1 Embark & Clarify: Respond to or initiate research and clarify or determine what knowledge is required, heeding ethical, cultural, social and team (ECST) considerations.	Critical evaluation - content knowledge
3	3.2 Find & Generate: Find and generate needed information/data using appropriate methodology	Disciplinary approaches
3	3.3a Evaluate: Determine and critique the degree of credibility of selected sources, information, and of data generated.	Critical evaluation - content knowledge
3	3.3b Reflect: Metacognitively reflect on processes used.	Critical evaluation - content knowledge
3	3.4a Organize: Organize information and data to reveal patterns and themes	Data analysis approaches
3	3.4b Manage: manage teams and research processes.	Team management
3	3.5a Analyze:Analyze information/data critically and	Data analysis approaches
3	3.5b Synthesize: synthesize new knowledge to produce coherent individual/team understandings.	Inferences and Implications
3	3.6a Communicate: Discuss, listen, write, present, and perform the processes, understandings and applications of the research	Communicating research & research ideas
3	3.6b Communicate:respond to feedback, accounting for ethical, cultural, social, and team (ECST) issues.	Receive research feedback
4	4.1 Disciplinary-Specific Knowledge	N/A - Heading
4	4.1.1 Analytical Approach to Defining Scientific Questions	Aims, RQs & Hypotheses
4	4.1.2 Design of Scientifically Testable Hypotheses	Aims, RQs & Hypotheses
4	4.1.3 Broad Based and Cross-Disciplinary Knowledge Acquisition	Disciplinary content knowledge
4	4.1.4 Interpretation and Analysis of Data	Interpret results
4	4.2 Research Skill Development	N/A - Heading
4	4.2.1 Research Techniques and Laboratory Safety	Practical research skills/techniques
4	4.2.2 Experimental Design	Disciplinary approaches
4	4.2.3 Data Analysis and Interpretation	Data analysis approaches
4	4.2.4 Statistical Analysis	Data analysis approaches
4	4.2.5 Effective Search Strategies and Critical Evaluation of the Literature	Search disciplinary literature/databases
4	4.2.6 Principles of Peer Review Process	Give feedback on research
4	4.3 Communication Skills	N/A - Heading
4	4.3.1 Writing - Scientific Publications	Communicating research & research ideas
4	4.3.2 Writing - Grants/Applications (Government, Corporations, Foundations)	Communicating research & research ideas
4	4.3.3 Writing - Career (CV and Resume, Cover Letters, Research and Teaching Statements or Portfolios, Letters of Recommendation or Collaboration)	Communicating research & research ideas
4	4.3.4 Speaking - Research Presenations (Poster Sessions, Conferences/Seminars, PowerPoint Presentations)	Communicating research & research ideas
4	4.3.5 Speaking - The Job Interview (Job Talks)	Communicating research & research ideas

Framework	Element	Theme
4	4.3.6 Teaching - Teaching Methods	Omit
4	4.3.7 Teaching - Learning Styles	Omit
4	4.3.8 Interpersonal Communication Skills - Style, Tone and Non-Verbal Cues	Effective interpersonal research relationships
4	4.3.9 Interpersonal Communication Skills - Negotiation	Effective interpersonal research relationships
4	4.3.10 Interpersonal Communication Skills - Performance Reviews/Feedback	Team management
4	4.3.11 Interpersonal Communication Skills - Difficult Conversations/Minimizing Conflict	Effective interpersonal research relationships
4	4.3.12 Special Situations - Networking	Effective interpersonal research relationships
4	4.3.13 Special Situations - Conflict Resolution	Effective interpersonal research relationships
4	4.3.14 Special Situations - Managing the News Media	Communicating research & research ideas
4	4.4 Workplace Professionalism: Developing social skills and an inclusive environment as a member of a research team or among different teams through effectie and respectful interactions with diverse students, employees, peers, and supervisors	Omit
4	4.4.1 Assess and uphold workplace etiquette, performance standards, and project goals with members of a team or in other immediate work environments	Professionalism
4	4.4.2 Respect, evaluate, and enhance the intellectual contribution of others by encouraging, nourishing attitudes and relationships	Team management
4	4.4.3 Allow others the right to self-expression and opinion to deeply understand and respect the complext identities of others, their histories, and their cultures	Inclusive research practice
4	4.4.4 Preserve and promote mentoring relationships within the workplace	Mentoring
4	4.4.5 Develop confidence in expressing one's attitudes, observations, or opinions using positive or reinforcing language	Confidence
4	4.5 Institutional Professionalism: Connecting as an employee or representative of an institution by developing advocacy skills with and seeking assistance from compliance officers, senior management, or administration (at an institution, across institutions, or with funding stakeholders)	Omit
4	4.5.1 Compliance with rules and regulations	Responsible and ethical research conduct
4	4.5.2 Respect, adjust, and comply with unit-specific and institutional norms and expectations of appearance, conduct, and values	Professionalism
4	4.5.3 Collaborate with senior management and administration through participation on institutional committees or other internal leadership opportunitites	Leadership
4	4.6 Collegial Professionalism: Engaging colleagues, collaborators, and experts as a citizen to scholarship to maintain the intellectual integrity of the discipline and the profession	Omit
4	4.6.1 Identify, establish, and maintain effective and trustworthy partnerships with mentors and collaborators	Effective interpersonal research relationships
4	4.6.1.1 Informal communication (networking and follow-through)	Effective interpersonal research relationships
4	4.6.1.2 Respecting others' time and needs in completing goals	Professionalism
4	4.6.1.3 Data confidentiality and ownership	Responsible and ethical research conduct
4	4.6.1.4 Developing rules, expectations, and evaluations for collaborators	Collaboration
4	4.6.1.5 Peer-review of manuscripts, grant proposals, and competitive awards	Give feedback on research
4	4.6.2 Advance and promote the discipline by participating in public and professional service activities, such as professional societies, editorial and advisory boards, peer review panels, and institutional committees	Outreach

Framework	Element	Theme
4	4.6.3 Advance and promote the discipline by participating in partnerships with government agencies, foundations, and/or non-profit organizations, such as funding agency grant panels or other advocacy/advisory boards to contribute to the advancement and promotion of the discipline	Outreach
4	4.6.4 Identify and manage apparent and actual conflicts of interest, ethical violations, and violoations of expected professional behavior	Responsible and ethical research conduct
4	4.7 Universal Professionalism: Bearing responsibilty for the universal perception of the profession and his/her contribution towards social justice or improvement as a representative of the community	Omit
4	4.7.1 Engage and serve the general public (schools, government officials, other professions, media) to promote the profession	Outreach
4	4.7.2 Identify societal needs and communicate the impact of the profession to address those needs to the public's benefit	Outreach
4	4.8 Leadership and Management	N/A - Heading
4	4.8.1 Research Staff Management - Writing job description	Team management
4	4.8.2 Research Staff Management - Recruitment	Team management
4	4.8.20 Leadership - Serving as a Role Model	Leadership
4	4.8.3 Research Staff Management - Hiring/Terminating	Team management
4	4.8.4 Research Staff Management - Mentoring/Retention	Mentoring
4	4.8.5 Research Staff Management - Performance reviews/feedback	Team management
4	4.8.6 Research Staff Management - Working with individuals of diverse gender, ethnic, cultural, and religious backgrounds	Inclusive research practice
4	4.8.7 Research Staff Management - Conflict management/difficult conversations	Effective interpersonal research relationships
4	4.8.8 Project Management - Establishing priorities	Project management
4	4.8.9 Project Management - Time management	Project management
4	4.8.10 Project Management Collaborations (intra/interlab)	Collaboration
4	4.8.11 Project Management - Planning - Development of overall plan	Aims, RQs & Hypotheses
4	4.8.12 Project Management - Planning - Strategic planning	Aims, RQs & Hypotheses
4	4.8.13 Project Management - Developing/managing budgets	Budget Management
4	4.8.14 Project Management - Tracking material and equipment use	Project management
4	4.8.15 General Management - Running a meeting	Project management
4	4.8.16 General Management - Delegating responsibilities	Team management
4	4.8.17 Leadership - Idenfitying and Clarifying Goals	Leadership
4	4.8.18 Leadership - Motivating/Inspiring Others - Understanding the long-term strategic vision and helping others to see where their work/roles fit in this picture	Leadership
4	4.8.19 Leadership - Motivating/Inspiring Others - Understanding how to use appropriate leadership styles in any given situation	Leadership
4	4.9 Responsible Conduct of Research	N/A - Heading
4	4.9.1 Data Ownership and Sharing - Sharing of data with collaborators, including industry-specific concerns as appropriate	Responsible and ethical research conduct
4	4.9.2 Data Ownership and Sharing - Ownership and access to data, particularly once a postdoctoral fellow's appointment ends	Responsible and ethical research conduct
4	4.9.3 Data Ownership and Sharing - Legal ramifications of intellectual property, patents and copyright	Responsible and ethical research conduct
4	4.9.4 Publication Practices and Responsible Authorship - Criteria for authorship	Authorship

Framework	Element	Theme
4	4.9.5 Publication Practices and Responsible Authorship - The elements of responsible publication	Authorship
4	4.9.6 Research With Human Subjects - Ethical principles for conducting research with human subjects	Responsible and ethical research conduct
4	4.9.7 Research With Human Subjects - Informed consent and subject confidentiality	Responsible and ethical research conduct
4	4.9.8 Research With Human Subjects - Institutional Review Boards	Responsible and ethical research conduct
4	4.9.9 Research With Human Subjects - Reporting clinical trials	Responsible and ethical research conduct
4	4.9.10 Research Involving Animals - Ethical principles and federal policies for conducting research with animals	Responsible and ethical research conduct
4	4.9.11 Research Involving Animals - Understanding the Three Rs: Replace, Reduce, and Refine animal use in research	Responsible and ethical research conduct
4	4.9.12 Research Involving Animals - Institutional Animal Care and Use Committee (IACUC)	Responsible and ethical research conduct
4	4.9.13 Identifying and Mitigating Research Misconduct - Definitions (federal, ORI/PHS, NASA, NEH, NSF, etc.)	Responsible and ethical research conduct
4	4.9.14 Identifying and Mitigating Research Misconduct - Reporting procedures	Responsible and ethical research conduct
4	4.9.15 Identifying and Mitigating Research Misconduct - The role and risks of being a whistleblower	Responsible and ethical research conduct
4	4.9.16 Conflicts of Interest - Personal and intellectual	Responsible and ethical research conduct
4	4.9.17 Conflicts of Interest - Conflicts of commitment	Responsible and ethical research conduct
4	4.9.18 Conflicts of Interest - Financial conflicts	Responsible and ethical research conduct
4	4.9.19 Conflicts of Interest - Profits and intellectual property rights	Responsible and ethical research conduct
4	4.9.20 Conflicts of Interest - Confidentiality and bias in peer review	Responsible and ethical research conduct
4	4.9.21 Conflicts of Interest - Conflicts and potential competition between mentor and trainee	Responsible and ethical research conduct
5	5.1 The ability to identify gaps or limitations in current research knowledge through the review, filtering, and synthesis of relevant literature.	N/A - Heading
5	5.1.1 Find appropriate sources of relevant scientific information (primary, secondary, etc.)	Search disciplinary literature/databases
5	5.1.2 Filter and evaluate the relevance of information from appropriate sources to the specific research focus	Critical evaluation - content knowledge
5	5.1.3 Evaluate background information with critical scientific skepticism	Critical evaluation - content knowledge
5	5.1.4 Synthesize and apply current knowledge to generate a contextual foundation for the research problem	Aims, RQs & Hypotheses
5	5.1.5 Reflect on the skills and knowledge needed in the relevant field before proceeding to do research	Disciplinary approaches
5	5.1.6 Recognize a gap in current scientific knowledge that can be addressed with experimentation	Critical evaluation - content knowledge
5	5.1.7 Reflect on limits of background knowledge related to the gap (in knowledge)	Critical evaluation - content knowledge
5	5.1.8 Identify a problem that is timely, relevant, and interesting, and, if addressed, could build on our foundational knowledge of science.	Aims, RQs & Hypotheses
5	5.2 The ability to generate a research question and formulate hypotheses.	N/A - Heading
5	5.2.1 Apply systematic observations to discern variable properties of components of biological systems	Practical research skills/techniques
5	5.2.2 Compare observations to existing knowledge, models, or theories	Use and test disciplinary theories, frameworks, and models

Framework	Element	Theme
5	5.2.3 Develop novel, relevant, and testable research questions based on patterns or properties of components observed in biological sytems or described in primary literature	Aims, RQs & Hypotheses
5	5.2.4 Evaluate ethical, theoretical, practical, and cost constraints associated with a research question	Critical evaluation - content knowledge
5	5.2.5 Develop a model (i.e. an abstraction or simplification: an equation, computer simulation, conceptual drawing, or other explanatory representation that show key elements and their relationships) to approximate or represent the behavior of a natural phenomenon	Use and test disciplinary theories, frameworks, and models
5	5.2.6 Articulate the assumptions and limitation of a model	Use and test disciplinary theories, frameworks, and models
5	5.2.7 Evaluate a model to identify ways to improve it	Use and test disciplinary theories, frameworks, and models
5	5.2.8 Use a model (i.e. an abstraction or simplification: an equation, computer simulation, conceptual drawing, or other explanatory representation that show key elements and their relationships) to generate new hypotheses	Use and test disciplinary theories, frameworks, and models
5	5.2.9 Generate multiple explanations of the natural world that are testable and potentially falsifiable	Aims, RQs & Hypotheses
5	5.2.10 Predict associations between treatment conditions and outcome variables for the research target	Aims, RQs & Hypotheses
5	5.2.11 Determine whether multiple hypotheses are mutually exclusive and based on predictions of a model	Use and test disciplinary theories, frameworks, and models
5	5.3 The ability to plan feasible and ethical experiments to answer research questions or test hyphtheses.	N/A - Heading
5	5.3.1 Diagram/catoon the steps of an experimental method	Disciplinary approaches
5	5.3.2 Construct a visual representation (e.g. a graph or diagram) of predicted results	Visualize data
5	5.3.3 Diagram, label and title components for a proposal to conduct an experiment	Communicating research & research ideas
5	5.3.4 Identify assumptions of the different types of experimental designs (manipulative, observational/discovery, natural)	Disciplinary approaches
5	5.3.5 Choose the most appropriate design approach to answer the research question(s)raised	Disciplinary approaches
5	5.3.6 Propose measurable outcomes that would support or refute hypotheses	Disciplinary approaches
5	5.3.7 Optimize treatments for efficiency	Disciplinary approaches
5	5.3.8 Identify potential sources of systemiatic and random error	Disciplinary approaches
5	5.3.9 Draw a timeline of experimental procedures	Project management
5	5.3.10 Identify relevant, measurable variables for testing hypothesis	Disciplinary approaches
5	5.3.11 Identify dependent and independent variables	Disciplinary approaches
5	5.3.12 Idenfity confounding, and/or covariate variables aligned with experiment	Disciplinary approaches
5	5.3.13 Design controls to anticipate likely sources of error to allow for comparison with experimental treatment groups in the context of the experiment	Disciplinary approaches
5	5.3.14 Select appropriate positive and negative controls to define an expected range of outcomes and to allow for comparison with outcomes of experimental treatments	Disciplinary approaches
5	5.3.15 Consider what conditions are necessary to perform the experiments	Disciplinary approaches
5	5.3.16 Randomize the order in which experimental subjects or units experience treatment or control conditions as a way to reduce th chance of bias in the experiment	Disciplinary approaches
5	5.3.17 Explain the implications of a control that did not show the expected result	Inferences and Implications
5	5.3.18 Choose appropriate measurements based on available equipment, population/species, natural variation, and research question(s)	Disciplinary approaches

Framework	Element	Theme
5	5.3.19 Align variables appropriately with measurement tools/scales/instruments	Disciplinary approaches
5	5.3.20 Recognize the limitations of measurement tools/equipment	Disciplinary approaches
5	5.3.21 Identify a target population(s) (might be molecules, cells, organisms, or populations) for the planned experiment	Disciplinary approaches
5	5.3.22 Design the sampling strategy to expose and account for natural variation and measurement error	Disciplinary approaches
5	5.3.23 Align sampling protocol with the research question or hypothesis	Disciplinary approaches
5	5.3.24 Sample subjects randomly for control and treatment groups to reduce the efffect of unanticipated variables	Disciplinary approaches
5	5.3.25 Differentiate between measurement variability and system variablility (natural variation or heterogenous populations)	Disciplinary approaches
5	5.3.26 Determine replication or repeatability needed to quantify variation	Disciplinary approaches
5	5.3.27 Integrate professional and community ethics into research design	Responsible and ethical research conduct
5	5.3.28 Submit planned research to the Institutional Review Board or Animal Care and Use Committee for evaluation, as appropriate	Responsible and ethical research conduct
5	5.3.29 Evaluate assumptions in the experimental design	Disciplinary approaches
5	5.3.30 Evaluate bias in the experimental design	Disciplinary approaches
5	5.3.31 Evaluate uncertainity in protocols (e.g. ho we measure variables), analytical methods (e.g., assumptions of statistical tests), and interpretations of results	Data analysis approaches
5	5.3.32 Evaluate limitations of methods	Disciplinary approaches
5	5.3.33 Design the research process to include multiple iterations (or repeated experiments)	Disciplinary approaches
5	5.3.34 Use feedback from preliminary results to improve protocols in new experiments	Inferences and Implications
5	5.3.35 Use feedback from results to refine hypotheses and predictions	Inferences and Implications
5	5.4 The ability to conduct an investigation to achieve research goals.	N/A - Heading
5	5.4.1 Record observational data carefully and appropriately	Record keeping and data storage
5	5.4.2 Measure the response of the subjects to the treatment conditions carefully and appropriately	Practical research skills/techniques
5	5.4.3 Monitor study for unexpected outcomes due to technical errors, equipment failure, subject characteristics, and unplanned factors	Practical research skills/techniques
5	5.4.4 Evaluate potential for non-treatment causes for differences or similarities in research outcomes	Interpret results
5	5.4.5 Troubleshoot technical errors	Problem solving
5	5.4.6 Maintain a written or digital laboratory notebook or field journal that provides a record describing how, when, where, and why data were collected	Record keeping and data storage
5	5.4.7 Archive important and sensitive data in an accessible format that is intelligible, secure, and ethical	Record keeping and data storage
5	5.4.8 Record data in an organized and systematic way using appropriate tables, forms, etc.	Record keeping and data storage
5	5.4.9 Enter data with appropriate labels, units of measure, and levels of precision	Record keeping and data storage
5	5.5 The ability to analyze and process data.	N/A - Heading
5	5.5.1 Construct appropriate ways to organize data (e.g., tables, figures)	Visualize data
5	5.5.2 Explore and reduce raw data to discern trend and summarize relationships among variables	Data analysis approaches
5	5.5.3 Idenfity outliers and/or errant data by generating criteria for inclusion or rejection of data	Data analysis approaches

Framework	Element	Theme
5	5.5.4 Display appropriate comparisons (i.e. detect natural groupings)	Data analysis approaches
5	5.5.5 Conduct transformations that facilitate statistical or other analytic tests	Data analysis approaches
5	5.5.6 Conduct computations for summarizing/interpreting findings	Data analysis approaches
5	5.5.7 Analyze clean data using discipline-appropriate methods based on the measurements collected and the experimental questions	Data analysis approaches
5	5.5.8 Choose and conduct statistical tests that are appropriate for the type/nature of data	Data analysis approaches
5	5.5.9 Choose and conduct statistical tests that are aligned with hypotheses and experimental research methods	Data analysis approaches
5	5.5.10 Generate statistics for a sample to summarize and/or describe parameters for a whole population (e.g., mean, median, measures of variance)	Data analysis approaches
5	5.5.11 Appropriately identify a legend, label axes, and select appropriate scale to graph findings	Visualize data
5	5.5.12 Considering the variables intended for comparisons, select an appropriate graphical type for the particular data type (e.g. contingency tables, bar graphs, histograms, scatterplots, etc.)	Visualize data
5	5.5.13 Display finding with a representation that is effective in summarizing trends or major findings, including illustrating contrasts among categorical groups where relevant	Visualize data
5	5.6 The ability to conclude about data with inferences that are limited to the scope inherent in the experimental design.	N/A - Heading
5	5.6.1 Describe trends in numeric an visual representations of data	Interpret results
5	5.6.2 Interpret whether the results suggest a causal mechanism beyond simple correlation	Interpret results
5	5.6.3 Distinguish biologically-meaningful trends from expected natural biological variability	Interpret results
5	5.6.4 Generalize results to an appropriate level (more than single experiment, less than universal)	Inferences and Implications
5	5.6.5 Connect analysis of results with valid claims or conclusion in a logical way	Interpret results
5	5.6.6 Evaluate limitations of the findings and limitations that determine scope of inference (experimental and practical limitations)	Critical evaluation - content knowledge
5	5.6.7 Compare results to other previously reported results and reconcile differences	Inferences and Implications
5	5.6.8 Align conclusion with analyses, hypotheses, reserach question(s), and existing knowledge	Inferences and Implications
5	5.6.9 Determine and articulate whether data support or refute hypotheses and predictions	Interpret results
5	5.6.10 Express uncertainty by discussing limitations of data analysis (sources of error, inaccurate measurement, and sample bias, statistical significance vs. biological relevance)	Data analysis approaches
5	5.6.11 Identify future directions that will make conclusions more certain	Inferences and Implications
5	5.6.12 Understand that scientific knowledge is tentative	Disciplinary content knowledge
5	5.7 The ability to communicate research work in professionally appropriate modes, including visual, written, and oral formats.	N/A - Heading
5	5.7.1 Distill results into clear numeric and/or graphical forms that are aligned with the experimental objective/question/hypothesis	Visualize data
5	5.7.2 Develop a predictive or explanatory model to summarize research findings	Use and test disciplinary theories, frameworks, and models

Framework	Element	Theme
5	5.7.3 Construct scientific communications using standard conventions	Communicating research & research ideas
5	5.7.4 Distinguish typical structure and detail of an oral versus a written presentation	Communicating research & research ideas
5	5.7.5 Tailor structure and content of a presentation to the probable audience (e.d., scientific vs. public)	Communicating research & research ideas
5	5.7.6 Construct a wide range of representations such as tables, graphs, slides, diagrams, animations, and simulations to present main points clearly in written and oral presentations	Visualize data
5	5.7.7 Select the representation that best depicts the data to allow for appropriate inferences	Visualize data
5	5.7.8 Articulate limitations, unanswered questions, and the tentative nature of results (both positive and negative)	Inferences and Implications
5	5.7.9 Contrast results and finding with previously published scientific work	Inferences and Implications
5	5.7.10 Offer alternative hypotheses	Inferences and Implications
5	5.7.11 Construct a justification and counter-justification argument for each alternative, if possible	Inferences and Implications
5	5.7.12 Evaluate, analyze, and explain the significance and implications of the research	Inferences and Implications
5	5.7.13 Revise an existing model based on observations or data	Inferences and Implications
5	5.7.14 Articulate how findings contribute to new knowledge that can drive further inquiry	Inferences and Implications
5	5.7.15 Propose follow up experiments based on inferences from predicted or actual results of experiments	Inferences and Implications
6	6.1 Process of Science	N/A - Heading
6	6.1.1 Scientific Thinking: Explain how science generates knowledge of the natural world	Disciplinary content knowledge
6	6.1.1.1 Explain how scientists use inference and evidence-based reasoning to generate knowledge	Disciplinary content knowledge
6	6.1.1.2 Describe the iterative nature of science and how new evidence can lead to the revision of scientific knowledge	Disciplinary content knowledge
6	6.1.2 Information Literacy: Locate, interpret, and evaluate scientific information	Search disciplinary literature/databases
6	6.1.2.1 Find and evaluate the credibility of a variety of sources of scientific information, including popular science media and scientific journals	Search disciplinary literature/databases
6	6.1.2.2 Interpret, summarize, and evaluate evidence in primary literature	Critical evaluation - content knowledge
6	6.1.2.3 Evaluate claims in scientific papers, popular science media, and other sources using evidence-based reasoning	Critical evaluation - content knowledge
6	6.1.3 Question Formulation: Post testable questions and hypotheses to address gaps in knowledge	Aims, RQs & Hypotheses
6	6.1.3.1 Recognize gaps in our current understanding of a biological system or process and identify what specific information is missing	Inferences and Implications
6	6.1.3.2 Develop research questions based on your own or others' observations	Aims, RQs & Hypotheses
6	6.1.3.3 Formulate testable hypotheses and state their predictions	Aims, RQs & Hypotheses
6	6.1.4 Study Design: Plan, evaluate, and implement scientific investigations	Disciplinary approaches
6	6.1.4.1 Compare the strengths and limitations of various study designs	Disciplinary approaches
6	6.1.4.2 Design controlled experiments, including plans for analyzing the data	Disciplinary approaches
6	6.1.4.3 Execute protocols and accurately record measurements and observations	Practical research skills/techniques
6	6.1.4.4 Identify methodological problems and suggest how to troubleshoot them	Problem solving
6	6.1.4.5 Evaluate and suggest best practices for responsible research conduct (e.g., lab safety, record keeping, proper citation of sources)	Responsible and ethical research conduct

Framework	Element	Theme
6	6.1.5 Data Interpretation and Evaluation: Interpret, evaluate, and draw conclusions from data in order to make evidence-based arguments about the natural world	Interpret results
6	6.1.5.1 Analyze data, summarize resulting patterns, and draw appropriate conclusions	Interpret results
6	6.1.5.2 Describe sources of error and uncertainity in data	Interpret results
6	6.1.5.3 Make evidence-based arguments using your own and others' findings	Inferences and Implications
6	6.1.5.4 Relate conclusions to original hypothesis, consider alternative hypotheses, and suggest future research directions based on findings	Inferences and Implications
6	6.1.6 Doing Research: Apply science process skills to address a research question in a course-based or independent research experience	Practical research skills/techniques
6	6.2 Quantitative Reasoning	N/A - Heading
6	6.2.1 Numercy: Use basic mathamatics (e.g., algebra, probability, unit conversions) in biological contexts	Data analysis approaches
6	6.2.1.1 Perform basic calculations (e.g., percentages, frequencies, rates, means)	Data analysis approaches
6	6.2.1.2 Select and apply appropriate equations (e.g., Hardy-Weinberg, Nernst, Gibbs free energy) to solve problems	Practical research skills/techniques
6	6.2.1.3 Interpret and manipulate mathematical relationships (e.g., scale, ratios, units) to make quantitative comparisons	Data analysis approaches
6	6.2.1.4 Use probability and understanding of biological variability to reason about biological processes and statistical analyses	Inferences and Implications
6	6.2.1.5 Use rouch estimates informed by biological knowledge to check quantitative work	Interpret results
6	6.2.1.6 Describe how quantitative reasoning helps biologists understand the natural world	Disciplinary content knowledge
6	6.2.2 Quantitative and Computational Data Analysis	Data analysis approaches
6	6.2.2.1 Record, organize, and annotate simple data sets	Record keeping and data storage
6	6.2.2.2 Create and interpret informative graphs and other data visualizations	Visualize data
6	6.2.2.3 Select, carry out, and interpret statistical analyses	Data analysis approaches
6	6.2.2.4 Describe how biologists answer research questions using databases, large data sets, and data science tools	Disciplinary approaches
6	6.2.2.5 Interpret the biological meaning of quantitative results	Interpret results
6	6.3 Modeling	N/A - Heading
6	6.3.1 Purpose of Models: Recognize the important roles that scientific models, of many different types (conceptual, mathematical, physical, etc.) play in predicting and communicating biological phenomena	Disciplinary content knowledge
6	6.3.1.1 Describe why biologists use simplified representations (models) when solving problems and communicating ideas	Disciplinary content knowledge
6	6.3.1.2 Given two models of the same biological process or system, compare their strengths, limitations, and assumptions	Use and test disciplinary theories, frameworks, and models
6	6.3.2 Model Application: Make inferences and solve problems using models and simulations	Inferences and Implications
6	6.3.2.1 Summarize relationships and trends that can be inferred from a given model or simulation	Inferences and Implications
6	6.3.2.2 Use models and simulations to make predictions and refine hypotheses	Use and test disciplinary theories, frameworks, and models
6	6.3.3 Modeling: Build and evaluate models of biological systems	Use and test disciplinary theories, frameworks, and models
6	6.3.3.1 Build and revise conceptual models to propose how a biological system or process works	Use and test disciplinary theories, frameworks, and models

Framework	Element	Theme
6	6.3.3.2 Identify important components of a system and describe how they influence each other (e.g., positively or negatively)	Use and test disciplinary theories, frameworks, and models
6	6.3.3.3 Evaluate conceptual, mathematical, or computational models by comparing their predictions with empherical data	Use and test disciplinary theories, frameworks, and models
6	6.4 Interdisciplinary Nature of Science	N/A - Heading
6	6.4.1 Connecting Scientific Knowledge: Integrate concepts across other STEM disciplines (e.g., chemistry, physics) and multiple fields of biology (e.g., cell biology, ecology)	Disciplinary content knowledge
6	6.4.1.1 Given a biological problem, identify relevant concepts from other STEM disciplines or fields of biology	Disciplinary content knowledge
6	6.4.1.2 Build models or explanations of simple biological processes that include concepts from other STEM disciplines or multiple fields of biology	Use and test disciplinary theories, frameworks, and models
6	6.4.2 Interdisciplinary Problem Solving: Consider interdisciplinary solutions to real-world problems	Problem solving
6	6.4.2.1 Describe examples of real-world problems that are too complex to be solved by applying biological approaches alone	Disciplinary content knowledge
6	6.4.2.2 Suggest how collaborators in STEM and non-STEM disciplines could contribute to solutions of real-world problems	Translating research to practice
6	6.4.2.3 Be able to explain biological concepts, data, and methods, including their limitations, using language understandable by collaborators in other disciplines	Communicating research & research ideas
6	6.5 Communication and Collaboration	N/A - Heading
6	6.5.1 Communication: Share ideas, data, and findings with others clearly and accurately	Communicating research & research ideas
6	6.5.1.1 Use appropriate language and style to communicate science effectively to targeted audiences (e.g., general public, biology experts, collaborators in other disciplines)	Communicating research & research ideas
6	6.5.1.2 Use a variety of modes to communicate science (e.g., oral, written, visual)	Communicating research & research ideas
6	6.5.2 Collaboration: Work productively in teams with people who have diveres backgrounds, skill sets, and perspectives	Collaboration
6	6.5.2.1 Work with teammates to establish and periodically update group plans and expectations (e.g., team goals, project timeline, rules for group interactions, individual and collaborative tasks)	Team management
6	6.5.2.2 Elicit, listen to, and incorporate ideas from teammates with different perspectives and backgrounds	Inclusive research practice
6	6.5.2.3 Work effectively with teammates to complete projects	Collaboration
6	6.5.3 Collegial Review: Provide and respond to constructive feedback in order to improve individual and team work	Give feedback on research
6	6.5.3.1 Evaluate feedback from others and revise work or behavior appropriately	Receive research feedback
6	6.5.3.2 Critique others' work and ideas constructively and respectfully	Give feedback on research
6	6.5.4 Metacognition: Reflect on your own learning, performance, and achievements	Self Reflective
6	6.5.4.1 Evaluate your own understanding and skill level	Self Reflective
6	6.5.4.2 Assess personal progress and contributions to your team and generate a plan to change your behavior as needed	Self Reflective
6	6.6 Science and Society	N/A - Heading
6	6.6.1 Ethics: Demonstrate the ability to critically analyze ethical issues in the conduct of science	Responsible and ethical research conduct
6	6.6.1.1 Identify and evaluate ethical considerations (e.g., use of animal or human subjects, conflicts of interest, confirmation bias) in a given research study	Responsible and ethical research conduct

Framework	Element	Theme
6	6.6.1.2 Critique how ethical controversies in biological research have been and can continue to be addressed by the scientific community	Responsible and ethical research conduct
6	6.6.2 Societal Influences: Consider the potential impacts of outside influences (historical, cultural, political, technological) on how science is practiced	Cultural context of research
6	6.6.2.1 Describe examples of how scientists' backgrounds and biases can influence science and how science is enhanced through diversity	Cultural context of research
6	6.6.2.2 Identify and describe how systemic factors (e.g., socioeconomic, political) affect how and by whom science is conducted	Cultural context of research
6	6.6.3 Science's Impact on Society: Apply scientific reasoning in daily life and recognize the impacts of science on a local and global scale	Translating research to practice
6	6.6.3.1 Apply evidence-based reasoning and biological knowledge in daily life (e.g., consuming popular media, deciding how to vote)	Translating research to practice
6	6.6.3.2 Use examples to describe the relevance of science in everyday experiences	Outreach
6	6.6.3.3 Identify and describe the broader societal impacts of biological research on different stakeholders	Outreach
6	6.6.3.4 Describe the roles scientists have in facilitating public understanding of science	Outreach
7	7.1 Technical Knowledge	N/A - Heading
7	7.1.1 Chemistry Knowledge: both basic and advanced chemistry content knowledge	Disciplinary content knowledge
7	7.1.2 Experimental Skill: executing experiment, using instruments, collecting data	Practical research skills/techniques
7	7.1.3 Data Analysis: analyzing data, interpreting data, quantitative analysis	Data analysis approaches
7	7.1.4 Literature Evaluation: literature searching, review and analysis	Search disciplinary literature/databases
7	7.1.5 Relevant Working Experience	Practical research skills/techniques
7	7.1.6 Computer Literacy: how to use Excel properly, coding, programming, etc.	Practical research skills/techniques
7	7.2 Communication Skill	N/A - Heading
7	7.2.1 Oral Communication Skill: via presentation or general talking without presentation	Communicating research & research ideas
7	7.2.2 Written Communication Skill: via written text, journals, books, grants, or other written materials	Communicating research & research ideas
7	7.3 Management Skill	N/A - Heading
7	7.3.1 Laboratory Management	Team management
7	7.3.2 Teaching Management	Omit
7	7.3.3 Project Management	Project management
7	7.4 Teamwork/Collaboration Skills	Collaboration
7	7.4.1 Interpersonal Understanding	Effective interpersonal research relationships
7	7.4.2 Sharing Learnings and Experiences: sharing what have been learned from the previous project and take to the next one	Inferences and Implications
7	7.4.3 Whom to Work With: classifying different kinds of colalborators	Collaboration
7	7.4.4 Having a Scope: knowing the team goal, rules	Collaboration
7	7.4.5 Everyone Knows the Progress: allowing people who have done the job to see the result and conclusion	Communicating research & research ideas
7	7.4.6 Giving feedback: providing feedback to collaborators	Give feedback on research
7	7.5 Planing and Organizational Skill	N/A - Heading
7	7.5.1 Time Management Skill	Time Management
7	7.5.2 Organizational Skill: the ability to organize oneself and others	Project management
7	7.6 Teaching Skill	Omit
7	7.6.1 Course Preparation: including preparing lectures, homeworks, exams, labs, etc.	Omit

Framework	Element	Theme
7	7.6.2 Teaching Strategy: teaching strategies that can help students learn better	Omit
7	7.6.3 Learning From Other Sources: internet materials, other universities	Search disciplinary literature/databases
7	7.6.4 Explaining in Simple Terms: breaking concepts down	Communicating research & research ideas
7	7.6.5 Interpersonal Skill: listening, approachable, awareness of others' feelings, caring	Effective interpersonal research relationships
7	7.6.6 Being Responsive: asking and answering questions, and responding quickly	Effective interpersonal research relationships
7	7.7 Personal Value/Attributes: combination of characteristics or qualities that form an individual's distinctive character	N/A - Heading
7.8	7.8 Problem Solving Skill	Problem solving
7	7.8.1 Problem Solving in Class: textbook problems	Omit
7	7.8.2 Trouble shooting: real world problems	Problem solving
7	7.9 Personal Growth/Development	N/A - Heading
7	7.9.1 Willing to Learn New Things	Curiosity
7	7.9.2 Taking Classes/Trainings to Learn New Things	Disciplinary content knowledge
7	7.9.3 Continuous Improvement Through Practice	Practical research skills/techniques
7	7.1O Critical Thinking	N/A - Heading
7	7.10.1 Creativity	Creative thinking
7	7.10.2 Connecting Small Pieces	Inferences and Implications
7	7.10.3 Awareness of External Influences	Inferences and Implications
7	7.11 Organizational Awareness: talent development	N/A - Heading
7	7.11.1 Business Mindedness: knowing cost, capital and how profit is made	Budget Management
7	7.11.2 Understading Rules and Regulations	Responsible and ethical research conduct
7	7.12 Networking Skill	N/A - Heading
8	8.1 Communication	N/A - Heading
8	8.1.1 Uses and understands professional and discipline-specific language	Communicating research & research ideas
8	8.1.2 Expresses ideas orally in an organized, clear, and concise manner	Communicating research & research ideas
8	8.1.3 Writes clearly and concisely using correct grammar, spelling, syntax, and sentence structure	Communicating research & research ideas
8	8.2 Creativity	N/A - Heading
8	8.2.1 Displays insight about the topic being investigated	Disciplinary content knowledge
8	8.2.2 Shows ability to approach problems from different perspectives	Disciplinary approaches
8	8.2.3 Uses information in ways that demonstrate intellectual resourcefulness	Disciplinary content knowledge
8	8.2.4 Effectively connects multiple ideas/approaches	Inferences and Implications
8	8.3 Autonomy	N/A - Heading
8	8.3.1 Demonstrates an ability to work independently and identify when guidance is needed	Independence
8	8.3.2 Accepts constructive criticism and uses feedback effectively	Receive research feedback
8	8.3.3 Uses time well to ensure work gets accomplished	Project management
8	8.3.4 Sets and meets project deadlines	Project management
8	8.4 Ability to Deal with Obstacles	N/A - Heading
8	8.4.1 Is not discouraged by unforeseen problems and perseveres when challenges or setbacks are encountered	Grit
8	8.4.2 Shows flexibility and a willingness to take risks and try again	Grit
8	8.4.3 Trouble-shoots problems and searches for ways to do things more effectively	Problem solving
8	8.5 Intellectual Development	N/A - Heading
8	8.5.1 Recognizes that problems are often more complicated than they first appear	Disciplinary content knowledge

Framework	Element	Theme
8	8.5.2 Approaches problems with an understanding that there can be more than one right explanation or even none at all	Disciplinary content knowledge
8	8.5.3 Displays accurate insight into the limits of his/her own knowledge and an appreciation for what is not known	Self Reflective
8	8.6 Critical Thinking and Problem Solving	Critical thinking
8	8.6.1 Maintains a posture of open-minded skepticism when considering potential solutions to problems; Challenges established thinking when appropriate	Problem solving
8	8.6.2 Looks for the root causes of problems and develops or recognizes the most appropriate corrective actions	Problem solving
8	8.6.3 Recognizes flaws, assumptions and missing elements in arguments	Critical evaluation - content knowledge
8	8.7 Practice and Process of Inquiry	N/A - Heading
8	8.7.1 Demonstrates ability to formulate questions and hypotheses within the discipline	Aims, RQs & Hypotheses
8	8.7.2 Demonstrates ability to properly identify and/or generate reliable data	Disciplinary approaches
8	8.7.3 Shows understanding of how knowledge is generated, validated and communicated within the discipline	Disciplinary approaches
8	8.8 Nature of Disciplinary Knowledge	N/A - Heading
8	8.8.1 Shows understanding of the way practitioners think within the discipline and view the world around them	Disciplinary approaches
8	8.8.2 Shows understanding of the criteria for determining what is valued as a contribution in the discipline	Critical evaluation - content knowledge
8	8.8.3 Shows awareness of important contributions in the discipline and who was responsible for those contributions	Disciplinary content knowledge
8	8.8.4 Reads and applies information obtained from professional journals and other sources	Inferences and Implications
8	8.8.5 Is aware of professional societies in the discipline	Professionalism
8	8.9 Content Knowledge and Methods	N/A - Heading
8	8.9.1 Displays knowledge of key facts and concepts	Disciplinary content knowledge
8	8.9.2 Displays a grasp of relevant research methods and is clear about how these methods apply to the research project	Disciplinary approaches
8	8.9.3 Demonstrates an appropriate mastery of skills needed to conduct the project	Practical research skills/techniques
8	8.10 Ethical Conduct	N/A - Heading
8	8.10.1 Understands that altering or fabricating data is highly unethical.	Responsible and ethical research conduct
8	8.10.2 Realizes that distorting or misstating research findings is unethical and may harm others who rely on your professional integrity	Responsible and ethical research conduct
8	8.10.3 Recognizes the imperative of giving credit to sources used in research and to those who may have provided helpful advice or assistance	Responsible and ethical research conduct
8	8.11 Career Goals	N/A - Heading
8	8.11.1 Is clear about academic and/or professional/work plans	Career development
8	8.11.2 Is aware of how research skills relate to academic and/or professional/work plans	Career development
8	8.12 Teamwork/Collaboration	N/A - Heading
8	8.12.1 Behaves with a high level of collegiality and treats others with respect	Professionalism
8	8.12.2 Shows ability to work effectively in a team	Collaboration
8	8.12.3 Willingly shares knowledge among team members and amongst other teams (as applicable)	Collaboration
9	9.1.1 Develop Effective Interpersonal Communication Skills	Effective interpersonal research relationships

Framework	Element	Theme
9	9.1.2 Develop Disciplinary Knowledge	Disciplinary content knowledge
9	9.1.3 Develop Research Communication Skills	Communicating research & research ideas
9	9.1.4 Develop Logical/Critical Thinking Skills	Critical thinking
9	9.1.5 Develop an Understanding of the Research Environment	Professionalism
9	9.2.1 Develop Ability to Design a Research Project	Disciplinary approaches
9	9.2.2 Develop Ability to Conduct a Research Project	Practical research skills/techniques
9	9.3.1 Develop Responsible and Ethical Research Practices	Responsible and ethical research conduct
9	9.4.1 Develop Identitiy as a Researcher	Identity
9	9.5.1 Develop Confidence as a Researcher	Confidence
9	9.5.2 Develop Independence as a Researcher	Independence
9	9.6.1 Develop Skills to Deal with Personal Differences in the Research Environment	Effective interpersonal research relationships
9	9.6.2 Advance Equity and Inclusion in the Research Environment	Inclusive research practice
9	9.7.1 Explore and Pursue a Research Career	Career development
9	9.7.2 Develop Confidence in Pursuing a Research Career	Career development
9	9.7.3 Translate Research Skills Across Career Pathways	Career development
9	9.8 Develop Research Leadership and Management Skills	Leadership
9	9.9 Develop Research Mentoring and Teaching Skills	Mentoring
10	10.1 Knowledge and Intellectual Abilities	Disciplinary content knowledge
10	10.1.1 Knowledge base	Disciplinary content knowledge
10	10.1.2 Cognitive abilities	Critical thinking
10	10.1.3 Creativity	Creative thinking
10	10.2 Personal Effectiveness	Professionalism
10	10.2.1 Personal qualities	Omit
10	10.2.2 Self-management	Self-Regulation in Research
10	10.2.3 Professional and career development	Career development
10	10.3 Research Governance and Organisation	Project management
10	10.3.1 Professional conduct	Professionalism
10	10.3.2 Research management	Project management
10	10.3.3 Finance, funding, and resources	Budget Management
10	10.4 Engagement, Influence and Impact	Leadership
10	10.4.1 Working with others	Collaboration
10	10.4.2 Communication and dissemination	Communicating research & research ideas
10	10.4.3 Engagement and impact	Outreach
11	11.1.1 Health data analytics: Development and evaluation of data visualizations and dashboards	Visualize data
11	11.1.2 Health data analytics: Applying data mining/machine learning models	Practical research skills/techniques
11	11.1.3 Health data analytics: Applying statistical methods and models	Data analysis approaches
11	11.1.4 Health data analytics: Development of business intelligence systems	Disciplinary approaches
11	11.2.1 Research competencies: Designing and conducting research in health information and digital health	Aims, RQs & Hypotheses
11	11.2.2 Research competencies: Publication of research results	Communicating research & research ideas
11	11.2.3 Research competencies: Development of research proposals and applying for grants	Communicating research & research ideas
11	11.2.4 Research competencies: Future studies for health information technology and digital health	Aims, RQs & Hypotheses
12	12.01 literature review: A1. Enquiring mind/curiosity	Curiosity

Framework	Element	Theme
12	12.02 literature review: A2. Exploring general information sources to increase familiarity with topic	Search disciplinary literature/databases
12	12.03 literature review: A3. Recognising gaps in the literature	Critical evaluation - content knowledge
12	12.04 literature review: A4. Formulating a structured answerable question using PICO format	Aims, RQs & Hypotheses
12	12.05 literature review: A5. Identifying key concepts and terms that describe information need	Disciplinary content knowledge
12	12.06 literature review: A6. Successfully searching for and locating relevant literature	Search disciplinary literature/databases
12	12.07 literature review: A7. Searching for literature when off campus	Search disciplinary literature/databases
12	12.08 literature review: A8. Knowledge of evidence sources and types, including their strengths and weaknesses	Disciplinary content knowledge
12	12.09 literature review: A9. Choosing an appropriate database	Search disciplinary literature/databases
12	12.10 literature review: A10. Constructing a systematic and comprehensive search strategy that reflects the purpose of the study	Search disciplinary literature/databases
12	12.11 literature review: A11. Applying a search strategy: narrowing a search, use of keywords, Boolean, truncation, search filters and MeSH	Search disciplinary literature/databases
12	12.12 literature review: A12. Managing references/software	Search disciplinary literature/databases
12	12.13 literature review: A13. Strategies to obtain full texts of relevant articles	Search disciplinary literature/databases
12	12.14 literature review: A14. Reading and understanding scientific articles, including research terminology	Disciplinary content knowledge
12	12.15 research methodology/ processes: B15. Knowledge of research design (differentiating and defining)	Disciplinary approaches
12	12.16 research methodology/ processes: B16. Knowing strength and weaknesses of each study design	Disciplinary approaches
12	12.17 research methodology/ processes: B17. Understanding hierarchy of levels of evidence	Disciplinary content knowledge
12	12.18 research methodology/ processes: B18. Knowing the best type of design to answer question (matching)	Disciplinary approaches
12	12.19 research methodology/ processes: B19. Critical appraisal of different study designs using CAT	Disciplinary approaches
12	12.20 research methodology/ processes: B20. Basic knowledge of biostatistics	Data analysis approaches
12	12.21 research methodology/ processes: B21. Evaluating statistical tests and principles	Disciplinary approaches
12	12.22 research methodology/ processes: B22. Reporting statistics	Communicating research & research ideas
12	12.23 research methodology/ processes: B23. Sample size determination	Disciplinary approaches
12	12.24 research methodology/ processes: B24. Data collection skills	Practical research skills/techniques
12	12.25 research methodology/ processes: B25. Data analysis skills	Data analysis approaches
12	12.26 research methodology/ processes: B26. Using data analysis techniques consistent with research question/hypotheses	Data analysis approaches
12	12.27 research methodology/ processes: B27. Using statistical software package	Practical research skills/techniques
12	12.28 research methodology/ processes: B28. Interpretation/synthesis of findings	Interpret results
12	12.29 research methodology/ processes: B29. Implications for future research and practice for each discipline	Inferences and Implications
12	12.30 research methodology/ processes: B30. Interpreting the certainty in evidence and strength of recommendation in healthcare	Inferences and Implications
12	12.31 other research processes: B31. Writing a grant application	Communicating research & research ideas
12	12.32 other research processes: B32. Identifying graduate funding	Budget Management

Framework	Element	Theme
12	12.33 other research processes: B33. Identifying mentors	Mentoring
12	12.34 other research processes: B34. Knowing the authorship process	Authorship
12	12.35 other research processes: C35. Scientific writing: understanding rules for citations, referencing, writing style, formatting, plagiarism	Communicating research & research ideas
12	12.36 other research processes: C36. Publishing research	Communicating research & research ideas
12	12.37 other research processes: C37. Oral presentation	Communicating research & research ideas
12	12.38 other research processes: D38. Communication skills	Communicating research & research ideas
12	12.39 other research processes: D39. Independent and critical thinking skills	Critical thinking
12	12.40 other research processes: D40. Problem-solving skills	Problem solving
12	12.41 other research processes: D41. Team-working skills/working in groups	Collaboration
12	12.42 other research processes: D42. Reflective skills	Self Reflective
12	12.43 other research processes: E43. Addressing ethical and legal issues	Responsible and ethical research conduct
12	12.44 other research processes: F44. Evidence-based practice essential to clinical work	Translating research to practice
12	12.45 other research processes: F45. Learning by doing	Practical research skills/techniques
13	13.01 knowledge-based competencies: Advanced research methods, RCTs, prospective and qualitative methods	Disciplinary approaches
13	13.02 knowledge-based competencies: Advanced statistical analyses (e.g., mixed linear modeling, group-based trajectory analysis)	Data analysis approaches
13	13.03 knowledge-based competencies: New methods of measurement, such as electronic monitoring, cell phone applications, etc.	Disciplinary approaches
13	13.04 knowledge-based competencies: Manuscripts for different professional audiences and different article formats	Communicating research & research ideas
13	13.05 knowledge-based competencies: Types of research grants, content, and formats of grants	Communicating research & research ideas
13	13.06 knowledge-based competencies: Specialized medical populations: etiology, medical treatment, and natural history of medical conditions	Disciplinary content knowledge
13	13.07 knowledge-based competencies: Focus and types of interprofessional research teams	Collaboration
13	13.08 knowledge-based competencies: Methods of research mentorship/supervision	Mentoring
13	13.09 knowledge-based competencies: Methods to integrate research and practice	Translating research to practice
13	13.10 knowledge-based competencies: Methods of responsible conduct of research in medical populations	Responsible and ethical research conduct
13	13.11 applied competencies: Implement prospective, qualitative studies, and RCTs	Practical research skills/techniques
13	13.12 applied competencies: Select, conduct, and interpret advanced data analysis	Data analysis approaches
13	13.13 applied competencies: Select and apply measures in study design and data analysis	Disciplinary approaches
13	13.14 applied competencies: Prepare first-authored manuscripts in different formats (e.g. meta-analyses, case studies) and for different audiences	Communicating research & research ideas
13	13.15 applied competencies: Sections of grants with mentor and independent grant proposals	Communicating research & research ideas

Framework	Element	Theme
13	13.16 applied competencies: Conduct research with medical populations	Practical research skills/techniques
13	13.17 applied competencies: Communicate and collaborate effectively within interprofessional teams	Effective interpersonal research relationships
13	13.18 applied competencies: Mentoring of student volunteers and research assistants in research activities	Mentoring
13	13.19 applied competencies: Providing clinical services to complex populations; delivering clinical interventions in research protocols, implementing research in clinical care	Practical research skills/techniques
13	13.20 applied competencies: Understand and recognize challenging ethical issues in research with medical populations	Responsible and ethical research conduct
13	13.21 benchmarks: Develop, participate in, and complete studies with various methods	Disciplinary approaches
13	13.22 benchmarks: Complete and summarize data using data analytic approaches	Data analysis approaches
13	13.23 benchmarks: Complete analysis and interpretation of data from multiple measures	Data analysis approaches
13	13.24 benchmarks: Submit and revise a range of first-authored manuscripts in different formats for different audiences	Communicating research & research ideas
13	13.25 benchmarks: Submit and revise grant proposals with mentor and independently	Communicating research & research ideas
13	13.26 benchmarks: Initiate and complete research projects with a range of medical populations	Practical research skills/techniques
13	13.27 benchmarks: Participate and lead activities of interprofessional teams	Collaboration
13	13.28 benchmarks: Initiate and complete mentorship activities with students, volunteers, and research associates	Mentoring
13	13.29 benchmarks: Complete assessments and interventions with clinical cases and patients in research protocols	Practical research skills/techniques
13	13.30 benchmarks: Complete ethics training; complete IRB proposals; mange ethical issues in research studies	Responsible and ethical research conduct
14	14.01 Students embark on inquiry and so determines a need for knowledge/understanding	Aims, RQs & Hypotheses
14	14.02 Students find/generate needed information/data using appropriate methodology	Practical research skills/techniques
14	14.03 Students critically evaluate information/data and the process to find/generate this informational data	Critical evaluation - content knowledge
14	14.04 Students organize information collected/generated	Record keeping and data storage
14	14.05 Students synthesize and analyze new knowledge	Inferences and Implications
14	14.06 Students communicate knowledge and understanding and the processes used to generate them.	Communicating research & research ideas
15	15.01 The ability to design sampling plan	Disciplinary approaches
15	15.02 The ability to define the data required for research	Disciplinary approaches

Framework	Element	Theme
15	15.03 The ability to choose a sample that can represent the population	Disciplinary approaches
15	15.04 The ability to determine the suitability of the people/associations to be included in research	Disciplinary approaches
15	15.05 The ability to define population	Disciplinary approaches
15	15.06 The ability to define dependent variables of the research	Disciplinary approaches
15	15.07 The ability to define independent variables of the research	Disciplinary approaches
15	15.08 The ability to choose effective measurement tools for research	Disciplinary approaches
15	15.09 The ability to process/code the collected data properly	Data analysis approaches
15	15.10 The ability to choose a reliability method suitable for the structure of data collection tool	Disciplinary approaches
15	15.11 The ability to explain the aim of the study in accordance with the research problem	Communicating research & research ideas
15	15.12 The ability to collect the necessary data in a systematic and planned manner	Practical research skills/techniques
15	15.13 The ability to use robust and consistent methods in order to conduct data collection activities in an ethical manner	Responsible and ethical research conduct
15	15.14 The ability to choose a research model suitable for the problem	Use and test disciplinary theories, frameworks, and models
15	15.15 The ability to interpret the analysed data	Interpret results
15	15.16 The ability to choose a validity method suitable for the structure of data collection tool	Disciplinary approaches
15	15.17 The ability to choose a research design in compliance with the theoretical/conceptual framework	Disciplinary approaches
15	15.18 The ability to construct the theoretical/conceptual framework of the research in a valid manner	Use and test disciplinary theories, frameworks, and models
15	15.19 The ability to use a research design in compliance with the statement of aim	Disciplinary approaches
15	15.20 The ability to form a correct/valid hypothesis	Aims, RQs & Hypotheses
15	15.21 The ability to assess the theoretical/conceptual frameworks for the feasibility of the study	Use and test disciplinary theories, frameworks, and models
15	15.22 The ability to infer the type of a research article from its title	Critical evaluation - content knowledge
15	15.23 The ability to analyse data	Data analysis approaches
15	15.24 The ability to assess the strengths/weaknesses of research methods	Disciplinary approaches
15	15.25 The ability to use appropriate data analysis techniques which are consistent with the aim and design of the study	Data analysis approaches
15	15.26 The ability to use a research design in compliance with the problem statement	Disciplinary approaches
15	15.27 The ability to interpret the psychometric features of a measurement tool	Disciplinary approaches
15	15.28 Compliance with scientific honesty standards	Responsible and ethical research conduct
15	15.29 Being open-minded	Curiosity
15	15.30 Compliance with ethical behaviour standards	Responsible and ethical research conduct
15	15.31 The ability to form effective purpose-oriented relationships with the relevant people	Effective interpersonal research relationships
15	15.32 The ability to use written communication effectively in order to express his/her ideas	Communicating research & research ideas
15	15.33 Being attentive in their studies	Self-Regulation in Research
15	15.34 The ability to use oral communication effectively in order to express his/her ideas	Communicating research & research ideas
15	15.35 The ability to consider the way s/he works in order to improve his/her performance/study	Self Reflective
15	15.36 The ability to follow the work in progress until obtaining results	Grit

Framework	Element	Theme
15	15.37 Making efforts required to complete the undertaken work	Project management
15	15.38 Working in a certain manner systematically / in a planned manner	Disciplinary content knowledge
15	15.39 Guiding someone through his/her research in case of having relevant experience	Mentoring
15	15.40 The ability to find alternative in cases of crisis	Problem solving
15	15.41 The ability to change his/her personal opinions in cases of objective findings	Critical evaluation - content knowledge
15	15.42 The ability to stay patient when finalising works	Grit
15	15.43 Being happy about providing support for research	General research beliefs
15	15.44 Being cautious	Safety
15	15.45 The ability to remain calm in the event of a crisis	Professionalism
15	15.46 Feeling free to use his/her own initiative when necessary	Independence
15	15.47 The ability to write a grant proposal for the research	Communicating research & research ideas
15	15.48 The ability to perceive threats to internal validity regarding research	Disciplinary approaches
15	15.49 The ability to take measures regarding threats to research beforehand	Disciplinary approaches
15	15.50 The ability to calculate interest regarding research	Leadership
15	15.51 The ability to perceive threats of external validity regarding research	Disciplinary approaches
15	15.52 The ability to predict opportunities regarding research	Leadership
15	15.53 The ability to conduct risk analyses regarding research	Disciplinary approaches
15	15.54 The ability to calculate costs regarding research	Budget Management
15	15.55 The ability to communicate orally using at least one foreign language	Communicating research & research ideas
15	15.56 The ability to communicate in writing using at least one foreign language	Communicating research & research ideas
15	15.57 The ability to follow scientific publications in at least one foreign language	Disciplinary content knowledge
16	16.01 Criteria for selecting & judging research questions	Aims, RQs & Hypotheses
16	16.02 Steps of the process of developing a research question	Aims, RQs & Hypotheses
16	16.03 Estimation of the effort required to perform specific research methods	Disciplinary approaches
16	16.04 Assessment of the feasibility of a specific research question	Disciplinary approaches
16	16.05 differences between methodologica perspectives on research objects	Disciplinary approaches
16	16.06 basic methodological terms	Disciplinary approaches
16	16.07 steps and reosurces of research planning	Project management
16	16.08 common difficulties in accessing a research field	Disciplinary content knowledge
16	16.09 steps, rules and boundaries of data collection methods	Disciplinary approaches
16	16.10 characteristics of good research instruments	Disciplinary approaches
16	16.11 implications of methodological concepts for reserach planning	Disciplinary approaches
16	16.12 fit between methodology and methods of data collection/analysis	Disciplinary approaches
16	16.13 importance of evaluating one's own research process	Self Reflective
16	16.14 comparison of reserach objectives and the status quo of one's results	Inferences and Implications
16	16.15 steps of data analysis methods	Data analysis approaches
16	16.16 interpretation of data and research results	Interpret results
16	16.17 basic methodological concepts for data analysis	Data analysis approaches
16	16.18 methodological concepts on the validity and range of research results.	Disciplinary approaches
17	17.01 Understand methods of inquiry that lead to scientific knowledge	Disciplinary approaches
17	17.02 Understand how to search for information	Search disciplinary literature/databases
17	17.03 Understand research design	Disciplinary approaches
17	17.04 Understand research techniques/ instrumentation	Practical research skills/techniques
17	17.05 Understand how research design may influence scientific finding	Disciplinary approaches
17	17.06 Troubleshoot technical issues	Problem solving
17	17.07 Interpret, represent, and analyze quantitative scientific data	Data analysis approaches

Framework	Element	Theme
17	17.08 Represent data in a visual form	Visualize data
17	17.09 Interpret visual representations of data	Interpret results
17	17.10 Understand basic statistics	Data analysis approaches
17	17.11 Evaluating scientific information	Critical evaluation - content knowledge
17	17.12 Evaluate evidence and critique experimental designs	Critical evaluation - content knowledge
17	17.13 Identify additional information needed to evaluate a hypothesis/interpretation	Critical evaluation - content knowledge
17	17.14 Provide alternative explanations for results that may have many causes	Inferences and Implications
18	18.1. Identify and utilize relevant previous work that supports the research.	Search disciplinary literature/databases
18	18.2. Articulate a timely and important research question or creative objective.	Aims, RQs & Hypotheses
18	18.3. Identify and utilize appropriate methodologies to address the research question or creative objective	Disciplinary approaches
18	18.4. Present the research effectively in a conference setting and a written publication	Communicating research & research ideas
18	18.5. Meet the relevant field's standards for the responsible conduct of research and effectively navigate challenges that arise in the research process.	Responsible and ethical research conduct
18	18.6. Work collaboratively with other researchers, demonstrating effective communication and problem-solving skills.	Collaboration
18	18.7. Reflect constructively on the research experience, identifying what was learned, personal strengths and opportunities for growth, and how the experience informs future educational and career goals	Self Reflective
19	19.01 Produce reflective theses	Communicating research & research ideas
19	19.02 Autonomous researchers	Independence
19	19.03 Knowledge producers and consumers	Omit
19	19.04 Leaders and managers	Leadership
20	20.01 Working as a researcher: Building supportive relationships	Effective interpersonal research relationships
20	20.02 Working as a researcher: developing research and study skills	Practical research skills/techniques
20	20.03 Working as a researcher: blending theory and practice	Translating research to practice
20	20.04 Developing ways of thinking: Building your resilience	Grit
20	20.05 Developing ways of thinking: Developing your identity	Identity
20	20.06 Developing ways of thinking: Reflecting on theory and practice	Translating research to practice
20	20.07 Moving on with your research: Disseminating your research	Communicating research & research ideas
20	20.08 Moving on with your research: Making a difference	Translating research to practice
20	20.09 Moving on with your research: Engaging with new opportunities	Inferences and Implications
21	21.01 Leadership: The ability to work effectively with stakeholders, manage assets and team members, provide professional development opportunities, communicate and share knowledge effectively, act as an initiator, and create a shared vision for team members	Leadership
21	21.02 Agents of change: The ability to enable change through creating conducive environments, advocate for health, mediate through partnerships and collaborate to improve health outcomes and maximize effectiveness	Translating research to practice
21	21.03 Knowledge and Knowledge translation: Knowledge should be composed of ethical values, principles and values, and evidence-based practice. Knowledge should be formed through credible education, opportunities to apply knowledge and professional development	Disciplinary content knowledge

Framework	Element	Theme
21	21.04 Communication: The ability to effectively communicate to professional and lay audiences through written, verbal, and nonverbal communication; implement new technologies; and develop interprofessional communication and teamwork skills	Communicating research & research ideas
21	21.05 Research, policy & practice: The ability to apply analytical skills and use evidence-based practices to implement or change existing policies and practices	Translating research to practice
21	21.06 Programming & evaluation: The ability to organize and evaluate programs and services through needs assessments, planning, evaluation, implementation, and administration skills	Project management
22	22.01 Mentorship	Mentoring
22	22.02 Community Engagement	Outreach
22	22.03 Inclusivity & Diversity	Inclusive research practice
22	22.04 Networking	Effective interpersonal research relationships
22	22.05 Leadership skills	Leadership
22	22.06 Interpersonal Skills	Effective interpersonal research relationships
22	22.07 Communication Skills	Communicating research & research ideas
22	22.08 Career Planning	Career development
22	22.09 Teaching & Learning	Omit
22	22.10 Critical Thinking	Critical thinking
22	22.11 Academic Service	Professionalism
22	22.12 Academic Writing	Communicating research & research ideas
22	22.13 Time management	Time Management
22	22.14 Work-Life Balance	Wellness
22	22.15 Health & Wellness	Wellness
22	22.16 Project Management	Project management
23	23.01 Basic steps of nursing research	Practical research skills/techniques
23	23.02 Ways to formulate research questions	Aims, RQs & Hypotheses
23	23.03 Statements of research questions	Aims, RQs & Hypotheses
23	23.04 Databases for document retrieval	Search disciplinary literature/databases
23	23.05 Types of databases for document retrieval	Search disciplinary literature/databases
23	23.06 Ways and methods of document retrieval	Disciplinary approaches
23	23.07 Ethical requirements for research	Responsible and ethical research conduct
23	23.08 Types of research design	Disciplinary approaches
23	23.09 Components of research design	Disciplinary approaches
23	23.10 Characteristics of different research designs	Disciplinary approaches
23	23.11 Concepts about population and sample	Disciplinary approaches
23	23.12 Sampling methods	Disciplinary approaches
23	23.13 Ways to estimate sample size	Disciplinary approaches
23	23.14 Data collection methods	Disciplinary approaches
23	23.15 Factors that affect research quality	Disciplinary approaches
23	23.16 Methods to improve research quality	Disciplinary approaches
23	23.17 Ways to measure instrument performance	Practical research skills/techniques
23	23.18 Types of research data	Disciplinary approaches
23	23.19 Methods of statistical analysis	Data analysis approaches

Framework	Element	Theme
23	23.20 Formats of research articles	Disciplinary content knowledge
23	23.21 Ways to write research articles	Communicating research & research ideas
23	23.22 Research article appraisal	Critical evaluation - content knowledge
23	23.23 Concept of evidence-based nursing	Translating research to practice
23	23.24 Steps of evidence-based nursing	Disciplinary approaches
24	24.01a Explain the role of computation and data mining in addressing hypothesis-driven and hypothesisgenerating questions within the life sciences. Life sciences students should have a clear understanding of the role computing and data mining play in modern biology.	Disciplinary approaches
24	24.01b Given a traditional hypothesis-driven research question, students should have ideas about what types of data and software exist that could help them answer the question quickly and efficiently.	Disciplinary approaches
24	24.01c They should also appreciate that mining large datasets can generate novel hypotheses to be tested in the lab or field.	Disciplinary approaches
24	24.02 Summarize key computational concepts, such as algorithms and relational databases, and their applications in the life sciences. To make use of sophisticated software and database tools, students should have a basic understanding of the principles upon which these tools are based and should be exposed to how these tools work	Disciplinary content knowledge
24	24.03 3. Apply statistical concepts used in bioinformatics. In addition to the basic statistics found in many biology curricula, modern life scientists should have an understanding of the statistics of large datasets and multiple comparisons	Data analysis approaches
24	24.04 Use bioinformatics tools to examine complex biological problems in evolution, information flow, and other important areas of biology. This competency is written broadly so as to encompass a variety of problems that can be addressed using bioinformatics tools, such as understanding the evolutionary underpinnings of sequence comparison and homology detection; distinguishing between genomic sequences, RNA sequences, and protein sequences; and interpreting phylogenetic trees. "Complex" biological problems require that students should be able to work through a problem with multiple steps, not just perform isolated tasks.	Practical research skills/techniques
24	24.05 5. Find, retrieve, and organize various types of biological data. Given the numerous and varied datasets currently being generated from all of the 'omics fields, students should develop the facility to identify appropriate data repositories, navigate and retrieve data from these repositories, and organize data relevant to their area of study in flat files or small local stand-alone databases	Practical research skills/techniques
24	24.06 a . Explore and/or model biological interactions, networks, and data integration using bioinformatics. Modeling of biological systems at all levels, from cellular to ecological, is being facilitated by technological and algorithmic advances. These models provide novel insights into the perturbations in systems that can cause disease, interactions of microbes with various eukaryotic systems, how metabolic networks respond to environmental stresses, etc.	Use and test disciplinary theories, frameworks, and models
24	24.06b Students should be familiar with the techniques used to generate these analyses and should be able to interpret the outputs and use the data to generate novel hypotheses.	Disciplinary approaches

Framework	Element	Theme
24	24.07a Use command-line bioinformatics tools and write simple computer scripts. Most biological datasets (e.g., genomic and proteomic sequences, BLAST results, RNASeq and resulting differential expression data) are available as text files; the most powerful and dynamic way to interact with these datasets is through the command line or shell scripting.	Practical research skills/techniques
24	24.07b Students should be able to manipulate their own data and to create and modify complex data processing and analysis workflows	Data analysis approaches
24	24.08a Describe and manage biological data types, structure, and reproducibility.	Practical research skills/techniques
24	24.08b. This competency addresses two distinct concerns: 1) each of the varied ‘omics fields produces data in formats particular to its needs, and these formats evolve with changes in technologies and refinements in downstream software	Practical research skills/techniques
24	24.08d . Students need to develop an awareness of, and ability to, manipulate different data types given the versioning of formats.	Practical research skills/techniques
24	24.08c. This competency addresses two distinct concerns: 2) all experimental data is subject to error and the user must be cognizant of the need to verify the reproducibility of their data.	Data analysis approaches
24	24.08e They also need to exercise caution, to carry out appropriate statistical analyses on their data as part of normal operating procedures and report the uncertainty of their results, and to provide the relevant information to enable reproduction of their results.	Data analysis approaches
24	24.09 Interpret the ethical, legal, medical, and social implications of biological data. The increasing scale and penetration of human genetic and genomic data has greatly enhanced our ability to identify disease-related loci, druggable targets, etc. and to identify potential genes for replacement with developing techniques. However, with this information also comes many ethical, legal, and social questions; suggested resolutions are often outpaced by the technological advances. As part of their scientific training, students should debate the medicinal, societal, and ethical implications of these information sets and techniques.	Responsible and ethical research conduct
25	25.01 Discipline specific knowledge	Disciplinary content knowledge
25	25.02 Professional knowledge	Disciplinary content knowledge
25	25.03 Inter- and transdisciplinary knowledge and skills	Disciplinary content knowledge
25	25.04 Discipline specific techniques and skills	Practical research skills/techniques
25	25.05 Knowledge (unspecified)	Disciplinary content knowledge
25	25.06 Data collection and analysis	Data analysis approaches
25	25.07 Literature skills	Search disciplinary literature/databases
25	25.08 Research skills (unspecified)	Practical research skills/techniques
25	25.09 Research attitude	General research beliefs
25	25.10 Research methods	Disciplinary approaches
25	25.11 Discipline specific research skills	Practical research skills/techniques
25	25.12 Research conceptualization	Aims, RQs & Hypotheses
25	25.13 Research expertise	Practical research skills/techniques
25	25.14 Interdisciplinary research skills	Practical research skills/techniques
25	25.15 General communication skills (not specified)	Communicating research & research ideas
25	25.16 Written communication skills	Communicating research & research ideas
25	25.17 Oral communication skills	Communicating research & research ideas
25	25.18 Language skills	Communicating research & research ideas
25	25.19 Interdisciplinary communication skills	Communicating research & research ideas

Framework	Element	Theme
25	25.20 Publication skills	Communicating research & research ideas
25	25.21 Disciplinary communication skills	Communicating research & research ideas
25	25.22 Teaching	Omit
25	25.23 Collaboration and teamwork	Collaboration
25	25.24 Networking	Effective interpersonal research relationships
25	25.25 Leadership	Leadership
25	25.26 Supervision	Team management
25	25.27 Project management	Project management
25	25.28 Time management	Time Management
25	25.29 Practitioner scholarship	Translating research to practice
25	25.30 Identity as scholar	Identity
25	25.31 Credibility	Responsible and ethical research conduct
25	25.32 Legitimacy	Responsible and ethical research conduct
25	25.33 Critical thinking	Critical thinking
25	25.34 Problem solving	Problem solving
25	25.35 Cognitive abilities	Critical thinking
25	25.36 Innovation	Creative thinking
25	25.37 Creativity and curiosity	Creative thinking
25	25.38 Confidence	Confidence
25	25.39 Self-efficacy	Confidence
25	25.40 Autonomy and independence	Independence
25	25.41 Resilience	Grit
25	25.42 Agency	Independence
25	25.43 Adaptability	Grit
25	25.44 Self-regulation	Self-Regulation in Research
25	25.45 General life skills and study skills	Professionalism
25	25.46 Advocacy	Outreach
25	25.47 Awareness of injustice and inequality	Culturally aware/relevant research
25	25.48 Social justice-oriented disposition	Inclusive research practice
26	26.01 Managing Yourself: Self-awareness, strengths, accountability	Self Reflective
26	26.02 Managing yourself: implicit bias	Inclusive research practice
26	26.03 Managing yourself: Time management	Time Management
26	26.04 Managing your project: budget management	Budget Management
26	26.05 Managing your project: hiring and interviewing	Team management
26	26.06 Managing your team: building your team	Team management
26	26.07 Managing your team: conflict resolution	Effective interpersonal research relationships
26	26.08 Managing your team: leadership skills	Leadership
27	27.01 Research & Evaluation: Demonstrate familiarity with the current scientific literature in rehabilitation psychology and disability and with disability policy and legal and legislative issues.	Disciplinary content knowledge
27	27.02 Research & Evaluation: Demonstrate knowledge of resources available to locate information relevant to patient care and program development	Search disciplinary literature/databases
27	27.03 Research & Evaluation: Demonstrate understanding of how to use knowledge of research design and statistical methods to identify the level of evidence in published information	Disciplinary approaches

Framework	Element	Theme
27	27.04 Research & Evaluation: Demonstrate familiarity with methods of integrating scientific literature and clinical information in the service of patient care and program development.	Translating research to practice
27	27.05 Research & Evaluation: Demonstrate knowledge of ethical, legal, and regulatory standards for conducting research with human subjects	Responsible and ethical research conduct
27	27.06 Research & Evaluation: Ask focused and operationalizable questions at the individual, team, or systems level.	Aims, RQs & Hypotheses
27	27.07 Research & Evaluation: Use current technological tools and informatics to locate information relevant for patient care and program development related to specialized rehabilitation psychology populations, problems, and procedures	Practical research skills/techniques
27	27.08a Research & Evaluation: Effectively select information that is accurate and applicable to current patients and programs.a. Identify available resources for conducting scientific literature searches.	Search disciplinary literature/databases
27	27.08b Research & Evaluation: Effectively select information that is accurate and applicable to current patients and programs. b. Use effective and efficient search strategies to locate scientific literature relevant to specific questions/patients	Search disciplinary literature/databases
27	27.09a Research & Evaluation: Appraise the research design, statistical methods, and findings of published studies to select data with the highest level of evidence. a. Evaluate the relevance of scientific literature to referral questions and patient population.	Critical evaluation - content knowledge
27	27.09b Research & Evaluation: Appraise the research design, statistical methods, and findings of published studies to select data with the highest level of evidence. b. Evaluate the quality of scientific literature (e.g., study design, analyses, power).	Critical evaluation - content knowledge
27	27.10 Research & Evaluation: Integrate selected scientific literature and clinical information to improve patient care and treatment programs	Inferences and Implications
27	27.11 Research & Evaluation: Evaluate treatment interventions and clinical outcomes in rehabilitation and use this information for continuous quality improvement	Translating research to practice
27	27.12 Research & Evaluation: Present at professional and/or scientific organizations and conferences related to rehabilitation psychology or produce a work product for peer review.	Communicating research & research ideas
27	27.13 Research & Evaluation: Demonstrate the importance of keeping up-to-date in studying and using evidence-based practices	Disciplinary content knowledge
27	27.14 Research & Evaluation: Demonstrate the importance of informed consent and ethical treatment of research subjects	Responsible and ethical research conduct
27	27.15 Research & Evaluation: Demonstrate the importance of honesty and integrity in presenting/publishing research findings.	Responsible and ethical research conduct
27	27.16 Teaching & Supervision: Demonstrate knowledge of methods of teaching specific to rehabilitation psychology and the provision of formal teaching activities.	Omit
27	27.17 Teaching & Supervision: Demonstrate knowledge of methods of providing supervision and evaluating trainees relevant to rehabilitation psychology	Omit
27	27.18 Teaching & Supervision: Demonstrate knowledge of ethics and laws pertaining to teaching and supervisory relationships.	Omit
27	27.19 Teaching & Supervision: Demonstrate knowledge of diversity issues and individual differences pertaining to teaching and supervisory relationships.	Omit

Framework	Element	Theme
27	27.20 Teaching & Supervision: Provide effective teaching within areas of expertise in didactics, journal clubs, case conferences, and professional seminars/colloquia/symposia	Omit
27	27.21 Teaching & Supervision: Effectively translate Rehabilitation Psychology knowledge for interdisciplinary teaching and education.	Omit
27	27.22 Teaching & Supervision: Use feedback from learners to improve teaching	Omit
27	27.23 Teaching & Supervision: Provide appropriate assessment and feedback to practicum and internship students that emphasize skill building in a supportive relationship.	Omit
27	27.24 Teaching & Supervision: Solicit supervisory feedback and use this to modify behavior	Omit
27	27.25 Teaching & Supervision: Identify issues related to individual and cultural diversity and discuss these in supervision	Omit
27	27.26 Teaching & Supervision: Attend on time and actively participate in scheduled training activities (classes, didactics, journal clubs, case conferences, professional seminars/ colloquia/symposia, supervision, mentoring, etc.).	Omit
27	27.27 Teaching & Supervision: Proactively seek and schedule supervision and mentoring.	Omit
27	27.28a Teaching & Supervision: Prepare for teaching and supervision:<br>a. In regard to supervision, has done background reading and thinking about patient care issues and personal learning needs and prioritizes these<br>and brings them for presentation and discussion.	Omit
27	27.28b Teaching & Supervision: Prepare for teaching and supervision:<br>b. In regard to teaching, has done background reading and thinking about the scheduled topic and is prepared to participate in discussion.	Omit
27	27.29a Teaching & Supervision: Take responsibility for learning and development:<br>a. Demonstrate and apply accurate self-reflection and self-appraisal of strengths and weaknesses.	Omit
27	27.29b Teaching & Supervision: Take responsibility for learning and development: b. Identify needed learning and skill development and generate suggestions on how these can be addressed.	Omit
27	27.29c Teaching & Supervision: Take responsibility for learning and development:<br>c. Develop professional growth plan in conjunction with supervisor and mentor.	Omit
27	27.30 Teaching & Supervision: Demonstrate a valuing of diversity and the importance of differing views	Omit
27	27.31 Demonstrate knowledge of APA ethical principles.	Omit
27	27.32 Demonstrate knowledge of key components of professionalism, including appropriate behavior, timely responsibility for tasks, and honesty and<br>integrity	Omit
27	27.33 Demonstrate knowledge of general and rehabilitation-specific legal and health policy issues, including injury and illness prevention, patient<br>advocacy, disability advocacy, financial and administrative aspects of treatment, legislative processes, social and physical environmental<br>accessibility, and disability rights law	Omit

Framework	Element	Theme
27	27.34 Maintain patient confidentiality	Omit
27	27.35a. Manage personal affairs in a way that does not interfere with professional activities.	Omit
27	27.35b. Complete assignments and responsibilities carefully, thoroughly, and on time.	Omit
27	27.35c. Prioritize tasks and display effective time management skills to be efficient and effective.	Omit
27	27.35d. Work beyond usual duties when necessary to provide appropriate care for patients.	Omit
27	27.35e. Seek assistance when work load is too heavy.	Omit
27	27.35f. Communicate with supervisors about barriers to effective work	Omit
27	27.36 Identify ethical, legal, regulatory, and conflict of interest issues that arise and seek appropriate consultation	Omit
27	27.37 Advocate for persons with disability in health-care settings and for rehabilitation services in legal and health policy settings	Omit
27	27.38 Is able to discuss and apply relevant APA guidelines for providers of psychological services:<br>a. Guidelines for Assessment of and Intervention With Persons With Disabilities<br>b. Guidelines on Multicultural Education, Training, Research, Practice, and Organizational Change for Psychologists<br>c. Guidelines for the Evaluation of Dementia and Age-Related Cognitive Decline.<br>d. Guidelines for Psychological Practice with Older Adults<br>e. Guidelines for Psychological Practice with Girls and Women.<br>f. Guidelines for Psychological Practice with Lesbian, Gay, and Bisexual Clients	Omit
27	27.39 Discuss and apply relevant Americans with Disabilities Act guidelines	Omit
27	27.40 Actively educate self about local and state resources for individuals with disabilities	Omit
27	27.41 Actively provide appropriate care for self.	Omit
27	27.42 Demonstrate a commitment to professionalism, including appropriate behavior, timely responsibility for tasks, and honesty and integrity	Omit
27	27.43 Demonstrate a belief in and emphasis on the human worth of persons with impairment or disability and the importance of their integration into the society at large.	Omit
27	27.44 Demonstrate an understanding of and respect for diversity in faculty, trainees, patients, and others in a manner that reflects psychology's ethical principles and professional standards.	Omit
27	27.45 Demonstrate a commitment to the ethical principles of Beneficence and Nonmaleficence, Fidelity and Responsibility, Integrity, Justice, and Respect for People's Rights and Dignity	Omit
28	28.01 Differentiates between primary and secondary sources, and recognizes how their use and importance vary depending upon the legal problem or issue.	Disciplinary content knowledge
28	28.02 Identifies and uses the most effective secondary sources to obtain background information, to gain familiarity with terms of art, and to put primary sources in context.	Search disciplinary literature/databases

Framework	Element	Theme
28	28.03 Recognizes differences in the weight of authority among sources and applying that knowledge to the legal research problem.	Disciplinary content knowledge
28	28.04 Distinguishes between federal, state, and local systems of government; and understanding the processes and the interrelationships among them on all levels.	Disciplinary content knowledge
28	28.05 Knows which legal information is produced, organized, and disseminated across levels and branches of government.	Disciplinary content knowledge
28	28.06 Identifies appropriate resources to locate the legislative, regulatory, and judicial law produced by the respective government bodies.	Search disciplinary literature/databases
28	28.07 Understands and distinguishes between different types of primary law sources, and the weight, reliability, and binding or persuasive authority of each source.	Critical evaluation - content knowledge
28	28.08 Recognizes that there are diverse structural frameworks for the various legal systems within the global community.	Disciplinary content knowledge
28	28.09 Recognizes basic similarities, differences, and interrelationships among and between various types of legal regimes, e.g., United States law, foreign law, and international law.	Disciplinary content knowledge
28	28.10 Identifies information resources that will increase depth and breadth of knowledge regarding a specific legal system.	Search disciplinary literature/databases
28	28.11 Recognizes that other countries and supranational organizations may produce, organize, and disseminate their legal information in different ways, and knows how to find the needed information for a particular legal system.	Disciplinary content knowledge
28	28.12 Identifies and analyzes the appropriate legal issues that need to be researched.	Aims, RQs & Hypotheses
28	28.13 Recognizes the authority or authorities governing particular legal issues.	Disciplinary content knowledge
28	28.14 Knows which print or electronic, primary or secondary, sources contain appropriate and current content on the issue being researched.	Search disciplinary literature/databases
28	28.15 Recognizes how tools facilitate research tasks due to content or organization, such as use of controlled vocabulary, synopses, annotations, or headnotes.	Disciplinary approaches
28	28.16 Knows how to check the content of sources and validate the completeness and currency of the selected sources.	Practical research skills/techniques
28	28.17 Supplements or validates preliminary results with additional tools.	Inferences and Implications
28	28.18 Articulates the precise legal issues that need to be researched, whether in the context of:<br>a. traditional litigation practice,<br>b. regulatory practice, or<br>c. transactional practice.	Aims, RQs & Hypotheses
28	28.19 Develops an appropriate research plan for each discrete issue.	Disciplinary approaches
28	28.20 Knows how to appropriately use available resources to research and understand the relative advantages of different methods of finding information.<br>a. Differentiates among various available online search platforms to employ those that are best suited to the task at hand, and<br>b. Understands the operation of both free and subscription search platforms to skillfully craft appropriate search queries.	Search disciplinary literature/databases

Framework	Element	Theme
28	28.21 Identifies the most cost-efficient sources, calculating cost of use against time on research.	Budget Management
28	28.22 Understands the necessity of validating case holdings through the use of citators such as Shepard's, KeyCite, or other citation-based methods of updating case law.	Disciplinary approaches
28	28.23 Analyzes research results using prior knowledge and experience on the topic in particular, as well as one's general knowledge of legal principles.	Interpret results
28	28.24 Recognizes the benefits of requesting assistance from knowledgeable individuals, or an institution's knowledge management system.	Disciplinary approaches
28	28.25 Understands when to stop the research proces	Disciplinary content knowledge
28	28.26 Records all pertinent information for future reference, such as:<br>a. resources and methods used,<br>b. information considered, and<br>c. reasons for selecting or rejecting various authorities or resources.	Record keeping and data storage
28	28.27 Understands and utilizing proper citation forms.	Communicating research & research ideas
28	28.28 Consistently applies criteria to evaluate the reliability of information, including but not limited to<br>a. Authority,<br>b. Credibility;<br>c. Currency; and<br>d. Authenticity	Critical evaluation - content knowledge
28	28.29 Understands that these criteria are relevant for both print and online, and legal and non-legal, sources.	Disciplinary content knowledge
28	28.30 Understands that there are costs associated with legal research, regardless of type, publisher, or format.	Budget Management
28	28.31 Demonstrates cognizance of the intersection of cost and efficiency in the selection of information format, and exercising professional judgment to choose the best source to serve the research parameters.	Budget Management
28	28.32 Understands the costs and benefits of mediated and disintermediated searching, and using this knowledge to revise research strategies when necessary.	Search disciplinary literature/databases
28	28.33 Clarifies or refines the research question as needed.	Aims, RQs & Hypotheses
28	28.34 Updates or expands the research.	Inferences and Implications
28	28.35 Identifies and addresses any contradictory authority.	Inferences and Implications
28	28.36 Synthesizes legal doctrine by examining cases similar, but not identical, to cases that are the current focus of research, in order to articulate how courts should apply current authoritative and relevant case law.	Inferences and Implications
28	28.37 Uses research results to craft or support arguments that resolve novel legal issues lacking precedent, when appropriate.	Inferences and Implications
28	28.38 Understands research as a recursive process, and expanding or narrowing research queries after discovering unanticipated results.	Disciplinary content knowledge
28	28.39 Reflects on the successes or failures of prior strategies for integrating new information into the analysis; and utilizing concepts, theories, and facts from prior research to continue the process.	Inferences and Implications
28	28.40 Identifies historical sources or scholarship from other disciplines relevant to resolving a specific issue.	Search disciplinary literature/databases

Framework	Element	Theme
28	28.41 Recognizing when specific questions within the larger research problem have not been answered with the information compiled, by either:<br>a. Recognizing when the ultimate questions presented have not been fully answered through the research already obtained, or<br>b. Realizing when sufficient research has been completed to address the legal issue or information need.	Inferences and Implications
28	28.42 Identifies unresolved issues and incorporates analogous background as appropriate if research has not clearly resolved all ambiguities or uncertainties within the issue posed.	Inferences and Implications
28	28.43 Identifies scholarship from other disciplines relevant to resolving a specific issue.	Disciplinary content knowledge
28	28.44 Understands how courts or other legal decision-makers have applied materials from other disciplines in the past, and determines when material from these disciplines might be persuasive in resolving a particular issue.	Disciplinary content knowledge
28	28.45 Locates background information to help answer a legal issue or need by using resources such as:<br>a. records of constitutional conventions,<br>b. legislative histories,<br>c. administrative histories,<br>d. trial or appellate briefs, or<br>e. economic, policy, business-specific, social, psychological, historical, or other inter-disciplinary research.	Search disciplinary literature/databases
28	28.46 Cites authority consistent with locally accepted rules, ensuring that cited references can be located by the reader.	Communicating research & research ideas
28	28.47 Organizes and integrates content, quotations, or forms, and paraphrases in a manner that supports the argument, brief, analysis, or transaction.<br>a. Chooses an appropriate communication format and style for the intended audience; and<br>b. Integrates charts, maps, or photos into the document or presentation for maximally persuasive effect, when appropriate.	Communicating research & research ideas
28	28.48 Comprehends and complies with laws and organizational (firm, school, court) rules on access to information resources and storage and dissemination of information.	Record keeping and data storage
28	28.49 Understands intellectual property issues such as licensing, copyright, and fair use of copyrighted material.	Responsible and ethical research conduct
28	28.50 Accurately articulates privacy, confidentiality, security, diligence, and other ethical issues related to research and practice in accordance with the Model Rules of Professional Conduct, the Model Code of Professional Responsibility, or the prevailing local law governing legal ethics.	Responsible and ethical research conduct
28	28.51 Uses citation of sources to respect authors' intellectual property rights and accurately indicates where the words and ideas of others have been used.	Responsible and ethical research conduct
28	28.52 Comprehends and complies with license and subscription agreements.	Responsible and ethical research conduct
28	28.53 Understands local requirements for continuous legal education.	Professionalism
28	28.54 Affirmatively undertakes training on research platforms as new iterations reach the market.	Practical research skills/techniques

Framework	Element	Theme
28	28.55 Comprehends that legal research skills, like legal standards, are "moving targets" subject to further refinement and development as the universe of legal knowledge (and legal research tools) expands.	Disciplinary approaches
29	29.01 systematically reviewing the state of research,	Search disciplinary literature/databases
29	29.02 evaluating relevant literature,	Critical evaluation - content knowledge
29	29.03 identifying contradictory findings,	Critical evaluation - content knowledge
29	29.04 – identifying research need.	Aims, RQs & Hypotheses
29	29.05 formulating and operationalizing research questions/hypotheses,	Aims, RQs & Hypotheses
29	29.06 planning the research process,	Disciplinary approaches
29	29.07 selecting appropriate research methods,	Disciplinary approaches
29	29.08 applying adequate methods.	Practical research skills/techniques
29	29.09 reflecting on implications of research results,	Inferences and Implications
29	29.10 reflecting on methodological limitations,	Disciplinary approaches
29	29.11 reflecting on practical implications,	Inferences and Implications
29	29.12 reflecting on ethical implications	Responsible and ethical research conduct
29	29.13 writing academic publications,	Communicating research & research ideas
29	29.14 presenting research findings.	Communicating research & research ideas
29	29.15 Content knowledge of central/key theories,	Disciplinary content knowledge
29	29.16 Content knowledge of central research methods	Disciplinary approaches
29	29.17 content knowledge of previous findings	Disciplinary content knowledge
29	29.18 content knowledge of standards of communications in academic research	Communicating research & research ideas
30	30.01 Use of catalogues, descriptor books and bibliographic records.	Search disciplinary literature/databases
30	30.02 Formulation of a scientific problem, research objectives, and research hypotheses.	Aims, RQs & Hypotheses
30	30.03 Selection of the population, the sample, and the type of sampling to be used	Disciplinary approaches
30	30.04 Selection, development, and application of methods, techniques, and instruments.	Disciplinary approaches
30	30.05 Analysis and processing of information through different statistical techniques.	Data analysis approaches
30	30.06 Interpretation and discussion of results presented in tables and graphs.	Interpret results
30	30.07 Drawing up conclusions and recommendations	Inferences and Implications
30	30.08 Writing final research reports.	Communicating research & research ideas
31	31.01 Conducting searches for research literature related to your research project (this does NOT include programming or technical guidance unless directly from a peer-reviewed published article)	Search disciplinary literature/databases
31	31.02 Reading research articles in the discipline (i.e., physics/chemistry)	Disciplinary content knowledge
31	31.03 Reading research articles in the relevant sub-discipline (i.e., particle physics/organic chemistry)	Disciplinary content knowledge
31	31.04 Identifying the theoretical purpose to why given methods or techniques are used in the literature	Disciplinary content knowledge
31	31.05 Interpreting and critiquing the results and findings presented in literature	Interpret results
31	31.06 Identifying further information necessary to support research-related results in the literature	Inferences and Implications
31	31.07 Interpreting visual representations of data (i.e., graphs, diagrams, and tables) provided in research literature	Interpret results
31	31.08 Discussion of research literature within ‘informal’ group setting (i.e., research group or journal club)	Communicating research & research ideas
31	31.09 Create written or oral summaries of research article	Communicating research & research ideas

Framework	Element	Theme
31	31.10 Developing your own research questions or hypotheses	Aims, RQs & Hypotheses
31	31.11 Developing your own research plan	Disciplinary approaches
31	31.12 Using basic research techniques (i.e., those often learned in early classes—data entry, weighing of samples, etc.)	Practical research skills/techniques
31	31.13 Using advanced research techniques and methods in your field of study	Practical research skills/techniques
31	31.14 Trouble shooting theoretical/technical errors in research during data collection	Problem solving
31	31.15 Computer programming for data collection	Practical research skills/techniques
31	31.16 Computer programming for statistical analysis/modeling of numerical data	Practical research skills/techniques
31	31.17 Computer programming for analysis of non-numerical data (e.g., image processing, chemical analysis)	Practical research skills/techniques
31	31.18 Qualitative/descriptive analysis of results	Data analysis approaches
31	31.19 Statistical analysis of research results using established stat software	Data analysis approaches
31	31.20 Interpreting statistical analysis of research in the field	Interpret results
31	31.21 Interpreting research-related results	Interpret results
31	31.22 Representing data in a visual form common for the research field (i.e., the construction of graphs, tables, and diagrams)	Visualize data
31	31.23 Trouble shooting theoretical/technical errors in research after interpreting the data	Problem solving
31	31.24 Discussion of research plans or results within 'informal' group setting (i.e., research group or journal club)	Communicating research & research ideas
31	31.25 Writing up research methods	Communicating research & research ideas
31	31.26 Writing up results	Communicating research & research ideas
31	31.27 Writing up a discussion of the results	Communicating research & research ideas
31	31.28 Making an oral presentation on research you participated in within a 'formal' group setting (i.e., professional meeting, undergraduate research conference)	Communicating research & research ideas
31	31.29 Understanding of the overarching discipline (i.e., chemistry/physics) in which your research is conducted	Disciplinary content knowledge
31	31.30 Understanding of the sub-discipline (i.e., particle physics, organic chemistry) in which your research is conducted	Disciplinary content knowledge
31	31.31 Understanding of the elements of work involved in science research	Disciplinary approaches
31	31.32 Understanding the process of science in your field (i.e., "how science research is done")	Disciplinary approaches
31	31.33 Understanding the social or cultural practices of your field (i.e., "how scientists act or behave")	Professionalism
31	31.34 Working independently to complete "basic" research tasks (e.g., data entry, weighing of samples, etc.	Independence
31	31.35 Working independently to complete advanced research techniques and methods in your field of study	Independence
31	31.36 Working in the lab setting with other individuals to complete tasks	Collaboration
31	31.37 Discussing results with mentors	Communicating research & research ideas
31	31.38 Suggesting next steps in the research process	Inferences and Implications
32	32.01 Communication: Uses and understands professional and discipline-specific language.	Communicating research & research ideas
32	32.02 Communication: Expresses ideas in an organized, clear, concise, and accurate manner.	Communicating research & research ideas
32	32.03 Communication: Writes clearly and effectively in discipline-specific formats.	Communicating research & research ideas

Framework	Element	Theme
32	32.04 Creativity: Brings new insights to the problem at hand.	Creative thinking
32	32.05 Creativity: Shows ability to approach problems from different perspectives.	Creative thinking
32	32.06 Creativity: Combines information in new ways and/or demonstrates intellectual resourcefulness.	Creative thinking
32	32.07 Creativity: Effectively connects multiple ideas/approaches	Creative thinking
32	32.08 Autonomy: Demonstrates the ability to work independently and identify when input, guidance, and feedback are needed.	Independence
32	32.09 Autonomy: Accepts constructive criticism and applies feedback effectively.	Receive research feedback
32	32.10 Autonomy: Displays high level of confidence in ability to meet challenges	Confidence
32	32.11 Autonomy: Uses time well to ensure work gets accomplished and meets deadlines	Time Management
32	32.12 Ability to deal with obstacles: Learns from and is not discouraged by setbacks and unforeseen events.	Grit
32	32.13 Ability to deal with obstacles: Shows flexibility and a willingness to take risks and try again	Grit
32	32.14 Practice & Process of Inquiry: Demonstrates ability to formulate questions and hypotheses within the discipline.	Aims, RQs & Hypotheses
32	32.15 Practice & Process of Inquiry: Demonstrates ability to properly identify and/or generate reliable data.	Practical research skills/techniques
32	32.16 Practice & Process of Inquiry: Shows understanding of how knowledge is generated, validated, and communicated within the discipline	Disciplinary approaches
32	32.17 Nature of disciplinary knowledge: Shows understanding of the way practitioners think within the discipline (e.g., as an earth scientist, sociologist, or artist) and view the world around them.	Disciplinary approaches
32	32.18 Nature of disciplinary knowledge: Shows understanding of the criteria for determining what is valued as a contribution to the discipline	Disciplinary content knowledge
32	32.19 Nature of disciplinary knowledge: Shows understanding of important current individuals within the discipline.	Disciplinary content knowledge
32	32.20 Critical Thinking & Problem Solving: Trouble-shoots problems, searches for ways to do things more effectively, and generates, evaluates, and selects between alternatives	Problem solving
32	32.21 Critical Thinking & Problem Solving: Recognizes discipline-specific problems and challenges established thinking when appropriate.	Critical evaluation - content knowledge
32	32.22 Critical Thinking & Problem Solving: Recognizes flaws, assumptions, and missing elements in arguments.	Critical evaluation - content knowledge
32	32.23 Understanding Ethical Conduct: Shows understanding and respect for intellectual property rights	Responsible and ethical research conduct
32	32.24 Understanding Ethical Conduct: Predicts, recognizes, and weighs the risks and benefits of the project for others.	Responsible and ethical research conduct

Framework	Element	Theme
32	32.25 Understanding Ethical Conduct: Recognizes the severity of creating, modifying, misrepresenting, or misreporting data, including omission or elimination of data/findings or authorship.	Responsible and ethical research conduct
32	32.26 Intellectual Development: Demonstrates growth from basic to more complex thinking in the discipline	Disciplinary content knowledge
32	32.27 Intellectual Development: Recognizes that problems are often more complicated than they first appear to be and the most economical solution is usually preferred over convoluted explanations.	Disciplinary content knowledge
32	32.28 Intellectual Development: Approaches problems from a perspective that there can be more than one right explanation or model or even none at all	Problem solving
32	32.29 Intellectual Development: Displays accurate insight into the extent of his/her own knowledge and understanding and an appreciation for what isn't known	Self Reflective
32	32.30 Culture of Scholarship: Is involved in the scholarly community of the discipline and/or professional societies.	Professionalism
32	32.31 Culture of Scholarship: Behaves with a high level of collegiality and ethical responsibility.	Effective interpersonal research relationships
32	32.32 Content Knowledge Skills/Methodology: Displays detailed and accurate knowledge of key facts and concepts.	Disciplinary content knowledge
32	32.33 Content Knowledge Skills/Methodology: Displays a thorough grasp of relevant research methods and is clear about how these methods apply to the research project being undertaken.	Disciplinary approaches
32	32.34 Content Knowledge Skills/Methodology: Demonstrates an advanced level of requisite skills.	Practical research skills/techniques
33	33.01 Argument	Aims, RQs & Hypotheses
33	33.02 Theorizing	Aims, RQs & Hypotheses
33	33.03 Framework	Use and test disciplinary theories, frameworks, and models
33	33.04 Knowledge Creation	Aims, RQs & Hypotheses
33	33.05 Analysis and interpretation	Data analysis approaches
33	33.06 research paradigm	Disciplinary approaches
33	33.07 questioning and problematising of accepted concepts	Critical evaluation - content knowledge
33	33.08 being able to mount a defensible argument	Aims, RQs & Hypotheses
33	33.09 conceptual and theoretical framework development	Use and test disciplinary theories, frameworks, and models
33	33.10 develping questions, design, data anlysis, conclusions, so that conceptual and theoretical conclusions will be produced.	Aims, RQs & Hypotheses
34	34.01 Analysis Skills	Data analysis approaches
34	34.02 Big Picture	Aims, RQs & Hypotheses
34	34.03 Conceptual Knowledge	Disciplinary content knowledge
34	34.04 Critical Thinking	Critical thinking
34	34.05 Data Interpretation	Interpret results
34	34.06 Defining the Problem	Aims, RQs & Hypotheses
34	34.07 Literature Review	Search disciplinary literature/databases

Framework	Element	Theme
34	34.08 Math/Statistical Skills	Practical research skills/techniques
34	34.09 Research Design	Disciplinary approaches
35	35.01 Introducting/setting the study in context	Cultural context of research
35	35.02 appropriate integrating primary literature	Disciplinary content knowledge
35	35.03 establishing testable hypotheses	Aims, RQs & Hypotheses
35	35.04 using appropriate experimental controls and replications	Disciplinary approaches
35	35.05 experimental design	Disciplinary approaches
35	35.06 selecting data for anlysis	Data analysis approaches
35	35.07 data analysis	Data analysis approaches
35	35.08 presenting results	Communicating research & research ideas
35	35.09 basing conclusions on results	Interpret results
35	35.10 identifying alternative explanations of findings	Inferences and Implications
35	35.11 identifying limitations of the study	Disciplinary approaches
35	35.12 discussing implications of the findings	Inferences and Implications
36	36.01 Q26—Conduct rigorous qualitative investigation	Practical research skills/techniques
36	36.02 Q29—Identify qualitative data analysis procedures	Data analysis approaches
36	36.03 Q27—Identify qualitative data collection procedures	Disciplinary approaches
36	36.04 Q23—Ground research question in the literature	Aims, RQs & Hypotheses
36	36.05 Q28—Implement qualitative data collection procedures	Practical research skills/techniques
36	36.06 Q30—Employ qualitative data analysis procedures	Data analysis approaches
36	36.07 Q25—Identify qualitative research design	Disciplinary approaches
36	36.08 Q22—Grounded in theoretical framework	Use and test disciplinary theories, frameworks, and models
36	36.09 Q33—Address threats to trustworthiness	Disciplinary approaches
36	36.10 Q31—Interpret qualitative results	Interpret results
36	36.11 Q24—Paradigmatic assumptions and research goals	Aims, RQs & Hypotheses
36	36.12 Q21—Construct qualitative research question	Aims, RQs & Hypotheses
36	36.13 Q32—Identify threats to trustworthiness	Disciplinary approaches
36	36.14 Q40—Identify quantitative data collection procedures	Disciplinary approaches
36	36.15 Q41—Implement quantitative data collection procedure	Practical research skills/techniques
36	36.16 Q36—Develop quantitative research question	Aims, RQs & Hypotheses
36	36.17 Q34—Construct quantitative research question	Aims, RQs & Hypotheses
36	36.18 Q46—Identify threats to validity in quantitative study	Disciplinary approaches
36	36.19 Q44—Interpret quantitative results collection procedure	Interpret results
36	36.20 Q45—Differentiate statistical and practical signal	Interpret results
36	36.21 Q35—Grounding quantitative question in theory	Aims, RQs & Hypotheses
36	36.22 Q37—Understand epistemological assumptions	Disciplinary content knowledge
36	36.23 Q19—Select data collect instruments	Disciplinary approaches
36	36.24 Q16—Operationally defining variables	Disciplinary approaches
36	36.25 Q18—Interpret psychometrics of instruments	Interpret results
36	36.26 Q49—Implement research ethics	Responsible and ethical research conduct
36	36.27 Q51—Implement ethical standards with humans	Responsible and ethical research conduct
36	36.28 Q50—Know ethical standards with humans	Responsible and ethical research conduct
36	36.29 Q48—Know research ethics	Responsible and ethical research conduct
36	36.30 Q52—Demonstrate cultural competence	Culturally aware/relevant research
36	36.31 Q54—Implement ethics on research team	Responsible and ethical research conduct
36	36.32 Q53—Know authorship processes	Authorship

Framework	Element	Theme
36	36.33 Q68—Develop implications	Inferences and Implications
36	36.34 Q69—Present research	Communicating research & research ideas
36	36.35 Q65—Construct report without grammar errors	Communicating research & research ideas
36	36.36 Q64—Align report with APA manual	Communicating research & research ideas
36	36.37 Q62—Construct report aligned with APA manual	Communicating research & research ideas
36	36.38 Q66—Disseminate quantitative report in journal	Communicating research & research ideas
36	36.39 Q63—Present position with citations	Communicating research & research ideas
36	36.40 Q57—Clear and concise results	Communicating research & research ideas
36	36.41 Q58—Compare finding to the literature	Inferences and Implications
36	36.42 Q59—Identify potential limitations	Disciplinary approaches
36	36.43 Q2—Identify theories in literature	Disciplinary content knowledge
36	36.44 Q3—Recognize gaps in literature	Critical evaluation - content knowledge
36	36.45 Q1—Locate relevant literature	Search disciplinary literature/databases
36	36.46 Q6—Develop framework to guide study	Use and test disciplinary theories, frameworks, and models
36	36.47 Q8—Generate meaningful research inquiry areas	Aims, RQs & Hypotheses
36	36.48 Q5—Identify implications from an article	Inferences and Implications
36	36.49 Q7—Construct a rationale for a study	Aims, RQs & Hypotheses
36	36.50 Q4—Recognize limitations of studies	Disciplinary approaches
36	36.51 Q11—Identify probability sampling procedures	Disciplinary approaches
36	36.52 Q14—Implement nonprobability sampling procedure	Practical research skills/techniques
36	36.53 Q13—Identify nonprobability sampling procedures	Disciplinary approaches
36	36.54 Q12—Implement probability sampling procedures	Practical research skills/techniques
37	37.01 Identify the appropriate study designs to measure the burden of cardiovascular diseases in the population and determine associations between risk factors and disease burden.	Disciplinary approaches
37	37.02 Describe the role of potential confounders and sources of bias that interfere with the ability to determine causal associations in population science.	Disciplinary approaches
37	37.03 Describe the underlying pathophysiology of cardiovascular disease across vascular beds.	Disciplinary content knowledge
37	37.04 Identify risk factors for cardiovascular diseases, including the social determinants of disease.	Disciplinary content knowledge
37	37.05 Discuss strategies to prevent and treat cardiovascular diseases at the clinical and population level.	Translating research to practice
37	37.06 Assemble, evaluate, and synthesize the scientific literature.	Search disciplinary literature/databases
37	37.07 Collect data using rigorous and reproducible data collection instruments.	Practical research skills/techniques
37	37.08 Manage data collection in the context of multidisciplinary research.	Project management
37	37.09 Calculate measures of disease burden and association using statistical analysis software.	Data analysis approaches
37	37.10 Interpret data analysis for oral and written presentation to scientific audiences.	Communicating research & research ideas
37	37.11 Demonstrate clear and concise scientific writing that includes the following components: a hypothesis, description of methods, interpretation of results, and summary of findings in the context of the scientific literature.	Communicating research & research ideas
37	37.12 Propose compelling scientific arguments and methodologies to present in grant applications.	Communicating research & research ideas
37	37.13 Discuss the scientific process and findings with lay audiences using appropriately adapted language.	Outreach

Framework	Element	Theme
37	37.14 Conduct research according to responsible conduct of research standards with protections in place for human subjects.	Responsible and ethical research conduct
37	37.15 Complete work products in a timely manner. Demonstrate collegiality and professionalism in all interactions.	Time Management
38	38.01 Literature reviews	Search disciplinary literature/databases
38	38.02 ethics in educational research	Responsible and ethical research conduct
38	38.03 research designs	Disciplinary approaches
38	38.04 sampling	Disciplinary approaches
38	38.05 data collection methodologies	Disciplinary approaches
38	38.06 data analysis procedures	Data analysis approaches
38	38.07 data reporting	Communicating research & research ideas
38	38.08 scholarly writing practices	Communicating research & research ideas
39	39.01 Career & Self-Development: skills, knowledge, and dispositions gained to support one's career aspirations.	Career development
39	39.02 Career & Self-Development: developing one's professional identity	Identity
39	39.03 Communication: students frame and share their work with varied audiences	Communicating research & research ideas
39	39.04 Communication: Students gain experience sharing their work in a formal setting outside the classroom verbally, visually, and in written form	Communicating research & research ideas
39	39.05 Critical Thinking: Observation, analysis, interpretation, inference and problem-solving	Inferences and Implications
39	39.06 Equity & Inclusion: flexibility in adapting to diverse environments	Inclusive research practice
39	39.07 Equity & Inclusion: keeping an open mind to divers ideas and new ways of thinking	Creative thinking
39	39.08 Equity & Inclusion: solicit and use feedback from multiple cultural perspectives to make inclusive and equity-minded decisions”	Inclusive research practice
39	39.09 Equity & Inclusion: “actively contribute to inclusive and equitable practices that influence individual and systemic change.”	Inclusive research practice
39	39.10 Equity & Inclusion: “identify resources and eliminate barriers resulting from individual and systemic racism, inequities, and biases,”	Inclusive research practice
39	39.11 Equity & Inclusion: address systems of privilege that limit opportunities for members of historically marginalized communities,	Inclusive research practice
39	39.12 Leadership:seek out and leverage diverse resources and feedback from others to inform direction,”	Receive research feedback
39	39.13 Leadership: “use innovative thinking to go beyond traditional methods,	Creative thinking
39	39.14 Leadership: “plan, initiate, manage, complete, and evaluate projects,”	Project management
39	39.15 Leadership: students serve as role models	Leadership
39	39.16 Professionalism: emonstrating dependability and accountability; ability to meet deadlines, act independently and with confidence; ability to work through ambiguity and obstacles; ability to prioritize and accomplish tasks; and a commitment to a larger, external goal.	Professionalism
39	39.17 Teamwork: “be[ing] accountable for individual and team responsibilities and deliverables,” “collaborat[ing] with others to achieve common goals,”	Collaboration
39	39.18 Technology: lab techniques, technical skills, data analysis skills	Practical research skills/techniques
39	39.19 Technology: leveraging technologies for teams to interact across distances	Collaboration
40	40.01 The student understands the purpose, concept, and criteria for choosing a topic for research	Disciplinary content knowledge

Framework	Element	Theme
40	40.02 The student has an understanding of the different type of research method, study design	Disciplinary approaches
40	40.03 The student has the knowledge of doing a literature review and can develop a research question	Search disciplinary literature/databases
40	40.04 The student knows different elements of a research protocol	Disciplinary approaches
40	40.05 The student has an understanding of basic statistics including classification of variables, measures of central tendency and dispersion	Data analysis approaches
40	40.06 The student can formulate a simple research question, frame objectives aligned to the research question and can choose an appropriate study design to address the research question	Aims, RQs & Hypotheses
40	40.07 The student can contribute to develop a questionnaire for data collection	Disciplinary approaches
40	40.08 The student can calculate the sample size for a prevalence study	Practical research skills/techniques
40	40.09 The student can perform probability sampling and can recruit study sample	Practical research skills/techniques
40	40.10 The student can contribute to writing an informed consent form	Responsible and ethical research conduct
40	40.11 The student can contribute to developing the overall operational plan of the study	Disciplinary approaches
40	40.12 The student can write a protocol	Disciplinary approaches
40	40.13 The student can extract and carry out basic analysis of key data sets (using MS excel) by producing frequencies, tables, graphs, calculating rate, ratio, proportion, and cross-tabulations; interprets the key findings from this	Data analysis approaches
40	40.14 Identify and articulate whether or not any conclusions drawn from analyses of data are valid and based on the material provided	Interpret results
40	40.15 Shows the ability to Communicate with community members	Outreach
40	40.16 show the ability to Interact appropriately with peers	Effective interpersonal research relationships
40	40.17 shows the ability to Behave and conduct herself in a professional manner in the community	Professionalism
40	40.18 Demonstrate an understanding of ethics and regulation of research while dealing with human subjects	Responsible and ethical research conduct
40	40.19 Ensures confidentiality, privacy, and autonomy of research participants Understand the need for ethical approval to be obtained before research activities are initiated	Responsible and ethical research conduct
41	41.01 Learn basic research practices	Practical research skills/techniques
41	41.02 Progress toward completion of independent research project	Independence
41	41.03 Maintain a lab notebook.	Record keeping and data storage
41	41.04 Present scientific findings in written format.	Communicating research & research ideas
41	41.05 Present scientific findings in oral format	Communicating research & research ideas
41	41.06 Critique oral presentations of peers	Give feedback on research
41	41.07 Collaborate with peers in lab.	Collaboration
41	41.08 Develop mentoring skills.	Mentoring
41	41.09 Present at a research symposium.	Communicating research & research ideas
41	41.10 Manage projects and personnel.	Project management
41	41.11 Develop teaching style.	Omit
41	41.12 Learn pedagogical practices	Omit
42	42.01 Understands the knowledge base of the relevant fi elds (theories, methods, techniques). [ks]	Disciplinary content knowledge
42	42.02 Understands the structure of the relevant fi elds, and the connections between sub-fi elds. [ks]	Disciplinary content knowledge

Framework	Element	Theme
42	42.03 Has knowledge of and some skill in the way in which truth-fi nding and the development of theories and models take place in the relevant fi elds. [ks]	Disciplinary content knowledge
42	42.04 Has knowledge of and some skill in the way in which interpretations (texts, data, problems, results) take place in the relevant fi elds. [ks]	Interpret results
42	42.05 Has knowledge of and some skill in the way in which experiments, gathering of data and simulations take place in the relevant fi elds. [ks]	Disciplinary approaches
42	42.06 Has knowledge of and some skill in the way in which decision-making takes place in the relevant fi elds. [ks]	Disciplinary content knowledge
42	42.07 Is aware of both the presuppositions of the standard methods and their importance. [ksa]	Disciplinary approaches
42	42.08 Is able (with supervision) to spot gaps in his / her own knowledge, and to revise and extend it through study. [ks]	Self Reflective
42	42.09 Is able to reformulate ill-structured research problems. Also takes account of the system boundaries in this. Is able to defend the new interpretation against involved parties. [ksa]	Aims, RQs & Hypotheses
42	42.10 Is observant, and has the creativity and the capacity to discover in apparently trivial matters certain connections and new viewpoints. [ksa]	Creative thinking
42	42.11 Is able (with supervision) to produce and execute a research plan. [ks]	Project management
42	42.12 Is able to work at different levels of abstraction. [ks]	Critical thinking
42	42.13 Understands, where necessary, the importance of other disciplines (interdisciplinarity). [ka]	Disciplinary content knowledge
42	42.14 Is aware of the changeability of the research process through external circumstances or advancing insight. [ka]	Disciplinary content knowledge
42	42.15 Is able to assess research within the discipline on its usefulness. [ks]	Critical evaluation - content knowledge
42	42.16 Is able (with supervision) to contribute to the development of scientifi c knowledge in one or more areas of the disciplines concerned. [ks]	Practical research skills/techniques
42	42.17 Has creativity and synthetic skills with respect to design problems. [ksa]	Creative thinking
42	42.18 Is able to work at different levels of abstraction including the system level. [ks]	Critical thinking
42	42.19 Is aware of the changeability of the design process through external circumstances or advancing insight. [ka]	Disciplinary content knowledge
42	42.20 Is able to integrate existing knowledge in a design. [ks]	Disciplinary approaches
42	42.21 Has the skill to take design decisions, and to justify and evaluate these in a systematic manner. [ks]	Disciplinary approaches
42	42.22 Is inquisitive and has an attitude of lifelong learning. [ka]	Curiosity
42	42.23 Has a systematic approach characterised by the development and use of theories, models and interpretations. [ksa]	Use and test disciplinary theories, frameworks, and models
42	42.24 Has the knowledge and the skill to use, justify and assess as to their value models for research and design (model understood broadly: from mathematical model to scale-model). Is able to adapt models for his or her own use. [ks]	Use and test disciplinary theories, frameworks, and models
42	42.25 Has insight into the nature of science and technology (purpose, methods, differences and similarities between scientifi c fi elds, nature of laws, theories, explanations, role of the experiment, objectivity etc.). [k]	Disciplinary approaches
42	42.26 Has insight into the scientifi c practice (research system, relation with clients, publication system, importance of integrity etc.). [k]	Disciplinary content knowledge
42	42.27 Is able to document adequately the results of research and design with a view to contributing to the development of knowledge in the fi eld and beyond. [ksa]	Record keeping and data storage

Framework	Element	Theme
42	42.28 Is able (with supervision) to critically refl ect on his or her own thinking, decision making, and acting and to adjust these on the basis of this refl ection. [ks]	Self Reflective
42	42.29 Is able to reason logically within the fi eld and beyond; both 'why' and 'what-if' reasoning. [ks]	Critical evaluation - content knowledge
42	42.30 Is able to recognise modes of reasoning (induction, deduction, analogy etc.) within the fi eld. [ks	Critical evaluation - content knowledge
42	42.31 Is able to ask adequate questions, and has a critical yet constructive attitude towards analysing and solving simple problems in the fi eld. [ks]	Aims, RQs & Hypotheses
42	42.32 Is able to form a well-reasoned opinion in the case of incomplete or irrelevant data. [ks]	Inferences and Implications
42	42.33 Is able to take a standpoint with regard to a scientifi c argument in the fi eld. [ksa]	Inferences and Implications
42	42.34 Possesses basic numerical skills, and has an understanding of orders of magnitude. [ks]	Practical research skills/techniques
42	42.35 Is able to communicate in writing about the results of learning, thinking and decision making with colleagues and non-colleagues. [ks]	Communicating research & research ideas
42	42.36 Is able to communicate verbally about the results of learning, thinking and decision making with colleagues and non-colleagues. [ks]	Communicating research & research ideas
42	42.37 Is able to follow debates about both the fi eld and the place of the fi eld in society. [ks]	Disciplinary content knowledge
42	42.38 Is characterised by professional behaviour. This includes: drive, reliability, commitment, accuracy, perseverance and independence. [ksa]	Professionalism
42	42.39 Is able to perform project-based work: is pragmatic and has a sense of responsibility; is able to deal with limited sources; is able to deal with risks; is able to compromise. [ksa]	Practical research skills/techniques
42	42.40 Is able to work within an interdisciplinary team. [ks]	Collaboration
42	42.41 Has insight into, and is able to deal with, team roles and social dynamics. [ks]	Effective interpersonal research relationships
42	42.42 Understands relevant (internal and external) developments in the history of the fi elds concerned. This includes the interaction between the internal developments (of ideas) and the external (social) developments. [ks]	Disciplinary content knowledge
42	42.43 Is able to analyse and to discuss the social consequences (economical, social, cultural) of new developments in relevant fi elds with colleagues and non-colleagues. [ks]	Cultural context of research
42	42.44 Is able to analyse the consequences of scientifi c thinking and acting on the environment and sustainable development. [ks]	Inferences and Implications
42	42.45 Is able to analyse and to discuss the ethical and the normative aspects of the consequences and assumptions of scientifi c thinking and acting with colleagues and non-colleagues (both in research and in designing). [ks]	Responsible and ethical research conduct
42	42.46 Has an eye for the different roles of professionals in society. [ks]	Outreach
42	42.47 Has a thorough mastery of parts of the relevant fi elds extending to the forefront of knowledge (latest theories, methods, techniques and topical questions). [ks]	Disciplinary content knowledge
42	42.48 Looks actively for structure and connections in the relevant fi elds. [ksa]	Inferences and Implications
42	42.49 Is able to refl ect on standard methods and their presuppositions; is able to question these; is able to propose adjustments, and to estimate their implications. [ksa]	Disciplinary approaches
42	42.50 Given the process stage of the research problem, chooses the appropriate level of abstraction. [ksa]	Disciplinary approaches

Framework	Element	Theme
42	42.51 Is able, and has the attitude to, where necessary, draw upon other disciplines in his or her own research. [ksa]	Disciplinary approaches
42	42.52 Is able to assess research within the discipline on its scientifi c value. [ksa]	Critical evaluation - content knowledge
42	42.53 Is able, and has the attitude, where necessary, to draw upon other disciplines in his or her own design. [ksa]	Disciplinary approaches
42	42.54 Is able to formulate new research questions on the basis of a design problem. [ks]	Aims, RQs & Hypotheses
42	42.55 Is able to identify and take in relevant developments. [ksa]	Inferences and Implications
42	42.56 Is able to critically examine existing theories, models or interpretations in the area of his or her graduation subject. [ksa]	Use and test disciplinary theories, frameworks, and models
42	42.57 Has great skill in, and affinity with the use, development and validation of models; is able consciously to choose between modelling techniques. [ksa]	Use and test disciplinary theories, frameworks, and models
42	42.58 Is able to recognise fallacies. [ks]	Critical evaluation - content knowledge
43	43.01 Critical but balanced reading of primary literature	Critical evaluation - content knowledge
43	43.02 design of experiments with appropriate controls	Disciplinary approaches
44	44.01 Thinking like a scientist	Disciplinary content knowledge
44	44.02 Communicating results	Communicating research & research ideas
44	44.03 Using tools and technology	Practical research skills/techniques
44	44.04 Collaboration and Interactivity	Collaboration
44	44.05 Iteration (repeating experiments and refining ideas)	Practical research skills/techniques
44	44.06 Discovery: sciences is a cumulative process; individuals must contribute small pieces of knowledge to come to better understandings of larger systems and phenomena.	Disciplinary content knowledge
44	44.07 Engaging in broadly relevant research	Practical research skills/techniques
45	45.01 Systems thinking	Critical thinking
45	45.02 Research self-efficacy	Confidence
45	45.03 interdisciplinary collaboration	Collaboration
45	45.04 effective communication	Effective interpersonal research relationships
45	45.05 research design & management (basic project organization/manangement skills)	Project management
46	46.01 Demonstrate beginning competence in accessing appropriate and relevant information	Search disciplinary literature/databases
46	46.02 Demonstrate beginning competence in accessing research-based evidence relevant to identified clinical problems	Search disciplinary literature/databases
46	46.03 Critically appraise research evidence to apply findings to clinical practice	Critical evaluation - content knowledge
46	46.04 Select appropriate clinical problems	Aims, RQs & Hypotheses
46	46.05 Read and evaluate research reports and data-based articles, synthesize findings, and evaluate their applicability to practice	Critical evaluation - content knowledge

Framework	Element	Theme
46	46.06a Read and critically appraise data-based literature	Critical evaluation - content knowledge
46	46.06b Synthesize findings and evaluate their applicability to practice	Inferences and Implications
46	46.07 Demonstrate skills necessary to design and implement an evidence-based clinical protocol (capstone project)	Disciplinary approaches
46	46.08 Demonstrate the ability to critically appraise research and propose alternate designs	Critical evaluation - content knowledge
46	46.09 Plan independent, original research with guidance from dissertation committee members Integrate research findings in a particular topic area	Aims, RQs & Hypotheses
46	46.10 Identify gaps in the literature to substantiate the significance of proposed research	Critical evaluation - content knowledge
46	46.11a Read and critically evaluate the literature	Critical evaluation - content knowledge
46	46.11b synthesize the literature,	Inferences and Implications
46	46.11c determine where the next level of evidence is needed when addressing a problem	Critical evaluation - content knowledge
46	46.12a Conduct independent, original research to provide the evidence for practice	Practical research skills/techniques
46	46.12b Disseminate research findings/knowledge gained	Communicating research & research ideas
47	47.01 Content area epertise	Disciplinary content knowledge
47	47.02 data management	Record keeping and data storage
47	47.03 evaluation theory	Use and test disciplinary theories, frameworks, and models
47	47.04 field data collection	Practical research skills/techniques
47	47.05 instrument development	Disciplinary approaches
47	47.06 literature reviews	Search disciplinary literature/databases
47	47.07 multivariate statistical analysis	Data analysis approaches
47	47.08 presentation skills	Communicating research & research ideas
47	47.09 project and/or team manangement	Project management
47	47.10 project planning	Project management

Framework	Element	Theme
47	47.11 proposal writing	Communicating research & research ideas
47	47.12 qualitiative analysis	Data analysis approaches
47	47.13 qualitative methods	Disciplinary approaches
47	47.14 relating to clients and stakeolders	Effective interpersonal research relationships
47	47.15 report writing	Communicating research & research ideas
47	47.16 research design	Disciplinary approaches
47	47.17 syntax writing	Practical research skills/techniques
47	47.18 univariate statistical analysis	Data analysis approaches
47	47.19 writing for publications	Communicating research & research ideas
48	48.01 Assess sources of bias and variation in published studies; assess threats to study validity (bias) including problems with sampling, recruitment, randomization, and comparability of study groups	Critical evaluation - content knowledge
48	48.02 Propose study designs for addressing a clinical or translational research question	Disciplinary approaches
48	48.03 Describe the basic principles and practical importance of random variation, systematic error, sampling error, measurement error, hypothesis testing, type I and type II errors, and confi dence limits	Data analysis approaches
48	48.04 Compute sample size, power, and precision for comparisons of two independent samples with respect to continuous and binary outcomes	Disciplinary approaches
48	48.05 Explain the uses, importance, and limitations of early stopping rules in clinical trials	Responsible and ethical research conduct
48	48.06a Describe the concepts and implications of reliability and validity of study measurements	Disciplinary approaches
48	48.06b evaluate the reliability and validity of measures	Disciplinary approaches
48	48.07a Calculate basic epidemiologic measures;	Practical research skills/techniques
48	48.07b draw appropriate inferences from epidemiologic data	Inferences and Implications
48	48.08 Scrutinize the assumptions behind different statistical methods and their corresponding limitations	Disciplinary approaches
48	48.09 Describe preferred methodological alternatives to commonly used statistical methods when assumptions are not met	Disciplinary approaches
48	48.10 Distinguish among the different measurement scales and the implications for selection of statistical methods to be used on the basis of these distinctions	Disciplinary approaches
48	48.11 Generate simple descriptive and inferential statistics that fit the study design chosen and answer research question	Data analysis approaches
48	48.12 Apply descriptive techniques commonly used to summarize public health data; apply common statistical methods for inference; apply descriptive and inferential methodologies according to the type of study design for answering a particular research question	Practical research skills/techniques

Framework	Element	Theme
48	48.13 Describe the uses of meta-analytic methods	Disciplinary approaches
48	48.14a Communicate clinical and translational research fi ndings to difference groups of individuals, including colleagues, students, the lay public, and the media;	Communicating research & research ideas
48	48.14b write summaries of scientifi c information for use in the development of clinical health care policy	Translating research to practice
48	48.15a Interpret results of statistical analyses found in public health studies;	Interpret results
48	48.15b develop written and oral presentations on the basis of statistical analyses for both public health professionals and educated lay audiences	Communicating research & research ideas
48	48.16 Describe size of the effect with a measure of precision	Interpret results
48	48.17 Describe the study sample, including sampling methods, the amount and type of missing data, and the implications for generalizability	Inferences and Implications
48	48.18 Interpret results in light of multiple comparisons	Interpret results
48	48.19 Identify inferential methods appropriate for clustered, matched, paired, or longitudinal studies	Disciplinary approaches
48	48.20 Describe adjusted inferential methods appropriate for the study design, including examination of interaction	Disciplinary approaches
48	48.21 Describe statistical methods appropriate to address loss to follow-up	Data analysis approaches
49	49.01 dynamic interactions between populations and contextual variations (age, gender, ethnicity, culture, etc.) on health behavior and health outcomes	Data analysis approaches
49	49.02 mechanistic and mediational pathways between contextual, psychosocial, and biological phenomena as they relate to health promotion, illness prevention, and disease progression	Data analysis approaches
49	49.03 scientific foundations and methods of psychology and allied health disciplines (e.g., epidemiology, physiology).	Disciplinary content knowledge
49	49.04 strengths and potential pitfalls of role relationships that characterize interdisciplinary collaborative research.	Collaboration
49	49.05 legal–ethical issues relating to interdisciplinary research	Responsible and ethical research conduct
49	49.06 apply diverse methodologies to address contextual, psychosocial, and biological processes as they relate to health promotion, illness prevention, and disease progression.	Disciplinary approaches
49	49.07 select, apply, and interpret data-analytic strategies that are best suited to the diverse research questions and levels of analysis characteristic of health psychology.	Data analysis approaches
49	49.08 accurately and efficiently communicate research findings in a manner that is consistent with the highest standards within the profession	Communicating research & research ideas
50	50.01 An understanding of the importance of self-reflection and assessment	Self Reflective

Framework	Element	Theme
50	50.02 The ability to effectively accomplish selfdirected learning	Independence
50	50.03 The ability to evaluate information critically and make evidence-based decisions	Critical evaluation - content knowledge
50	50.04 The ability to explain how research is created, disseminated, interpreted, and applied	Disciplinary approaches
51	51.01 Administer psychological assessment measures	Practical research skills/techniques
51	51.02 Score psychological assessment measures	Practical research skills/techniques
51	51.03 Select appropriate assessment measures	Disciplinary approaches
51	51.04 Integrate and interpret psychological assessments by writing an integrative report	Communicating research & research ideas
51	51.05 Conceptualize a psychological assessment case	Aims, RQs & Hypotheses
51	51.06 Understand the basic theory of psychological assessment	Disciplinary content knowledge
51	51.07 . Clearly and accurately communicate the results of psychological tests through verbal feedback	Communicating research & research ideas
51	51.08 Design a methodologically sound research study	Disciplinary approaches
51	51.09 Conduct appropriate statistical analyses to evaluate results of a research study	Data analysis approaches
51	51.10 Write up an APA-style research report	Communicating research & research ideas
51	51.11 Find appropriate literature on a research topic	Search disciplinary literature/databases
52	52.01 – Acquire professional perspective: Understand and analyze the history and values of the discipline and its relationship to other fields while demonstrating an ability to read, interpret, and critique the core literature	Disciplinary content knowledge
52	52.02 Analyze problems: Analyze, understand, abstract, and model a specific biomedical problem in terms of data, information, and knowledge components	Use and test disciplinary theories, frameworks, and models
52	52.03 Produce solutions: Use the problem analysis to identify and understand the space of possible solutions and generate designs that capture essential aspects of solutions and their components.	Disciplinary approaches
52	52.04 – Articulate the rationale: Defend the specific solution and its advantage over competing options.	Disciplinary approaches
52	52.05 Implement, evaluate, and refine: Carry out the solution (including obtaining necessary resources and managing projects), evaluate it, and iteratively improve it	Project management
52	52.06 – Innovate: Create new theories, typologies, frameworks, representations, methods, and processes to address biomedical informatics problems	Use and test disciplinary theories, frameworks, and models
52	52.07 – Work collaboratively: Team effectively with partners within and across disciplines.	Collaboration
52	52.08 Educate, disseminate, and discuss: Communicate effectively to students and to other audiences in multiple disciplines in persuasive written and oral form	Communicating research & research ideas
52	52.09 Prerequisite knowledge and skills: Students must be familiar with biological, biomedical, and population health concepts and problems including common research problems	Disciplinary content knowledge

Framework	Element	Theme
52	52.10 – Fundamental knowledge: Understand the fundamentals of the field in the context of the effective use of biomedical data, information, and knowledge	Disciplinary content knowledge
52	52.11 Procedural knowledge and skills: For substantive problems related to scientific inquiry, problem solving, and decision making, apply, analyze, evaluate, and create solutions based on biomedical informatics approaches	Disciplinary approaches
52	52.12 Understand and analyze complex biomedical informatics problems in terms of data, information, and knowledge.	Disciplinary approaches
52	52.13 Apply, analyze, evaluate, and create biomedical informatics methods that solve substantive problems within and across biomedical domains.	Disciplinary approaches
52	52.14 Relate such knowledge and methods to other problems within and across levels of the biomedical spectrum	Inferences and Implications
52	52.15 Theories: Understand and apply syntactic, semantic, cognitive, social, and pragmatic theories as they are used in biomedical informatics	Use and test disciplinary theories, frameworks, and models
52	52.16 Typology: Understand, and analyze the types and nature of biomedical data, information, and knowledge.	Disciplinary content knowledge
52	52.17 Frameworks: Understand, and apply the common conceptual frameworks that are used in biomedical informatics.	Use and test disciplinary theories, frameworks, and models
52	52.18 – Knowledge representation: Understand and apply representations and models that are applicable to biomedical data, information, and knowledge.	Use and test disciplinary theories, frameworks, and models
52	52.19 Methods and processes: Understand and apply existing methods (eg, simulated annealing) and processes (eg, goaloriented reasoning) used in different contexts of biomedical informatics.	Disciplinary approaches
52	52.20 Prerequisite knowledge and skills: Assumes familiarity with data structures, algorithms, programming, mathematics, statistics.	Practical research skills/techniques
52	52.21 – Fundamental knowledge: Understand and apply technological approaches in the context of biomedical problems.	Practical research skills/techniques
52	52.22 Procedural knowledge and skills: For substantive problems, understand and apply methods of inquiry and criteria for selecting and utilizing algorithms, techniques, and methods	Disciplinary approaches
52	52.23 Prerequisite knowledge and skills: Familiarity with fundamentals of social, organizational, cognitive, and decision sciences	Disciplinary content knowledge
52	52.24 Fundamental knowledge: Understand and apply knowledge in Design, evaluation, social, behavior, communication, and organizational sciences, ethical, legal, social issues, economic, social, and organizational context of biometical research, pharmaceutical and biotechnology industries, medical instrumentation, healthcare, and public health	Disciplinary content knowledge
52	52.25 Procedural knowledge and skills: Apply, analyze, evaluate, and create systems approaches to the solution of substantive problems in biomedical informatics	Practical research skills/techniques

Framework	Element	Theme
52	52.26 Analyze complex biomedical informatics problems in terms of people, organizations, and socio-technical systems.	Cultural context of research
52	52.27 Understand the challenges and limitations of technological solutions	Inferences and Implications
52	52.28 – Design and implement systems approaches to biomedical informatics applications and interventions	Disciplinary approaches
52	52.29 – Evaluate the impact of biomedical informatics applications and interventions in terms of people, organizations, and sociotechnical systems	Cultural context of research
52	52.30 – Relate solutions to other problems within and across levels of the biomedical spectrum.	Inferences and Implications
53	53.01 Use theories and methods of multiple disciplines in developing integrated theoretical and research frameworks	Use and test disciplinary theories, frameworks, and models
53	53.02 Integrate concepts and methods from multiple disciplines in designing interdisciplinary research protocols.	Inferences and Implications
53	53.03 Investigate hypotheses through interdisciplinary research.	Practical research skills/techniques
53	53.04 Draft funding proposals for interdisciplinary research programs in partnership with scholars from other disciplines.	Communicating research & research ideas
53	53.05 Disseminate interdisciplinary research results both within and outside his or her discipline.	Communicating research & research ideas
53	53.06 Author publications with scholars from other disciplines	Collaboration
53	53.07 Advocate interdisciplinary research in developing initiatives within a substantive area of study.	Outreach
53	53.08 Express respect for the perspectives of other disciplines.	Inclusive research practice
53	53.09 Read journals outside of his or her discipline	Disciplinary content knowledge
53	53.10 Communicate regularly with scholars from multiple disciplines.	Collaboration
53	53.11 Share research from his or her discipline in language meaningful to an interdisciplinary team.	Communicating research & research ideas
53	53.12 Modify his or her own work or research agenda as a result of interactions with colleagues from fi elds other than his or her own	Receive research feedback
53	53.13 Present interdisciplinary research at venues representing more than one discipline.	Communicating research & research ideas
53	53.14 Engage colleagues from other disciplines to gain their perspectives on research problems.	Collaboration
53	53.15 Interact in training exercises with scholars from other disciplines.	Career development
53	53.16 Attend scholarly presentations by members of other disciplines	Disciplinary content knowledge
53	53.17 Collaborate respectfully and equitably with scholars from other disciplines to develop interdisciplinary research frameworks.	Collaboration
54	54.01 Understand cultural factors at the individual, familial, and societal level that affect pediatric chronic illness conditions, disease symptoms, complications, and medical management	Cultural context of research
54	54.02 Understand sociocultural factors that affect health promotion, illness prevention, and disease progression	Culturally aware/relevant research
54	54.03 Understand sociocultural factors that might discourage or prevent participation in research and how to circumvent these	Culturally aware/relevant research

Framework	Element	Theme
54	54.04 Familiarity with all levels of influence on child and family functioning	Disciplinary content knowledge
54	54.05 Familiarity with normal and abnormal development in cognitive, emotional, behavioral, and physical domains	Disciplinary content knowledge
54	54.06 Understand potential interactions across influences and how to account for these in research design	Disciplinary approaches
54	54.07 Knowledge of scientific foundations/methods of psychology as well as the foundations and methods of related disciplines as they apply to pediatric psychology	Disciplinary content knowledge
54	54.08 Familiarity with research designs particularly relevant to pediatric psychology (e.g., longitudinal, randomized clinical trials, structural equation modeling, and single-case design) and program evaluation designs	Disciplinary approaches
54	54.09 Familiarity with evidence-based practice	Translating research to practice
54	54.10 Knowledge foundations and methods of related disciplines as they apply to pediatric psychology	Disciplinary content knowledge
54	54.11 Utilize clinical experiences to inform research hypotheses and design	Aims, RQs & Hypotheses
54	54.12 Participate in research-related collaborations within and across medical and other settings including ways of developing and maintaining these relationships	Collaboration
54	54.13 Knowledge and practice exposure through relevant clinical training to learn about potential legal and ethical dilemmas related to working with children and families	Responsible and ethical research conduct
54	54.14 Knowledge and practice exposure to appropriately address legal and ethical dilemmas into research design and methodology	Responsible and ethical research conduct
54	54.15 Knowledge and ability to utilize single-subject, cross-sectional, and longitudinal designs within pediatric populations	Practical research skills/techniques
54	54.16 Apply appropriate methodologies	Disciplinary approaches
54	54.17 Apply knowledge of pediatric chronic illness/injury condition, symptoms, complications, and disease prevention/ management to methodological decisions	Disciplinary approaches
54	54.18 Specialized statistical training focusing on broad statistical knowledge, consultation, and analysis rather than simple mastery of methods	Data analysis approaches
54	54.19 Interpreting and dissemination research findings to other psychologists, medical teams, the broad scientific community, and the lay community public through manuscripts, posters, oral presentations, and interviews with the mass media	Communicating research & research ideas
54	54.20 Understand and is familiar with language used by other disciplines	Disciplinary content knowledge
54	54.21 Targeted clinical practice to obtain clinical experiences to develop and refine research questions	Aims, RQs & Hypotheses

Framework	Element	Theme
54	54.22 Demonstrate respect for other disciplines and their research methodologies while maintaining rigorous standards	Inclusive research practice
54	54.23 Understand research role and expected contribution (i.e., principal investigator, co-investigator, consultant, and program evaluator)	Disciplinary content knowledge
54	54.24 Integrate research and practice to facilitate skill development in program development, evaluation, and intervention research	Translating research to practice
54	54.25 Apply research findings in clinical-care settings	Translating research to practice
55	55.01 The information literate student defines and articulates the need for information.	Aims, RQs & Hypotheses
55	55.02 The information literate student identifies a variety of types and formats of potential sources for information.	Search disciplinary literature/databases
55	55.03 The information literate student considers the costs and benefits of acquiring the needed information.	Budget Management
55	55.04 The information literate student reevaluates the nature and extent of the information need.	Critical evaluation - content knowledge
55	55.05 The information literate student selects the most appropriate investigative methods or information retrieval systems for accessing the needed information.	Disciplinary approaches
55	55.06 The information literate student constructs and implements effectivelydesigned search strategies.	Disciplinary approaches
55	55.07 The information literate student retrieves information online or in person using a variety of methods.	Search disciplinary literature/databases
55	55.08 The information literate student refines the search strategy if necessary	Search disciplinary literature/databases
55	55.09 The information literate student extracts, records, and manages the information and its sources.	Record keeping and data storage
55	55.10 The information literate student summarizes the main ideas to be extracted from the information gathered.	Inferences and Implications
55	55.11 The information literate student articulates and applies initial criteria for evaluating both the information and its sources.	Critical evaluation - content knowledge
55	55.12 The information literate student synthesizes main ideas to construct new concepts.	Inferences and Implications
55	55.13 The information literate student compares new knowledge with prior knowledge to determine the value added, contradictions, or other unique characteristics of the information	Inferences and Implications
55	55.14 The information literate student determines whether the new knowledge has an impact on the individual's value system and takes steps to reconcile differences.	Inferences and Implications
55	55.15 The information literate student validates understanding and interpretation of the information through discourse with other individuals, subject-area experts, and/or practitioners.	Receive research feedback
55	55.16 The information literate student determines whether the initial query should be revised.	Inferences and Implications

Framework	Element	Theme
55	55.17 The information literate student applies new and prior information to the planning and creation of a particular product or performance.	Inferences and Implications
55	55.18 The information literate student revises the development process for the product or performance.	Disciplinary approaches
55	55.19 The information literate student communicates the product or performance effectively to others.	Communicating research & research ideas
55	55.20 The information literate student understands many of the ethical, legal and socio-economic issues surrounding information and information technology.	Responsible and ethical research conduct
55	55.21 The information literate student follows laws, regulations, institutional policies, and etiquette related to the access and use of information resources	Responsible and ethical research conduct
55	55.22 The information literate student acknowledges the use of information sources in communicating the product or performance.	Communicating research & research ideas
56	56.01 Literature Search: used key words, search dates, cited seminal/landmark studies	Search disciplinary literature/databases
56	56.02 Hypothesis/AIm Objective: clearly explained hypothesis, aim or objective, discussed hypothesis, aim or objective in relation to data, may have hypothesis decision	Aims, RQs & Hypotheses
56	56.03 Statistical Analysis: may have described statistics as it related to design; sample size & power; statistical limitations	Disciplinary approaches
56	56.04 Presentation skills: clear & concise; answered technical questions; logical flow, reiterated results, may recommend future direction	Communicating research & research ideas
56	56.05 Personal conduct: well-prepared, professional attired, well-mannered and respectful towards others, arrived early, polished presentation	Professionalism
57	57.01 Describe the current state of knowledge about a biomedical, clinical, or public health problem	Disciplinary content knowledge
57	57.02 Defend the clinical and public health implications of a given research hypothesis	Aims, RQs & Hypotheses
57	57.03 Develop appropriate methods to recruit and retain study participants for a selected research design	Disciplinary approaches
57	57.04 Identify important outcome measures for incorporation into patient-oriented clinical trial design.	Disciplinary approaches
57	57.05 Generate a plan for data security/management.	Record keeping and data storage
57	57.06 Identify barriers in translating research discoveries into meaningful changes in human health	Translating research to practice
57	57.07 Develop an approach to overcome barriers in translating research to humans.	Translating research to practice
57	57.08 Design appropriate, ethically sound, and hypothesis-driven clinical studies.	Aims, RQs & Hypotheses
57	57.09 Select the appropriate statistical approach for the interpretation of preclinical and clinical datasets.<br>Define bias in clinical and translational research.	Data analysis approaches
57	57.10 Identify federal and non-federal agencies and programmatic initiatives aimed at translating research to clinical care of patients	Budget Management
57	57.11 Defend a written research proposal that describes specific research aims, significance, innovation, and approach for a human clinical trial.	Communicating research & research ideas
57	57.12 Assess the clinical implications of scientific information.	Translating research to practice
57	57.13 Develop a therapeutic protocol/guideline for medication-related issues or management	Translating research to practice

Framework	Element	Theme
57	57.14 Demonstrate knowledge of the standards of professional and ethical conduct established to guide researchers in protecting the rights, well-being, and dignity in the recruitment and retainment of human subjects in clinical research.	Responsible and ethical research conduct
57	57.15 Give examples of the informed consent process including an understanding of the risk/benefit criteria and its impact on the patient/volunteer.	Responsible and ethical research conduct

S3c. Framework Citations

Framework Number	Citation
2	Verderame MF, Freedman VH, Kozlowski LM, McCormack WT. Competency-based assessment for the training of P students and early-career scientists. Pewsey E, editor. eLife. 2018;7: e34801. doi:10.7554/eLife.34801
3	Willison J, O'Regan K, Kuhn SK. Researcher Skill Development Framework (US English Edition). 2018. Available frc https://commons.und.edu/cgi/viewcontent.cgi?article=1004&context=oers
4	NPA Core Competencies. (nd). Available from: https://www.nationalpostdoc.org/page/CoreCompetencies
5	Pelaez N, Anderson T, Gardner S, Yin Y, Abraham J, Bartlett E, et al. The Basic Competencies of Biological Experimentation: Concept-Skill Statements. PIBERG Instructional Innovation Materials. 2016. Available from: https://docs.lib.purdue.edu/pibergiim/4
6	Clemmons AW, Timbrook J, Herron JC, Crowe AJ. BioSkills Guide: Development and National Validation of a Tool fc Interpreting the Vision and Change Core Competencies. LSE. 2020;19: ar53. doi:10.1187/cbe.19-11-0259
7	Cui Q, Harshman J. Qualitative Investigation to Identify the Knowledge and Skills That U.S.-Trained Doctoral Chemis Require in Typical Chemistry Positions. J Chem Educ. 2020;97: 1247–1255. doi:10.1021/acs.jchemed.9b01027
8	The Evaluate UR method: Outcomes: Categories and Compoenents. [Internet]. 9 Mar 2023 Available from: https://serc.carleton.edu/evaluateur/method/outcomes.html
9	Butz AR, Branchaw JL. Entering Research Learning Assessment (ERLA): Validity Evidence for an Instrument to Mea Undergraduate and Graduate Research Trainee Development. LSE. 2020;19: ar18. doi:10.1187/cbe.19-07-0146
10	Bray R, Boon S. Towards a framework for research career development: An evaluation of the UK's Vitae Researcher Development Framework. International Journal for Researcher Development. 2011;2: 99–116. doi:10.1108/17597511111212709
11	Ahmadi M, Sheikhtaheri A, Tahmasbi F, Eslami Jahromi M, Rangraz Jeddi F. A competency framework for Ph.D. programs in health information management. Int J Med Inform. 2022;168: 104906. doi:10.1016/j.ijmedinf.2022.10490
12	Charumbira MY, Berner K, Louw QA. Research competencies for undergraduate rehabilitation students : a scoping r Afr J Health Prof Educ. 2021;13: 52–58. doi:10.7196/AJHPE.2021.v13i1.1229
13	Drotar D, Cortina S, Crosby LE, Hommel KA, Modi AC, Pai ALH. Competency-based postdoctoral research training f clinical psychologists: An example and implications. Train Educ Prof Psychol. 2015;9: 92–98. doi:10.1037/tep000003
14	Willison J, O'Regan K. Commonly known, commonly not known, totally unknown: a framework for students becoming researchers. High Educ Res Dev. 2007;26. doi:10.1080/07294360701658609
15	Duru P, Örsal Ö. Development of the Scientific Research Competency Scale for nurses. J Res Nurs. 2021;26: 684–7 doi:10.1177/17449871211020061
16	Gess C, Geiger C, Ziegler M. Social-Scientific Research Competency. Eur J Psychol Assess. 2019;35: 737–750. doi:10.1027/1015-5759/a000451
17	Harsh J, J. Esteb J, V. Maltese A. Evaluating the development of chemistry undergraduate researchers' scientific thi skills using performance-data: first findings from the performance assessment of undergraduate research (PURE) instrument. Chem Educ Res Pract. 2017;18: 472–485. doi:10.1039/C6RP00222F
18	Hayes-Harb R, St. Andre M, Shannahan M. Assessment of Undergraduate Research Learning Outcomes: Poster Presentations as Artifacts. SPUR. 2020;3: 55–61. doi:10.18833/spur/3/4/10
19	Kariyana I, Sonn ,Reynold A., and Marongwe N. Objectivity of the subjective quality: Convergence on competencies expected of doctoral graduates. Cheng M, editor. Cogent Educ. 2017;4: 1390827. doi:10.1080/2331186X.2017.1390
20	Lindsay H, Floyd A. Experiences of using the researching professional development framework. Stud Grad Postdr E 2019;10: 54–68. doi:10.1108/SGPE-02-2019-049

Framework Number	Citation
21	Miller L, Brushett S, Ayn C, Furlotte K, Jackson L, MacQuarrie M, et al. Developing a Competency Framework for Population Health Graduate Students Through Student and Faculty Collaboration. Pedagogy Health Promot. 2021;7: 280–288. doi:10.1177/2373379919859607
22	Nowell L, Dhingra S, Kenny N, Jacobsen M, Pexman P. Professional learning and development framework for postd scholars. Stud Grad Postdr Educ. 2021;12: 353–370. doi:10.1108/SGPE-10-2020-0067
23	Qiu C, Feng X, Reinhardt JD, Li J. Development and psychometric testing of the Research Competency Scale for Nu Students: An instrument design study. Nurse Educ Today. 2019;79: 198–203. doi:10.1016/j.nedt.2019.05.039
24	Sayres MAW, Hauser C, Sierk M, Robic S, Rosenwald AG, Smith TM, et al. Bioinformatics core competencies for undergraduate life sciences education. PLoS ONE. 2018;13: e0196878. doi:10.1371/journal.pone.0196878
25	Senekal JS, Munnik E, Frantz JM. A systematic review of doctoral graduate attributes: Domains and definitions. Fron Educ. 2022;7. doi:10.3389/feduc.2022.1009106
26	Steen K, Vornhagen J, Weinberg ZY, Boulanger-Bertolus J, Rao A, Gardner ME, et al. A structured professional development curriculum for postdoctoral fellows leads to recognized knowledge growth. PLoS ONE. 2021;16: e0260 doi:10.1371/journal.pone.0260212
27	Stiers W, Barisa M, Stucky K, Pawlowski C, Van Tubbergen M, Turner AP, et al. Guidelines for competency develop and measurement in rehabilitation psychology postdoctoral training. Rehabil Psychol. 2015;60: 111–122. doi:10.1037/a0038353
28	Talley NB. Are You Doing It Backward? Improving Information Literacy Instruction Using the AALL Principles and Standards for Legal Research Competency, Taxonomies, and Backward Design. Law Libr J. 2014;106: 47–68. Avail from: https://heinonline.org/HOL/P?h=hein.journals/llj106&i=47
29	Böttcher F, Thiel F. Evaluating research-oriented teaching: a new instrument to assess university students' research competences. High Educ. 2018;75: 91–110. doi:10.1007/s10734-017-0128-y
30	Ipanaqué-Zapata M, Figueroa-Quiñones J, Bazalar-Palacios J, Arhuis-Inca W, Quiñones-Negrete M, Villarreal-Zega Research skills for university students' thesis in E-learning: Scale development and validation in Peru. Heliyon. 2023 e13770. doi:10.1016/j.heliyon.2023.e13770
31	Maltese A, Harsh J, Jung E. Evaluating Undergraduate Research Experiences—Development of a Self-Report Tool. Sci (Basel). 2017;7: 87. doi:10.3390/educsci7040087
32	Singer J, Zimmerman B. Evaluating a Summer Undergraduate Research Program: Measuring Student Outcomes a Program Impact. Counc Undergrad Res Q. 2012;32: 40–47. Available from: https://digitalcommons.buffalostate.edu/cgi/viewcontent.cgi?article=1004&context=earth_sciences_facpub
33	Kiley M, and Wisker G. Threshold concepts in research education and evidence of threshold crossing.High Educ Re 2009;28: 431–441. doi:10.1080/07294360903067930
34	Feldon DF, Maher MA, Hurst M, Timmerman B. Faculty Mentors', Graduate Students', and Performance-Based Assessments of Students' Research Skill Development. Am Educ Res J. 2015;52: 334–370. doi:10.3102/0002831214549449
35	Feldon DF, Litson K, Jeong S, Blaney JM, Kang J, Miller C, et al. Postdocs' lab engagement predicts trajectories of F students' skill development. Proc Natl Acad Sci U S A. 2019;116: 20910–20916. doi:10.1073/pnas.1912488116
36	Swank JM, Lambie GW. Development of the Research Competencies Scale. Meas Eval Couns Dev. 2016;49: 91–1 doi:10.1177/0748175615625749
37	Carnethon MR, Neubauer LC, Greenland P. Competency-Based Postdoctoral Education. Circulation. 2019;139: 310 doi:10.1161/CIRCULATIONAHA.118.037494
38	Lambie GW, Hayes BG, Griffith C, Limberg D, Mullen PR. An Exploratory Investigation of the Research Self-Efficacy Interest in Research, and Research Knowledge of Ph.D. in Education Students. Innov High Educ. 2014;39: 139–153 doi:10.1007/s10755-013-9264-1

Framework Number	Citation
39	Mekolichick J. Mapping the Impacts of Undergraduate Research, Scholarship, and Creative Inquiry Experiences to th NACE Career Readiness Competencies. NACE Journal. 2021;82: 34–40. Available from: https://ebiztest.naceweb.org/career-readiness/competencies/mapping-the-impacts-of-undergraduate-research-schola and-creative-inquiry-experiences-to-the-nace-career-readiness-competencies/
40	Patra S, Khan AM. Development and implementation of a competency-based module for teaching research methodo to medical undergraduates. J Educ Health Promot. 2019;8: 164. doi:10.4103/jehp.jehp_133_19
41	Brown AM, Lewis SN, Bevan DR. Development of a structured undergraduate research experience: Framework and implications. Biochem Mol Biol Educ. 2016;44: 463–474. doi:10.1002/bmb.20975
42	Meijers AWM, Borghuis VAJ, Mutsaers EJPJ, Overveld, van CWAM, Perrenet JC. Criteria voor academische bachel master curricula = Criteria for academic bachelor's and master's curricula. 2e, gew. dr. ed. Eindhoven: Technische Universiteit Eindhoven, 2005. 24 p. Available from: https://research.tue.nl/en/publications/criteria-voor-academische-bachelor-en-master-curricula-criteria-f
43	Feldon DF, Rates ,Christopher, and Sun C. Doctoral conceptual thresholds in cellular and molecular biology. Int J Sc Educ. 2017;39: 2574–2593. doi:10.1080/09500693.2017.1395493
44	Brownell SE, and Kloser MJ. Toward a conceptual framework for measuring the effectiveness of course-based undergraduate research experiences in undergraduate biology. Stud High Educ. 2015;40: 525–544. doi:10.1080/03075079.2015.1004234
45	Elder S, Wittman H, Giang A. Building sustainability research competencies through scaffolded pathways for undergraduate research experience. Elementa (Wash D C). 2023;11: 00091. doi:10.1525/elementa.2022.00091
46	Burke LE, Schlenk EA, Sereika SM, Cohen SM, Happ MB, Dorman JS. Developing Research Competence to Suppo Evidence-Based Practice. J Prof Nurs. 2005;21: 358–363. doi:10.1016/j.profnurs.2005.10.011
47	Dewey JD, Montrosse BE, Schröter DC, Sullins CD, MattoxII JR. Evaluator Competencies: What’s Taught Versus W Sought. Am J Eval. 2008;29: 268–287. doi:10.1177/1098214008321152
48	Enders F. Evaluating Mastery of Biostatistics for Medical Researchers: Need for a New Assessment Tool. Clin Trans 2011;4: 448–454. doi:10.1111/j.1752-8062.2011.00323.x
49	France CR, Masters KS, Belar CD, Kerns RD, Klonoff EA, Larkin KT, et al. Application of the competency model to c health psychology.Prof Psychol Res Pr. 2008;39: 573–580. doi:10.1037/0735-7028.39.6.573
50	Hodgson JL, Pelzer JM, Inzana KD. Beyond NAVMEC: Competency-Based Veterinary Education and Assessment o Professional Competencies. J Vet Med Educ. 2013;40: 102–118. doi:10.3138/jvme.1012-092R
51	Kamen C, Veilleux JC, Bangen KJ, VanderVeen JW, Klonoff EA. Climbing the stairway to competency: Trainee perspectives on competency development. Train Educ Prof Psychol. 2010;4: 227–234. doi:10.1037/a0021092
52	Kulikowski CA, Shortliffe EH, Currie LM, Elkin PL, Hunter LE, Johnson TR, et al. AMIA Board white paper: definition biomedical informatics and specification of core competencies for graduate education in the discipline. JAMIA Open. 2012;19: 931–938. doi:10.1136/amiajnl-2012-001053
53	Larson EL, Landers TF, Begg MD. Building Interdisciplinary Research Models: A Didactic Course to Prepare Interdisciplinary Scholars and Faculty. Clin Transl Sci. 2011;4: 38–41. Available: https://ascpt.onlinelibrary.wiley.com/doi/10.1111/j.1752-8062.2010.00258.x
54	Madan-Swain A, Hankins SL, Gilliam MB, Ross K, Reynolds N, Milby J, et al. Applying the Cube Model to Pediatric Psychology: Development of Research Competency Skills at the Doctoral Level. J Pediatr Psychol. 2012;37: 136–14 doi:10.1093/jpepsy/jsr096
55	American Library Association. Information Literacy Competency Standards for Higher Education. Jan 2000. Available https://alair.ala.org/items/294803b6-2521-4a96-a044-96976239e3fb
56	Musial JL, Rubinfeld IS, Parker AO, Reickert CA, Adams SA, Rao S, et al. Developing a Scoring Rubric for Resident Research Presentations: A Pilot Study. J Surg Res. 2007;142: 304–307. doi:10.1016/j.jss.2007.03.060
57	Poloyac SM, Empey KM, Rohan LC, Skledar SJ, Empey PE, Nolin TD, et al. Core Competencies for Research Train the Clinical Pharmaceutical Sciences. Am J Pharm Educ. 2011;75: 27. doi:10.5688/ajpe75227
