# Supplementary material for: The comprehensive researcher development framework (CRDF): Core learning outcomes for research training
Source: PLoS One. 2025 Sep 15;20(9):e0332587. doi: 10.1371/journal.pone.0332587 (PMC12435680; doi:10.1371/journal.pone.0332587)
Supplement: S4 Appendix — (PDF) [file pone.0332587.s005.pdf]

Original Learning Outcomes (LOs)	Decisions before Card Sort 1	LOs used in Card Sort 1	Decisions after Card Sort 1	LOs used in Card sort #2	Decisions after Card Sort 2	Final Version	Final Area	Final numbering
know the fundamental content in their discipline.	Keep	know the fundamental content in their discipline.	Revise	know the fundamental content in their discipline (e.g., frameworks, theories)	Revise	know the fundamental content in their discipline (e.g., frameworks, theories, and models)	Foundational Disciplinary Knowledge	1.01
know the history of knowledge generation in their discipline.	Keep	know the history of knowledge generation in their discipline.	Keep	know the history of knowledge generation in their discipline.	Keep	know the history of knowledge generation in their discipline.	Foundational Disciplinary Knowledge	1.02
know the processes by which new knowledge is generated in their discipline.	Revise	know the processes by which new knowledge is generated and evaluated in their discipline.	Keep	know the processes by which new knowledge is generated and evaluated in their discipline.	Revise	know the processes by which new knowledge is generated and evaluated.	Foundational Disciplinary Knowledge	1.03
ground hypotheses and research questions in established disciplinary theories or frameworks.	Revise	ground hypotheses and research questions in established disciplinary knowledge, theories, or frameworks.	Keep	ground hypotheses and research questions in established disciplinary knowledge, theories, or frameworks.	Revise	ground hypotheses and research questions in established disciplinary knowledge, theories, frameworks or observations.	Foundational Disciplinary Knowledge	1.04
recognize the inferences and implications of research findings on and beyond the discipline.	Keep	recognize the inferences and implications of research findings on and beyond the discipline.	Keep	recognize the inferences and implications of research findings on and beyond the discipline.	Split LO	know inferences and implications of research findings	Foundational Disciplinary Knowledge	1.05
can relate content knowledge from other disciplines to content knowledge in their discipline.	Keep	can relate content knowledge from other disciplines to content knowledge in their discipline.		can relate content knowledge from other disciplines to content knowledge in their discipline.	Split LO	know the ways that content knowledge from other disciplines is related to content knowledge in their discipline.	Foundational Disciplinary Knowledge	1.06
can use tools and databases to search the disciplinary literature.	Keep	can use tools and databases to search the disciplinary literature.	Keep	can use tools and databases to search the disciplinary literature.	Keep	use tools and databases to search the disciplinary literature.	Practical and Cognitive Research Skills	2.01
use literature search strategies that identify relevant prior research.	Keep	use literature search strategies that identify relevant prior research.	Keep	use literature search strategies that identify relevant prior research.	Keep	use literature search strategies that identify relevant prior research.	Practical and Cognitive Research Skills	2.02
use logical and critical thinking in evaluating information and knowledge and in conducting research.	Revise	use logical and critical thinking in evaluating information in research (e.g., designing, conducting, defending research)	Revise	use logical and critical thinking in evaluating information in research (e.g., designing, conducting, defending, and reviewing research	Keep	use logical and critical thinking in evaluating research	Practical & Cognitive Research Skills	2.03
connect diverse research ideas and approaches in novel ways.	Keep	connect diverse research ideas and approaches in novel ways.	Keep	connect diverse research ideas and approaches in novel ways.	Revise	connect diverse research ideas and approaches in novel and creative ways.	Practical & Cognitive Research Skills	2.04
consider alternative approaches and interpretations of research.	Keep	consider alternative approaches and interpretations of research.	Keep	consider alternative approaches and interpretations of research.	Keep	consider alternative approaches and interpretations of research.	Practical & Cognitive Research Skills	2.05
					Split LO	make connections between content knowledge in their discipline and content knowledge in other disciplines.	Practical & Cognitive Research Skills	2.06
identify gaps in existing knowledge to investigate.	Revise	identify gaps in existing knowledge or research results to investigate.	Keep	identify gaps in existing knowledge or research results to investigate.	Keep	identify gaps in existing knowledge or research results to investigate.	Practical & Cognitive Research Skills	2.07
set research goals.	Keep	set research goals.	Keep	set research goals.	Keep	set research goals.	Practical & Cognitive Research Skills	2.08
use disciplinary theories, frameworks and models in analyzing the results of research studies.	Keep	use disciplinary theories, frameworks and models in analyzing the results of research studies.	Keep	use disciplinary theories, frameworks and models in designing research studies.	Keep	use disciplinary theories, frameworks and models in designing research studies.	Practical & Cognitive Research Skills	2.09
can provide a logical rationale for their study designs.	Keep	can provide a logical rationale for their study designs.	Keep	can provide a logical rationale for their study designs.	Keep	can provide a logical rationale for their study designs.	Practical & Cognitive Research Skills	2.10
know assumptions and limitations in study designs.	Revise	know assumptions and limitations in study designs (e.g., reporting uncertainty/error).	Keep	know assumptions and limitations in study designs (e.g., reporting uncertainty/error).	Keep	know assumptions and limitations in study designs (e.g., reporting uncertainty/error).	Practical & Cognitive Research Skills	2.11
formulate hypotheses and research questions that can be systematically tested or investigated.	Keep	formulate hypotheses and research questions that can be systematically tested or investigated.	Keep	formulate hypotheses and research questions that can be systematically tested or investigated.	Keep	formulate hypotheses and research questions that can be systematically tested or investigated.	Practical & Cognitive Research Skills	2.12
know the research methods used in their discipline.	Merge							
select appropriate methods to investigate research questions and hypotheses in their discipline.	Merge						Practical & Cognitive Research Skills	2.13
can conduct the analytical and statistical methods used in their discipline.	Merge	know and select appropriate methods to investigate research questions in their discipline.	Revise	select appropriate methods to investigate research questions in the discipline.	Keep	select appropriate methods to investigate research questions		
follow standard protocols to document research data.	Merge		Keep		Split LO	follow standard procedures to collect and store data.	Practical & Cognitive Research Skills	2.14
follow standard protocols to securely store research data.	Merge	follow standard protocols to document and securely store research data.		follow standard protocols to document and securely store research data.	Split LO	follow ethical guidelines for working with research data.	Ethical and Responsible Research Practices	3.03
have the technical skills to conduct research in the discipline.	Keep	have the technical skills to conduct research in the discipline.	Revise	have up-to-date technical skills to conduct research in the discipline.	Keep	have up-to-date technical skills to conduct research in the discipline.	Practical & Cognitive Research Skills	2.15
	New LO	develop new data collection or analytical methods when needed to address novel research questions.	Keep	develop new data collection or analytical methods when needed to address novel research questions.	Keep	develop new data collection or analytical methods when needed	Practical & Cognitive Research Skills	2.16
use troubleshooting skills to address theoretical or technical problems in research.	Keep	use troubleshooting skills to address theoretical or technical problems in research.	Keep	use troubleshooting skills to address theoretical or technical problems in research.	Keep	use troubleshooting skills to address theoretical or technical problems in research.	Practical and Cognitive Research Skills	2.17
know the analytic and statistical methods used in their discipline.	Merge						Practical & Cognitive Research Skills	2.18
are able to select appropriate analytic and statistical methods to analyze data.	Merge	know and select the appropriate analytic and statistical methods used in their discipline.	Revise	select the appropriate analytic and statistical methods used in the discipline.	Revise	apply the appropriate analytic and statistical methods to analyze data.		
use disciplinary theories, frameworks and models in designing research studies.	Keep	use disciplinary theories, frameworks and models in designing research studies.	Keep	use disciplinary theories, frameworks and models in analyzing the results of research studies.	Keep	use disciplinary theories, frameworks and models in analyzing data	Practical and Cognitive Research Skills	2.19
can interpret the results of analyses.	Merge		Keep		Keep		Practical & Cognitive Research Skills	2.20
interpret statistical analyses of data.	Merge	can interpret the results of analyses of data (e.g., coding, mathematical and statistical calculations)	Keep	can interpret the results of analyses of data (e.g., coding, mathematical and statistical calculations)	Keep	interpret the results of data analyses (e.g., coding, mathematical, and statistical calculations)		
interpret or synthesize research results.	Keep	interpret or synthesize research results.	Keep	interpret or synthesize research results.	Keep	interpret or synthesize research findings	Practical & Cognitive Research Skills	2.21
					Split LO	propose new inferences and implications of research findings (their own and/or others')	Practical & Cognitive Research Skills	2.22
draw conclusions from research results.	Keep	draw conclusions from research results.	Keep	draw conclusions from research results.	Keep	draw conclusions from research findings	Practical & Cognitive Research Skills	2.23
refine existing and contribute new disciplinary theories, frameworks, and models.	Revise	refine existing and/or contribute new disciplinary theories, frameworks, and models.	Keep	refine existing and/or contribute new disciplinary theories, frameworks, and models.	Keep	refine existing and/or contribute new disciplinary theories, frameworks, and models based on research findings.	Practical and Cognitive Research Skills	2.24
follow research safety regulations.	Keep	follow research safety regulations.	Keep	follow research safety regulations.	Keep	follow research safety regulations.	Ethical and Responsible Research Practices	3.01
know and follow responsible research practices.	Revise	know and follow disciplinary data ownership/stewardship practices	Keep	know and follow disciplinary data ownership/stewardship practices	Keep	follow disciplinary data ownership and stewardship practices	Ethical and Responsible Research Practices	3.02

Original Learning Outcomes (LOs)	Decisions before Card Sort 1	LOs used in Card Sort 1	Decisions after Card Sort 1	LOs used in Card sort #2	Decisions after Card Sort 2	Final Version	Final Area	Final numbering
	New LO	know and follow guidelines for ethical treatment of research subjects (e.g., individuals, communities, animals, etc.)	Keep	know and follow guidelines for ethical treatment of research subjects (e.g., individuals, communities, animals, etc.)	Keep	follow guidelines for ethical treatment of research subjects (e.g., individuals, communities, animals, etc.)	Ethical and Responsible Research Practices	3.04
	New LO	know and follow guidelines for research rigor and reproducibility in your discipline	Keep	know and follow guidelines for research rigor and reproducibility in your discipline	Revise	follow guidelines for conducting research rigor and reproducibility in your discipline	Ethical and Responsible Research Practices	3.05
know and follow disciplinary norms and policies regarding credit for contributions to research (e.g., citing previous research, authorship order, acknowledging work).	Keep	know and follow disciplinary norms and policies regarding credit for contributions to research (e.g., citing previous research, authorship order, acknowledging work).	Keep	know and follow disciplinary norms and policies regarding credit for contributions to research (e.g., citing previous research, authorship order, acknowledging work).	Keep	follow disciplinary norms and policies regarding credit for contributions to research (e.g., citing previous research, authorship order, acknowledging work).	Ethical and Responsible Research Practices	3.06
are able to recognize legal and/or ethical issues that arise in research and take steps to resolve them.	Revise	recognize and minimize potential conflicts of interest in research	Keep	recognize and minimize potential conflicts of interest in research	Revise	recognize and minimize legal issues, ethical issues, and potential conflicts of interest in research	Ethical and Responsible Research Practices	3.07
	New LO	recognize instances of research misconduct and take steps to address them	Keep	recognize instances of research misconduct and take steps to address them	Keep	recognize instances of research misconduct and take steps to address them	Ethical and Responsible Research Practices	3.08
consider social and cultural factors in research.	Keep	consider social and cultural factors in research.	Revise	consider the role of social and cultural factors in research.	Keep	consider the role of social and cultural factors in research.	Ethical and Responsible Research Practices	3.09
consider the implications of research to individuals and society.	Keep	consider the implications of research to individuals and society.	Keep	consider the implications of research to individuals and society.	Keep	consider the implications of research to individuals and society.	Ethical and Responsible Research Practices	3.10
understand how system structures provide differential access to participation in research.	Keep	understand how system structures provide differential access to participation in research.	Keep	understand how system structures provide differential access to participation in research.	Revise	consider how system structures provide differential access to participation in research.	Ethical and Responsible Research Practices	3.11
act to increase access to research for all.	Keep	act to increase access to research for all.	Keep	act to increase access to research for all.	Keep	act to increase access to research for all.	Ethical and Responsible Research Practices	3.12
construct appropriate ways to present and visualize data.	Keep	construct appropriate ways to present and visualize data.	Keep	construct appropriate ways to present and visualize data.	Keep	construct appropriate ways to present and visualize data.	Research Communication Skills	4.01
use disciplinary conventions to communicate research (e.g., ideas, results, implications) orally (e.g., conference presentations, invited talks, research team meetings).	Revise	use disciplinary conventions to communicate research effectively (e.g., ideas, results, implications) orally (e.g., conference presentations, invited talks, research team meetings).	Keep	use disciplinary conventions to communicate research effectively (e.g., ideas, results, implications) orally (e.g., conference presentations, invited talks, research team meetings).	Keep	use disciplinary conventions to communicate research (e.g., ideas, results, implications) orally (e.g., conference presentations, invited talks, research team meetings).	Research Communication Skills	4.02
use disciplinary conventions to communicate research (e.g., ideas, results, implications) in writing (e.g., research articles, grant proposals, policy briefs).	Revise	use disciplinary conventions to communicate research effectively (e.g., ideas, results, implications) in writing (e.g., research articles, grant proposals, policy briefs).	Keep	use disciplinary conventions to communicate research effectively (e.g., ideas, results, implications) in writing (e.g., research articles, grant proposals, policy briefs).	Keep	use disciplinary conventions to communicate research (e.g., ideas, results, implications) in writing (e.g., research articles, grant proposals, policy briefs).	Research Communication Skills	4.03
are able to translate research findings into policies and practices.	Revise	are able to translate research findings into policies, practices, and daily life.	Keep	are able to translate research findings into policies, practices, and daily life.	Keep	are able to translate research findings into policies, practices	Research Communication Skills	4.04
can translate research (e.g., ideas, results, implications) to audiences outside of their research discipline (e.g., to scholars in other disciplines, non-research, or general audiences).	Revise	can translate research (e.g., ideas, results, implications) and engage with audiences outside of their research discipline (e.g., to scholars in other disciplines, non-research, or general audiences).	Keep	can translate research (e.g., ideas, results, implications) and engage with audiences outside of their research discipline (e.g., to scholars in other disciplines, non-research, or general audiences).	Keep	translate research (e.g., ideas, results, implications) and engage with audiences outside of their research discipline (e.g., to scholars in other disciplines, non-research, or general audiences).	Research Communication Skills	4.05
promote and advocate for research through interactions with public stakeholders.	Revise	promote and advocate for research within the institution, the discipline, and through interactions with public stakeholders.	Keep	promote and advocate for research within the institution, the discipline, and through interactions with public stakeholders.	Revise	promote and advocate for research through communications to various audiences (e.g., institution, disciplines, public stakeholders)	Research Communication Skil	4.06
understand and conduct themselves in accordance with the cultural and social norms of professionals in the discipline.	Keep	understand and conduct themselves in accordance with the cultural and social norms of professionals in the discipline.	Keep	understand and conduct themselves in accordance with the cultural and social norms of professionals in the discipline.	Keep	understand and conduct themselves in accordance with the cultural and social norms of professionals in the discipline.	Interpersonal Research Skills	5.01
express respect for others' differences.	Keep	express respect for others' differences.	Keep	express respect for others' differences.	Keep	express respect for others' differences.	Interpersonal Research Skills	5.02
use effective interpersonal communication practices with research colleagues.	Revise	use appropriate and effective interpersonal communication practices with research colleagues.	Keep	use appropriate and effective interpersonal communication practices with research colleagues.	Keep	use appropriate and effective interpersonal communication practices with research colleagues.	Interpersonal Research Skills	5.03
are able to manage conflicts with research colleagues.	Revise	are able to manage difficult conversations and conflicts with research colleagues.	Keep	are able to manage difficult conversations and conflicts with research colleagues.	Keep	are able to manage difficult conversations and conflicts with research colleagues.	Interpersonal Research Skills	5.04
work effectively with others on collaborative teams.	Revise	work effectively with others on collaborative and/or interdisciplinary teams.	Keep	work effectively with others on collaborative and/or interdisciplinary teams.	Keep	work effectively with others on collaborative and/or interdisciplinary teams.	Interpersonal Research Skills	5.05
consider and include multiple perspectives in decision making.	Keep	consider and include multiple perspectives in decision making.	Keep	consider and include multiple perspectives in decision making.	Keep	consider and include multiple perspectives in decision making.	Interpersonal Research Skills	5.06
make meaningful contributions to collaborative research projects.	Keep	make meaningful contributions to collaborative research projects.	Keep	make meaningful contributions to collaborative research projects.	Keep	make meaningful contributions to collaborative research projects.	Interpersonal Research Skills	5.07
provide critical and constructive feedback on research to colleagues.	Keep	provide critical and constructive feedback on research to colleagues.	Keep	provide critical and constructive feedback on research to colleagues.	Keep	provide critical and constructive feedback on research to colleagues.	Interpersonal Research Skills	5.08
accept, interpret,and modify their research based on constructive criticism and feedback from colleagues.	Keep	accept, interpret,and modify their research based on constructive criticism and feedback from colleagues.	Keep	accept, interpret,and modify their research based on constructive criticism and feedback from colleagues.	Keep	accept, interpret,and modify their research based on constructive criticism and feedback from colleagues.	Interpersonal Research Skills	5.09
are able to network with other research professionals.	Keep	are able to network with other research professionals.	Keep	are able to network with other research professionals.	Keep	are able to network with other research professionals.	Interpersonal Research Skills	5.10
	New LO	self advocate when working with mentors to set research goals and secure the guidance and resources needed to achieve those goals.	Revise	self advocate and take responsibility when working with mentors to set research goals and secure the guidance and resources needed to achieve those goals.	Keep	proactively -set their research goals and secure the guidance and resources needed to achieve those goals.	Researcher Self-Beliefs & Attitudes	6.01
persevere when problems or challenges arise in research.	Revise	perservere when problems or challenges arise in research (e.g., unexpected, ambiguous or uncertain results, failed projects)	Keep	perservere when problems or challenges arise in research (e.g., unexpected, ambiguous or uncertain results, failed projects)	Keep	perservere when problems or challenges arise in research (e.g., unexpected, ambiguous or uncertain results, failed projects)	Researcher Self-Beliefs & Attitudes	6.02
are able to recognize and manage their feelings and behaviors in the research environment.	Keep	are able to recognize and manage their feelings and behaviors in the research environment.	Keep	are able to recognize and manage their feelings and behaviors in the research environment.	Keep	recognize and manage their feelings and behaviors in the research environment.	Researcher Self-Beliefs & Attitudes	6.03
are able to accurately self-assess their strengths and weaknesses.	Keep	are able to accurately self-assess their strengths and weaknesses.	Keep	are able to accurately self-assess their strengths and weaknesses.	Keep	accurately self-assess their research strengths and weaknesses.	Researcher Self-Beliefs & Attitudes	6.04
demonstrate curiosity in exploring and conducting research.	Keep	demonstrate curiosity in exploring and conducting research.	Revise	express curiosity in exploring and conducting research.	Keep	express curiosity in exploring and conducting research.	Researcher Self-Beliefs & Attitudes	6.05
work at an appropriate level of independence.	Keep	work at an appropriate level of independence.	Keep	work at an appropriate level of independence.	Keep	work at an appropriate level of independence.	Researcher Self-Beliefs and Attitudes	6.06
are able to meet research project milestones in a timely manner.	Keep	are able to meet research project milestones in a timely manner.	Revise	are able to manage time and meet research project milestones.	Revise	manage time to meet individual project milestones	Researcher Self-Beliefs & Attitudes	6.07
develop confidence in their capability to successfully conduct research.	Keep	develop confidence in their capability to successfully conduct research.	Keep	develop confidence in their capability to successfully conduct research.	Keep	develop confidence in their capability to successfully conduct research.	Researcher Self-Beliefs & Attitudes	6.08
identify themselves as a researcher in their discipline.	Revise	identify themselves as a researcher or expert in their discipline.	Keep	identify themselves as a researcher or expert in their discipline.	Keep	identify themselves as a researcher or expert in their discipline.	Researcher Self-Beliefs & Attitudes	6.09

Original Learning Outcomes (LOs)	Decisions before Card Sort 1	LOs used in Card Sort 1	Decisions after Card Sort 1	LOs used in Card sort #2	Decisions after Card Sort 2	Final Version	Final Area	Final numbering
engage in practices that support work-life balance.	Revise	engage in practices that support work-life balance (e.g., time management, pursuing interests beyond research)	Keep	engage in practices that support work-life balance (e.g., time management, pursuing interests beyond research)	Keep	engage in practices that support work-life balance (e.g., time management, pursuing interests beyond research)	Researcher Self-Beliefs & Attitudes	6.10
are aware of career pathways related to their research training.	Keep	are aware of career pathways related to their research training.	Keep	are aware of career pathways related to their research training.	Keep	are aware of career pathways related to their research training.	Knowledge & Skills to Pursue a Research or Research-Related Career	7.01
identify and clarify a long-term strategic vision for research.	Keep	identify and clarify a long-term strategic vision for research.	Keep	identify and clarify a long-term strategic vision for research.	Split LO	identify and clarify a long-term strategic vision for their research career.	Knowledge & Skills to Pursue a Research or Research-Related Career	7.02
can translate and apply research skills and knowledge across career pathways.	Keep	can translate and apply research skills and knowledge across career pathways.	Keep	can translate and apply research skills and knowledge across career pathways.	Keep	translate and apply their research skills and knowledge across career pathways.	Knowledge & Skills to Pursue a Research or Research-Related Career	7.03
are prepared to pursue research career pathways.	Keep	are prepared to pursue research career pathways.	Keep	are prepared to pursue research career pathways.	Keep	are prepared to pursue research career pathways.	Knowledge & Skills to Pursue a Research or Research-Related Career	7.04
					Split LO	identify and clarify a long-term strategic vision for a program of research.	Knowledge & Skills to Administer and Manage Research Projects and Teams	8.01
identify opportunities and make decisions about the research to be done.	Keep	identify opportunities and make decisions about the research to be done.	Keep	identify opportunities and make decisions about the research to be done.	Revise	identify opportunities and make decisions about the research to be done	Knowledge & Skills to Administer and Manage Research Projects and Teams	8.02
mentor others learning to do research.	Keep	mentor others learning to do research.	Revise	mentor other developing researchers using best practices in mentoring.	Keep	mentor other developing researchers using best practices in mentoring.	Knolwedge & Skills to Administer and Manage Research Projects and Teams	8.03
know how research is funded in the discipline.	Keep	know how research is funded in the discipline.	Keep	know how research is funded in the discipline.	Revise	know how to identify and secure funding (e.g. investments, grants) to support research in their discipline.	Knolwedge & Skills to Administer and Manage Research Projects and Teams	8.04
can estimate the funds needed to conduct research.	Keep	can estimate the funds needed to conduct research.	Keep	can estimate the funds needed to conduct research.	Keep	can estimate and secure the funds needed to conduct research.	Knolwedge & Skills to Administer and Manage Research Projects and Teams	8.05
can track research expenditures.	Keep	can track research expenditures.	Keep	responsibly spend and track research expenditures.	Keep	track research expenditures.	Knolwedge & Skills to Administer and Manage Research Projects and Teams	8.06
have the administrative skills to manage research projects and personnel.	Revise	have the administrative skills to manage research projects, personnel, and support staff.	Keep	have the administrative skills to manage research projects, personnel, and support staff.	Keep	have the administrative skills to manage research projects and/or heterogeneous research teams.	Knowledge & Skills to Administer and Manage Research Projects and Teams	8.07
develop attitudes about research that support success in research.	Keep	develop attitudes about research that support success in research.	Keep	develop attitudes about research that support success in research.	Delete	--DELETED--		
are able to manage research teams.	Revise	are able to manage heterogenous research teams.	Keep	are able to manage heterogenous research teams.	Delete	--DELETED--		
can secure funding to conduct research.	Keep	can secure funding to conduct research.	Keep	can secure funding to conduct research.	Delete	--DELETED--		
use disciplinary conventions to create research presentations.	Delete	--DELETED--		--DELETED--				
Total Count of LOS								
79		78		78		79		
