# Supplementary material for: The comprehensive researcher development framework (CRDF): Core learning outcomes for research training
Source: PLoS One. 2025 Sep 15;20(9):e0332587. doi: 10.1371/journal.pone.0332587 (PMC12435680; doi:10.1371/journal.pone.0332587)
Supplement: S5 Appendix — (PDF) [file pone.0332587.s006.pdf]

S5. Dissemination Contacts for National Survey

Contact Name/Group	Description
Author contacts	Individuals within each author's network
Center for the Improvement of Mentored Experiences in Research (CIMER)	Trained Facilitators of Entering Research Curriculum
University of Wisconsin - Madison	• Department chairs<br>• graduate training program directors and coordinators<br>• Research Education Working group<br>• Mentoring Working group<br>• UW-Madison Postdocs
Grant-funded training programs across the nation	• NSF Research Experiences for Undergraduates (REU) Program directors<br>• NIH Building Infrastructure leading to Diversity (BUILD) Program directors
Regional and National Professional Groups	• Big 10 Postdoc Office<br>• National Research Mentoring Network (NRMN) Online Community<br>• Council of Undergraduate Research<br>• Council of Graduate Schools Humanities Coalition Advisory Committee<br>• National Postdoc Association<br>• Undergraduate Research Center Directors
