# Supplementary material for: The comprehensive researcher development framework (CRDF): Core learning outcomes for research training
Source: PLoS One. 2025 Sep 15;20(9):e0332587. doi: 10.1371/journal.pone.0332587 (PMC12435680; doi:10.1371/journal.pone.0332587)
Supplement: S6 Appendix — (PDF) [file pone.0332587.s007.pdf]

S6a Open Card Sorting Data

Card no	Card name	Group 1	Group 2	Group 3	Group 4	Group 5	Group 6	Group 7	Group 8	Group 9
1	accept, interpret,and modify their research based on constructive criticism and feedback from colleagues.	Research Content Knowledge	Communication/ Feedback	Professional Communication	Self-Efficacy	Grit/ Self-Regulation	Professional Skills	Mentorship soft skills management	Self Management	Interpersonal & research Management
2	recognize and minimize potential conflicts of interest in research	Research ethics	RCR	Guidelines/ Professional Ethics	Ethics	Ethical Practices	Ethics	Ethics undergrad first, postdoc last	Resonsible Conduct of research / Research Integrity	RCR
3	have the technical skills to conduct research in the discipline.	Research Tech Skills	Foundational knowledge	Technical Competency	Foundational Research Skills	Technical Research Skills	Professional Skills	Analysis/technical skills	Disciplinary Knowledge	Research process - conducting research & synthesizing new knowledge
4	know assumptions and limitations in study designs (e.g., reporting uncertainty/error).	Research Interpretation	Planning/ hypothesis	Expertise - Mid Stage	Experimental Design & Analysis	Technical Research Skills	How to design + Execute An Experiment or project	Analysis/technical skills	Basic Research Skills	Research process - conducting research & synthesizing new knowledge
5	use troubleshooting skills to address theoretical or technical problems in research.	Research Tech Skills	Affective	Technical Competency	Foundational Research Skills	Technical Research Skills	Technical	Analysis/technical skills	Basic Research Skills	Research process - conducting research & synthesizing new knowledge
6	identify and clarify a long-term strategic vision for research.	Administration	Synthesis	Expertise - Late Stage	Research Management & Implementation	Research Skills Related to Overall Discipline/ Research Vision	Research/Career Independence	Career Development	Leadership	Research process - conducting research & synthesizing new knowledge
7	know and follow disciplinary norms and policies regarding credit for contributions to research (e.g., citing previous research, authorship order, acknowledging work).	Research ethics	RCR	Guidelines/ Professional Ethics	Communication & Sharing to Society	Ethical Practices	Ethics	Ethics undergrad first, postdoc last	Resonsible Conduct of research / Research Integrity	RCR
8	develop confidence in their capability to successfully conduct research.	Self-efficacy	Affective	Agency, Grit, Ownership, Resilience	Self-Efficacy	Grit/ Self-Regulation	Attitude/Personal Development	Personal Development	Self Management	Self-efficacy & Research identity
9	know the fundamental content in their discipline.	Research Content Knowledge	Foundational knowledge	Expertise - Early Stage	Foundational Research Skills	Disciplinary Knowledge	Discipline Knowledge	Knowledge/ breadth	Disciplinary Knowledge	Research process - conducting research & synthesizing new knowledge
10	are aware of career pathways related to their research training.	Career exploration	Career	Technical Competency	Research Management & Implementation	Research Skills Related to Overall Discipline/ Research Vision	Personal/Career Knowledge Development	Career Development	Career Pathway	Self-efficacy & Research identity
11	know and select appropriate methods to investigate research questions in their discipline.	Research Content Knowledge	Planning/ hypothesis	Technical Competency	Experimental Design & Analysis	Technical Research Skills	How to design + Execute An Experiment or project	Analysis/technical skills	Disciplinary Knowledge	Research process - conducting research & synthesizing new knowledge
12	mentor others learning to do research.	Social aspects/ lab culture	Mentoring	People (Interpersonal)	Research Management & Implementation	Professional Collaboration	Mentorship	Mentorship soft skills management	Leadership	Interpersonal & research Management
13	use disciplinary conventions to communicate research effectively (e.g., ideas, results, implications) orally (e.g., conference presentations, invited talks, research team meetings).	Research communication	Communication/ Feedback	Technical Competency	Communication & Sharing to Society	Professional Collaboration	Research Communication	Communication and collaboration	Scientific Communication	Communication
14	draw conclusions from research results.	Research Interpretation	Interpretation	Expertise - Mid Stage	Experimental Design & Analysis	Technical Research Skills	How to design + Execute An Experiment or project	Critical thinking	Basic Research Skills	Research process - conducting research & synthesizing new knowledge
15	are prepared to pursue research career pathways.	Career exploration	Career	Agency, Grit, Ownership, Resilience	Research Management & Implementation	Research Skills Related to Overall Discipline/ Research Vision	Personal/Career Knowledge Development	Career Development	Career Pathway	Self-efficacy & Research identity
16	can use tools and databases to search the disciplinary literature.	Research Content Knowledge	Foundational knowledge	Technical Competency	Foundational Research Skills	Disciplinary Knowledge	Technical	Analysis/technical skills	Basic Research Skills	Research process - conducting research & synthesizing new knowledge
17	can estimate the funds needed to conduct research.	Administration	Money/ Resources - Rsrch Admin	Finance Resources	Research Management & Implementation	Management/Administrative	Funding	Money	Lab Management	Funding
18	construct appropriate ways to present and visualize data.	Research communication	Interpretation	Technical Competency	Communication & Sharing to Society	Technical Research Skills	Technical	Communication and collaboration	Scientific Communication	Communication
19	are able to manage heterogenous research teams.	Administration	Teams/ Group Networking	People (Interpersonal)	Collaborative Research	Promoting Equality in Research	Management	Mentorship soft skills management	Leadership	Interpersonal & research Management
20	know how research is funded in the discipline.	Administration	Money/ Resources - Rsrch Admin	Finance Resources	Research Management & Implementation	Management/Administrative	Funding	Money	Lab Management	Funding
21	make meaningful contributions to collaborative research projects.	Comm w/ others	Teams/ Group Networking	Expertise - Mid Stage	Collaborative Research	Professional Collaboration	Collaboration	Communication and collaboration	Collaborative Teams	Self-efficacy & Research identity
22	know and select the appropriate analytic and statistical methods used in their discipline.	Research Tech Skills	Planning/ hypothesis	Technical Competency	Experimental Design & Analysis	Disciplinary Knowledge	Technical	Analysis/technical skills	Basic Research Skills	Research process - conducting research & synthesizing new knowledge
23	use disciplinary theories, frameworks and models in analyzing the results of research studies.	Research Interpretation	Interpretation	Expertise - Late Stage	Experimental Design & Analysis	Disciplinary Knowledge	Technical	Analysis/technical skills	Basic Research Skills	Research process - conducting research & synthesizing new knowledge
24	use logical and critical thinking in evaluating information in research (e.g., designing, conducting, defending research)	Research Tech Skills	Interpretation	Expertise - Mid Stage	Experimental Design & Analysis	Technical Research Skills	Professional Skills	Critical thinking	Basic Research Skills	Research process - conducting research & synthesizing new knowledge
25	consider alternative approaches and interpretations of research.	Research Interpretation	Interpretation	Expertise - Early Stage	Collaborative Research	Technical Research Skills	How to design + Execute An Experiment or project	Knowledge/ breadth	Basic Research Skills	Research process - conducting research & synthesizing new knowledge
26	can translate and apply research skills and knowledge across career pathways.	Career exploration	Career	Ethical / Professional Behavior	Communication & Sharing to Society	Research Skills Related to Overall Discipline/ Research Vision	Research/Career Independence	Career Development	Career Pathway	Research process - conducting research & synthesizing new knowledge
27	identify opportunities and make decisions about the research to be done.	Administration	Money/ Resources - Rsrch Admin	Expertise - Late Stage	Research Management & Implementation	Research Skills Related to Overall Discipline/ Research Vision	Research/Career Independence	Knowledge/ breadth	Leadership	Research process - conducting research & synthesizing new knowledge
28	promote and advocate for research within the institution, the discipline, and through interactions with public stakeholders.	Administration	Translational/ Public Science	Professional Communication	Communication & Sharing to Society	Ethical Practices	Research Communication	Advocacy	Scientific Communication to non-scientific community	Communication
29	can relate content knowledge from other disciplines to content knowledge in their discipline.	Research Interpretation	Foundational knowledge	Expertise - Early Stage	Collaborative Research	Disciplinary Knowledge	Discipline Knowledge	Knowledge/ breadth	Research independence/ novel research questions	Research process - conducting research & synthesizing new knowledge
30	set research goals.	Administration	Planning/ hypothesis	Agency, Grit, Ownership, Resilience	Experimental Design & Analysis	Research Skills Related to Overall Discipline/ Research Vision	How to design + Execute An Experiment or project	Analysis/technical skills	Basic Research Skills	Research process - conducting research & synthesizing new knowledge
31	can track research expenditures.	Administration	Money/ Resources - Rsrch Admin	Finance Resources	Research Management & Implementation	Management/Administrative	Funding	Money	Lab Management	Funding

Card no	Card name	Group 1	Group 2	Group 3	Group 4	Group 5	Group 6	Group 7	Group 8	Group 9
32	consider and include multiple perspectives in decision making.	Research Interpretation	Access/Inclusion	Ethical / Professional Behavior	Collaborative Research	Promoting Equality in Research	Collaboration	Advocacy	Leadership	RCR
33	understand how system structures provide differential access to participation in research.	Research ethics	Access/Inclusion	People (Interpersonal)	Ethics	Promoting Equality in Research	Inclusion	Ethics undergrad first, postdoc last	Broader Impacts	RCR
34	engage in practices that support work-life balance (e.g., time management, pursuing interests beyond research)	Social aspects/ lab culture	Career	People (Interpersonal)	Ethics	Grit/ Self-Regulation	Attitude/Personal Development	Career Development	Leadership	Self-efficacy & Research identity
35	follow standard protocols to document and securely store research data.	Research Tech Skills	RCR	Guidelines/ Professional Ethics	Ethics	Ethical Practices	Ethics	Analysis/technical skills	Resonsible Conduct of research / Research Integrity	RCR
36	use appropriate and effective interpersonal communication practices with research colleagues.	Comm w/ others	Teams/ Group Networking	Professional Communication	Collaborative Research	Professional Collaboration	Collaboration	Communication and collaboration	Collaborative Teams	Interpersonal & research Management
37	understand and conduct themselves in accordance with the cultural and social norms of professionals in the discipline.	Social aspects/ lab culture	RCR	People (Interpersonal)	Ethics	Professional Collaboration	Professional Skills	Mentorship soft skills management	Broader Impacts	RCR
38	perservere when problems or challenges arise in research (e.g., unexpected, ambiguous or uncertain results, failed projects)	Self-efficacy	Affective	Agency, Grit, Ownership, Resilience	Self-Efficacy	Grit/ Self-Regulation	Professional Skills	Personal Development	Self Management	Self-efficacy & Research identity
39	refine existing and/or contribute new disciplinary theories, frameworks, and models.	Research Interpretation	Synthesis	Expertise - Late Stage	Experimental Design & Analysis	Disciplinary Knowledge	Research/Career Independence	Critical thinking	Research independence/ novel research questions	Research process - conducting research & synthesizing new knowledge
40	ground hypotheses and research questions in established disciplinary knowledge, theories, or frameworks.	Research Content Knowledge	Planning/ hypothesis	Expertise - Early Stage	Experimental Design & Analysis	Disciplinary Knowledge	How to design + Execute An Experiment or project	Knowledge/ breadth	Basic Research Skills	Research process - conducting research & synthesizing new knowledge
41	are able to accurately self-assess their strengths and weaknesses.	Self-efficacy	Affective	Agency, Grit, Ownership, Resilience	Self-Efficacy	Grit/ Self-Regulation	Attitude/Personal Development	Personal Development	Self Management	Self-efficacy & Research identity
42	develop attitudes about research that support success in research.	Self-efficacy	Affective	Agency, Grit, Ownership, Resilience	Ethics	Research Skills Related to Overall Discipline/ Research Vision	Attitude/Personal Development	Personal Development	Leadership	Self-efficacy & Research identity
43	connect diverse research ideas and approaches in novel ways.	Research Interpretation	Synthesis	Expertise - Late Stage	Communication & Sharing to Society	Research Skills Related to Overall Discipline/ Research Vision	How to design + Execute An Experiment or project	Knowledge/ breadth	Research independence/ novel research questions	Research process - conducting research & synthesizing new knowledge
44	consider the implications of research to individuals and society.	Research ethics	RCR	Guidelines/ Professional Ethics	Ethics	Public Communication	Public/ Broad Communication	Ethics undergrad first, postdoc last	Broader Impacts	RCR
45	know the history of knowledge generation in their discipline.	Research Content Knowledge	Foundational knowledge	Expertise - Early Stage	Foundational Research Skills	Disciplinary Knowledge	Discipline Knowledge	Knowledge/ breadth	Disciplinary Knowledge	Research process - conducting research & synthesizing new knowledge
46	know the processes by which new knowledge is generated and evaluated in their discipline.	Research Content Knowledge	Foundational knowledge	Expertise - Early Stage	Foundational Research Skills	Disciplinary Knowledge	Discipline Knowledge	Knowledge/ breadth	Disciplinary Knowledge	Research process - conducting research & synthesizing new knowledge
47	recognize instances of research misconduct and take steps to address them	Research ethics	RCR	Guidelines/ Professional Ethics	Ethics	Ethical Practices	Ethics	Ethics undergrad first, postdoc last	Resonsible Conduct of research / Research Integrity	RCR
48	have the administrative skills to manage research projects, personnel, and support staff.	Administration	Money/ Resources - Rsrch Admin	Ethical / Professional Behavior	Research Management & Implementation	Management/Administrative	Management	Money	Lab Management	Interpersonal & research Management
49	are able to recognize and manage their feelings and behaviors in the research environment.	Social aspects/ lab culture	Affective	Ethical / Professional Behavior	Ethics	Grit/ Self-Regulation	Attitude/Personal Development	Mentorship soft skills management	Self Management	Self-efficacy & Research identity
50	interpret or synthesize research results.	Research Interpretation	Interpretation	Expertise - Late Stage	Foundational Research Skills	Technical Research Skills	How to design + Execute An Experiment or project	Critical thinking	Basic Research Skills	Research process - conducting research & synthesizing new knowledge
51	use literature search strategies that identify relevant prior research.	Research Content Knowledge	Foundational knowledge	Expertise - Early Stage	Experimental Design & Analysis	Disciplinary Knowledge	Discipline Knowledge	Analysis/technical skills	Basic Research Skills	Research process - conducting research & synthesizing new knowledge
52	identify themselves as a researcher or expert in their discipline.	Self-efficacy	Affective	Ethical / Professional Behavior	Self-Efficacy	Professional Collaboration	Attitude/Personal Development	Personal Development	Self Management	Self-efficacy & Research identity
53	work effectively with others on collaborative and/or interdisciplinary teams.	Comm w/ others	Teams/ Group Networking	People (Interpersonal)	Collaborative Research	Professional Collaboration	Collaboration	Communication and collaboration	Collaborative Teams	Interpersonal & research Management
54	can secure funding to conduct research.	Administration	Money/ Resources - Rsrch Admin	Finance Resources	Research Management & Implementation	Management/Administrative	Funding	Money	Lab Management	Funding
55	can interpret the results of analyses of data (e.g., coding, mathematical and statistical calculations)	Research Interpretation	Interpretation	Expertise - Mid Stage	Foundational Research Skills	Technical Research Skills	Technical	Analysis/technical skills	Basic Research Skills	Research process - conducting research & synthesizing new knowledge
56	work at an appropriate level of independence.	Self-efficacy	Affective	Expertise - Mid Stage	Research Management & Implementation	Research Skills Related to Overall Discipline/ Research Vision	Attitude/Personal Development	Personal Development	Self Management	Self-efficacy & Research identity
57	consider social and cultural factors in research.	Social aspects/ lab culture	Access/Inclusion	People (Interpersonal)	Ethics	Promoting Equality in Research	Inclusion	Ethics undergrad first, postdoc last	Broader Impacts	RCR
58	know and follow guidelines for ethical treatment of research subjects (e.g., individuals, communities, animals, etc.)	Research ethics	RCR	Guidelines/ Professional Ethics	Ethics	Ethical Practices	Ethics	Ethics undergrad first, postdoc last	Resonsible Conduct of research / Research Integrity	RCR
59	can provide a logical rationale for their study designs.	Research Interpretation	Planning/ hypothesis	Expertise - Early Stage	Experimental Design & Analysis	Research Skills Related to Overall Discipline/ Research Vision	How to design + Execute An Experiment or project	Critical thinking	Basic Research Skills	Research process - conducting research & synthesizing new knowledge
60	are able to translate research findings into policies, practices, and daily life.	Research communication	Translational/ Public Science	Professional Communication	Communication & Sharing to Society	Public Communication	Public/ Broad Communication	Communication and collaboration	Scientific Communication to non-scientific community	Communication
61	can translate research (e.g., ideas, results, implications) and engage with audiences outside of their research discipline (e.g., to scholars in other disciplines, non-research, or general audiences).	Research communication	Translational/ Public Science	Professional Communication	Communication & Sharing to Society	Public Communication	Public/ Broad Communication	Communication and collaboration	Scientific Communication to non-scientific community	Communication
62	recognize the inferences and implications of research findings on and beyond the discipline.	Research communication	Translational/ Public Science	Expertise - Late Stage	Communication & Sharing to Society	Research Skills Related to Overall Discipline/ Research Vision	Public/ Broad Communication	Critical thinking	Broader Impacts	Research process - conducting research & synthesizing new knowledge
63	express respect for others' differences.	Research ethics	Access/Inclusion	People (Interpersonal)	Ethics	Promoting Equality in Research	Collaboration	Ethics undergrad first, postdoc last	Leadership	Interpersonal & research Management
64	develop new data collection or analytical methods when needed to address novel research questions.	Research Tech Skills	Interpretation	Expertise - Late Stage	Experimental Design & Analysis	Technical Research Skills	Technical	Critical thinking	Research independence/ novel research questions	Research process - conducting research & synthesizing new knowledge
65	identify gaps in existing knowledge or research results to investigate.	Research Content Knowledge	Foundational knowledge	Expertise - Early Stage	Experimental Design & Analysis	Disciplinary Knowledge	Discipline Knowledge	Knowledge/ breadth	Research independence/ novel research questions	Research process - conducting research & synthesizing new knowledge

Card no	Card name	Group 1	Group 2	Group 3	Group 4	Group 5	Group 6	Group 7	Group 8	Group 9
66	demonstrate curiosity in exploring and conducting research.	Research Content Knowledge	Affective	Agency, Grit, Ownership, Resilience	Self-Efficacy	Research Skills Related to Overall Discipline/ Research Vision	Research/Career Independence	Personal Development	Self Management	Self-efficacy & Research identity
67	use disciplinary theories, frameworks and models in designing research studies.	Research Interpretation	Planning/ hypothesis	Technical Competency	Experimental Design & Analysis	Technical Research Skills	How to design + Execute An Experiment or project	Critical thinking	Disciplinary Knowledge	Research process - conducting research & synthesizing new knowledge
68	know and follow guidelines for research rigor and reproducibility in your discipline	Research ethics	RCR	Guidelines/ Professional Ethics	Ethics	Ethical Practices	Ethics	Ethics undergrad first, postdoc last	Resonsible Conduct of research / Research Integrity	RCR
69	are able to network with other research professionals.	Comm w/ others	Teams/ Group Networking	People (Interpersonal)	Communication & Sharing to Society	Professional Collaboration	Professional Skills	Communication and collaboration	Scientific Communication	Interpersonal & research Management
70	know and follow disciplinary data ownership/stewardship practices	Research Tech Skills	RCR	Ethical / Professional Behavior	Ethics	Ethical Practices	Ethics	Ethics undergrad first, postdoc last	Resonsible Conduct of research / Research Integrity	RCR
71	follow research safety regulations.	Research Tech Skills	RCR	Guidelines/ Professional Ethics	Ethics	Ethical Practices	Safety	Ethics undergrad first, postdoc last	Resonsible Conduct of research / Research Integrity	Research process - conducting research & synthesizing new knowledge
72	use disciplinary conventions to communicate research effectively (e.g., ideas, results, implications) in writing (e.g., research articles, grant proposals, policy briefs).	Research communication	Translational/ Public Science	Technical Competency	Communication & Sharing to Society	Professional Collaboration	Research Communication	Communication and collaboration	Scientific Communication	Communication
73	formulate hypotheses and research questions that can be systematically tested or investigated.	Research Tech Skills	Planning/ hypothesis	Expertise - Mid Stage	Experimental Design & Analysis	Technical Research Skills	How to design + Execute An Experiment or project	Critical thinking	Basic Research Skills	Research process - conducting research & synthesizing new knowledge
74	provide critical and constructive feedback on research to colleagues.	Social aspects/ lab culture	Communication/ Feedback	Professional Communication	Collaborative Research	Professional Collaboration	Collaboration	Mentorship soft skills management	Leadership	Interpersonal & research Management
75	self advocate when working with mentors to set research goals and secure the guidance and resources needed to achieve those goals.	Self-efficacy	Affective	Agency, Grit, Ownership, Resilience	Collaborative Research	Research Skills Related to Overall Discipline/ Research Vision	Professional Skills	Personal Development	Self Management	Self-efficacy & Research identity
76	are able to manage difficult conversations and conflicts with research colleagues.	Comm w/ others	Teams/ Group Networking	People (Interpersonal)	Collaborative Research	Grit/ Self-Regulation	Collaboration	Mentorship soft skills management	Leadership	Interpersonal & research Management
77	are able to meet research project milestones in a timely manner.	Administration	Foundational knowledge	Ethical / Professional Behavior	Research Management & Implementation	Research Skills Related to Overall Discipline/ Research Vision	Professional Skills	Money	Leadership	Research process - conducting research & synthesizing new knowledge
78	act to increase access to research for all.	Social aspects/ lab culture	Access/Inclusion	People (Interpersonal)	Communication & Sharing to Society	Promoting Equality in Research	Inclusion	Advocacy	Broader Impacts	RCR

S6b. Organization of Original Categories Suggested by Each Group into Common, Standardized Categories

Group	Original category	Standardized category
Group 1	Research ethics	Ethical and Responsible Research Practices
Group 1	Social aspects/ lab culture	Interpersonal Skills in Research
Group 1	Administration	Research Administration and Management
Group 1	Career exploration	Research Career Development
Group 1	Research communication	Research Communication
Group 1	Research Interpretation	Thinking about Research
Group 1	Research Content Knowledge	Foundational Disciplinary Knowledge
Group 1	Research Tech Skills	Doing Research
Group 1	Comm w/ others	Interpersonal Skills in Research
Group 1	Self-efficacy	Personal Development as a Researcher
Group 2	Access/Inclusion	Interpersonal Skills in Research
Group 2	Translational/ Public Science	Research Communication
Group 2	Communication/ Feedback	Interpersonal Skills in Research
Group 2	Teams/ Group Networking	Interpersonal Skills in Research
Group 2	Career	Research Career Development
Group 2	Planning/ hypothesis	Doing Research
Group 2	Foundational knowledge	Foundational Disciplinary Knowledge
Group 2	Money/ Resources - Rsrch Admin	Research Administration and Management
Group 2	Mentoring	Interpersonal Skills in Research
Group 2	Affective	Personal Development as a Researcher
Group 2	Synthesis	Thinking about Research
Group 2	Interpretation	Thinking about Research
Group 2	RCR	Ethical and Responsible Research Practices
Group 3	Technical Competency	Doing Research
Group 3	Finance Resources	Research Administration and Management
Group 3	People (Interpersonal)	Interpersonal Skills in Research
Group 3	Professional Communication	Research Communication
Group 3	Expertise - Late Stage	Thinking about Research
Group 3	Expertise - Mid Stage	Doing Research

Group	Original category	Standardized category
Group 3	Expertise - Early Stage	Foundational Disciplinary Knowledge
Group 3	Ethical / Professional Behavior	Ethical and Responsible Research Practices
Group 3	Guidelines/ Professional Ethics	Ethical and Responsible Research Practices
Group 3	Agency, Grit, Ownership, Resilience	Personal Development as a Researcher
Group 4	Experimental Design & Analysis	Thinking about Research
Group 4	Research Management & Implementation	Research Administration and Management
Group 4	Ethics	Ethical and Responsible Research Practices
Group 4	Communication & Sharing to Society	Research Communication
Group 4	Collaborative Research	Interpersonal Skills in Research
Group 4	Self-Efficacy	Personal Development as a Researcher
Group 4	Foundational Research Skills	Doing Research
Group 5	Ethical Practices	Ethical and Responsible Research Practices
Group 5	Promoting Equality in Research	Interpersonal Skills in Research
Group 5	Professional Collaboration	Interpersonal Skills in Research
Group 5	Disciplinary Knowledge	Foundational Disciplinary Knowledge
Group 5	Grit/ Self-Regulation	Personal Development as a Researcher
Group 5	Public Communication	Research Communication
Group 5	Management/Administrative	Research Administration and Management
Group 5	Technical Research Skills	Doing Research
Group 5	Discipline/ Research Vision	Thinking about Research
Group 6	Mentorship	Interpersonal Skills in Research
Group 6	[illegible] or project	Doing Research
Group 6	Public/ Broad Communication	Research Communication
Group 6	Research Communication	Research Communication
Group 6	Discipline Knowledge	Foundational Disciplinary Knowledge
Group 6	Funding	Research Administration and Management
Group 6	Research/Career Independence	Research Career Development
Group 6	Professional Skills	Interpersonal Skills in Research
Group 6	Personal/Career Knowledge Development	Research Career Development
Group 6	Inclusion	Interpersonal Skills in Research
Group 6	Attitude/Personal Development	Personal Development as a Researcher

Group	Original category	Standardized category
Group 6	Technical	Doing Research
Group 6	Collaboration	Interpersonal Skills in Research
Group 6	Ethics	Ethical and Responsible Research Practices
Group 6	Management	Interpersonal Skills in Research
Group 6	Safety	Ethical and Responsible Research Practices
Group 7	Personal Development	Personal Development as a Researcher
Group 7	Communication and collaboration	Interpersonal Skills in Research
Group 7	Analysis/technical skills	Doing Research
Group 7	Mentorship soft skills management	Interpersonal Skills in Research
Group 7	Advocacy	Interpersonal Skills in Research
Group 7	Career Development	Research Career Development
Group 7	Ethics undergrad first, postdoc last	Ethical and Responsible Research Practices
Group 7	Knowledge/ breadth	Foundational Disciplinary Knowledge
Group 7	Critical thinking	Thinking about Research
Group 7	Money	Research Administration and Management
Group 8	Collaborative Teams	Interpersonal Skills in Research
Group 8	Research Integrity	Ethical and Responsible Research Practices
Group 8	Leadership	Interpersonal Skills in Research
Group 8	Self Management	Personal Development as a Researcher
Group 8	Scientific Communication	Research Communication
Group 8	questions	Thinking about Research
Group 8	Broader Impacts	Ethical and Responsible Research Practices
Group 8	Lab Management	Research Administration and Management
Group 8	community	Research Communication
Group 8	Career Pathway	Research Career Development
Group 8	Disciplinary Knowledge	Foundational Disciplinary Knowledge
Group 8	Basic Research Skills	Thinking about Research
Group 9	Self-efficacy & Research identity	Personal Development as a Researcher
Group 9	& synthesizing new knowledge	Thinking about Research
Group 9	Communication	Research Communication
Group 9	RCR	Ethical and Responsible Research Practices

Group	Original category	Standardized category
Group 9	Interpersonal & research Management	Interpersonal Skills in Research
Group 9	Funding	Research Administration and Management
