# Supplementary material for: The comprehensive researcher development framework (CRDF): Core learning outcomes for research training
Source: PLoS One. 2025 Sep 15;20(9):e0332587. doi: 10.1371/journal.pone.0332587 (PMC12435680; doi:10.1371/journal.pone.0332587)
Supplement: Attachment — Submitted filename: Response to Reviewers_CRDF_BranchawButzAyoob.pdf [file pone.0332587.s010.pdf]

WISCIENCE
UNIVERSITY OF WISCONSIN–MADISON

445 Henry Mall
Madison, WI 53706
wiscience.wisc.edu
608.263.0598

August 22, 2025

Dear Dr. Prunuske,

Thank you for the opportunity to revise and resubmit our manuscript, "*The Comprehensive Researcher Development Framework (CRDF): Core Learning Outcomes for Research Training*." We appreciate the helpful feedback that you and the two reviewers provided and have revised the manuscript to address the concerns raised. Detailed explanations regarding how we responded to the reviewers' comments are provided in the table below.

In brief, we created a more detailed flow chart of the process we used to develop the CRDF in Figure 1 and then used the headings in this figure to create parallel subheadings in the Methods and Results sections of the paper. We believe this addresses the major concern expressed by the reviewers regarding the clarity and organization of the manuscript. We also moved the last back mapping confirmation step from the Discussion into the Results section and added a section to the Methods describing how we did the back mapping. Beyond these major rearrangements, we responded to the specific questions and suggestions from the reviewers in the table and made some minor clarifying edits in the manuscript.

Note that because we moved text between sections of the paper, the track changes version of the paper looks very busy, and the line numbering is not accurate. Consequent, the text lines listed in the reviewer response table refer to the lines in the clean manuscript without track changes.

Please contact me if any further information or clarification on these points is needed. We look forward to hearing from you.

Sincerely,

Janet Branchaw

Janet L. Branchaw, PhD
Associate Professor of Kinesiology, School of Education
Faculty Director of WISCIENCE
University of Wisconsin - Madison

Reviewer	Feedback	Response
Editor	address the reviewers' comments regarding the results section and Figure 4	See responses to individual reviewer comments below.
Editor	helpful to include a flow chart	Figure 1 was revised and is now a more detailed flow chart.
Editor	consider being more explicit about generating a tool that demonstrated consensus across disciplines	We added text indicating the CRDF transcends and demonstrates consensus across disciplines in multiple places in the article. See lines 32, 84, 590-1, and 654.
Reviewer #1	the manuscript is seriously hobbled by a lack of clarity in the writing of the Results section	The Methods and Results sections have been reorganized to be parallel, both following the detailed flow chart presented in figure 1. We believe this addresses the lack of clarity in the writing. The change necessitated flipping the numbering of Tables 3 & 4.
Reviewer #1	This is an important statement. Is there a citation?	Citations have been added in line 70.
Reviewer #1	It might be worthwhile for the authors to have a flow chart-like figure that explains their process of collecting competencies from the literature > surveying researchers > creating a refined list > open card sorting > creating categories > closed card sorting > refining the framework.	Figure 1 has been revised with more details as a flow chart. In addition, the Methods and Results sections have been reorganized to more explicitly reflect the process.
Reviewer #1	Regarding the card-sorting exercises. Is this an evidence-based approach? If so, what are the citations sorting the use of the open and closed sorting approaches.	The citation about use of card sorting activities has been added in line 327.
Reviewer #1	It feels like the Results are missing a section about their literature review.	A section about the literature search and review has been added to the Results in the new organization.
Reviewer #1	Information about the 79 outcomes prepared for the survey and the 75 that actually appeared in the survey due to a survey error belongs in the Methods section. For the results section, the authors should present the data as though there were a list of 75 outcomes since that's what there is actually data on.	The information about the 75 of the 79 outcomes that were included in the survey (4 were missing due to an error) has been moved from the Results to the Methods section.<br>The data presented in the Results now explicitly lists 75 learning outcomes in the legend of figure 4, where data collected about the importance of the learning outcomes is presented. Note the in figure 5 data from all 79 outcomes are included because all were asked about on the career stage survey questions.

Reviewer	Feedback	Response
Reviewer #1	It is not clear what figure 4 is actually supposed to show us or if it is even that important. Is this meant to communicate the % of the 75 learning outcomes judged important by the survey (segregated by discipline)? Is this really something that is important to show? What outcomes were deemed important is certainly interesting and is presented in Table 4. Figure 4's message seems to be "there were things that researchers thought were important" and that's it. Consider omitting (or explaining it better).	Figure 4 provides evidence of content validity from experts for the core learning outcomes. A statement explicitly explaining this has been added in lines 417 – 418.
Reviewer #1	Perhaps the title should be "Learning Outcomes ... by 80% or More of Overall Respondents". Also, what is the significance of the non-bolded numbers? Just that they are below 80%?	Table 4 was simplified to report only the overall importance ratings across disciplines and the bolded numbers were removed.
Reviewer #1	Are the details about what categories the relatively low scored (variable importance) outcomes really needed here. It distracts from the subsequent report about the higher scoring 50 outcomes.<br>I'm completely confused by this section. "Some variation" is reported by discipline but not what those differences are or whether they were statistically different. Then ratings were combined to minimize those differences. If there really are differences between disciplines, then it seems like something worth talking about and maybe analyzing with distribution stats. If not, then just talk about the combined data. Also, the last sentence presents a series of numbers in parentheses. Are these the learning outcomes in Table 1? If so, the format does not correspond (there are no 3.01 or 4.04 in Table 1).<br>Again, why this discussion on what wasn't highly scored.	We agree with the reviewers' comments and have followed their recommendation to remove the details about possible small disciplinary differences and to focus on the main findings.
Reviewer #1	Shouldn't the authors refer to Table 5? Also, I'm not sure that Tables 5 & 6 are in the right order. Or Table 5 is confusing. The process of going to phase 1 to phase 2 to the final 8 set of categories is shown in table 5, but the process of getting to the last set of 8 categories is based on data in Table 6. Either this needs to be explained better in the text or the order of the tables should be changed.	Table 5 precedes table 6 because table 5 shows the results of the first (open) card sort and table 6 the results of the second (closed) card sort. Table 5 also shows the final categories, which were finalized based on the second (closed) card sort results. To clarify this, the table 5 column headers were modified, and the text has been modified to more explicitly describe what is in each table using "open" and "closed" card sorting (rather than first and second phase).

Reviewer	Feedback	Response
Reviewer #1	I'm not sure I understand this approach of dealing with the learning outcomes that showed weak consensus. It sounds arbitrary, but I suspect it was not. Two potential reasons for lack of consensus were that there were discipline-specific differences in which researchers thought these outcomes should be placed. Another is that the outcome could be placed in multiple categories because it was interpreted differently by different groups. If the latter, then perhaps the right approach by the authors would be to "split" the outcome into two outcomes with each reflecting the category that researchers matched them to. An interesting follow-up experiment would be to repeat the forced choice supporting and see if the split/renamed categories got sorted as the authors predicted/assumed.	The explanation for how we dealt with the learning outcomes for which there was low consensus in the closed card sorting exercise has been clarified in lines 534 – 539 by adding more detail about the decision-making process.
Reviewer #1	How were the Table 7 data generated. I didn't see anything in the Methods that might explain it. Also, what exactly is it saying? Is this mapping how often a given outcome was discussed in the literature reviewed? Finally, doesn't this belong in the Results section?	A section was added to the Methods "Back Map Source Framework Elements to Learning Outcomes and Categories to Confirm Coverage" to describe this process (lines 360 – 365), and Table 7 was moved from the Discussion to the Results section.
Reviewer #1	It might be worth stating that the CDRF is meant to represent a relative consensus across disciplines.	Great suggestion! We added text indicating the CRDF transcends and demonstrates consensus across disciplines in multiple places in the article. See lines 32, 84, 590-1, and 654.
Reviewer #1	The use of the CRDF to increase transparency is an important point. Could the authors discuss how to operationalize that (similar to how they discussed using the CRDF as an assessment tool in the subsequent section). Placed in a student handbook? Part of a program/mentor/student compact? Something else?	Specific examples of how the CRDF can be used for and with research mentees have been added in lines 617 – 620.
Reviewer #1	This is somewhat discussed in the limitations section, but regarding the data from the Arts & Humanities. How confident are the authors regarding this data given the small sample size? Perhaps these 5 are in a post-grad research career because they had this research training in their undergrad years.	We are confident in the Arts & Humanities data and have therefore included it, but because of the small sample size we are reluctant to draw sweeping conclusions from it. We believe that acknowledging and discussing it in the Limitations section is the best way to convey this and have elaborated on what is written there to be more explicit. See lines 644 – 645.
Reviewer #2	Consistency or simplification of the headings would be helpful. For example, the methods sections has STEPS 1-4, with STEP 1 having four lettered subsections; the results section has two main subsections and one of those subsections lists the two phases of card sorting; and limitations, conclusions, and future directions are all short main sections.	Figure 1 has been revised with more details as a flow chart outlining the process we used and headings (rather than numbers and letters) have been used in the revised Methods and Results sections.

Reviewer	Feedback	Response
Reviewer #2	Somewhere in the manuscript it would be helpful to note the geographic comprehensiveness of the framework. For example, were most of the frameworks used in the literature review from the United States (or from the US and Europe) – or was there global representation from where the frameworks were developed? Is researcher development often done similarly globally, or is this framework likely most applicable to US researcher development?	34% (19) of the source frameworks were developed outside the United States, including: Iran, Germany (2), United Kingdom (3), South Africa (2), Turkey, Canada (2), Peru, Australia (3), Netherlands, India, China, and Hong Kong. A statement about this was added to the Results in lines 375 - 376 and in the discussion in lines 570 – 573.
Reviewer #2	More detail in the captions would be helpful so that the tables and figures could stand alone.	We reviewed the captions and feel confident that they convey all the information needed to interpret the tables and figures. It is unclear what the reviewer means by “stand alone.” We do not think it necessary to replicate the text from the Methods and Results sections in the captions.
Reviewer #2	There are some superscripts in Table 6, and the descriptions of what those superscripts represent is missing (i.e., there are not footnotes below Table 6).	The superscript notations are now referenced at the bottom of the table.
Reviewer #2	Table 7 has a column titled “framework names,” but the items listed seem to be the article titles.	The column title has been changed to “Framework Article.”
Reviewer #2	Table 1 lists the learning outcomes as 1.1, 1.2, 1.3…, and the text of the manuscript adds a “0” before the learning outcomes below 10 (e.g., 3.01 on line 439).	The learning outcome numbers have been updated to include the 0 in those below 10 (e.g., 1.1 is now 1.01).
